# Skull variation in Afro-Eurasian monkeys results from both adaptive and non-adaptive evolutionary processes

**DOI:** 10.1038/s41598-022-16734-x

**Published:** 2022-07-22

**Authors:** Lauren Schroeder, Sarah Elton, Rebecca Rogers Ackermann

**Affiliations:** 1 Department of Anthropology, University of Toronto Mississauga, Mississauga, ON L5L 1C6 Canada; 2 Department of Anthropology, Durham University, Dawson Building, South Road, Durham, DH1 3LE UK; 3 Department of Archaeology, University of Cape Town, Rondebosch, 7701 South Africa; 4 Human Evolution Research Institute, University of Cape Town, Rondebosch, 7701 South Africa

**Keywords:** Biological anthropology, Evolutionary theory

## Abstract

Afro-Eurasian monkeys originated in the Miocene and are the most species-rich modern primate family. Molecular and fossil data have provided considerable insight into their evolutionary divergence, but we know considerably less about the evolutionary processes that underlie these differences. Here, we apply tests developed from quantitative genetics theory to a large (n > 3000) cranio-mandibular morphometric dataset, investigating the relative importance of adaptation (natural selection) and neutral processes (genetic drift) in shaping diversity at different taxonomic levels, an approach applied previously to monkeys of the Americas, apes, hominins, and other vertebrate taxa. Results indicate that natural selection, particularly for differences in size, plays a significant role in diversifying Afro-Eurasian monkeys as a whole. However, drift appears to better explain skull divergence within the subfamily Colobinae, and in particular the African colobine clade, likely due to habitat fragmentation. Small and declining population sizes make it likely that drift will continue in this taxon, with potentially dire implications for genetic diversity and future resilience in the face of environmental change. For the other taxa, many of whom also have decreasing populations and are threatened, understanding adaptive pressures similarly helps identify relative vulnerability and may assist with prioritising scarce conservation resources.

## Introduction

While much phenotypic evolution is adaptive^1^, genetic drift also has an influential role in phenotypic differentiation^2^. Morphological data from a range of organisms including deer mice^3^, western chipmunks^4^, toads^5^, bats^6^, American monkeys^7,8^, extinct armadillo^9^, apes^10^, humans^11–14^, and extinct hominins^15–19^ have been studied to evaluate the relative roles of adaptive versus neutral evolutionary processes shaping population divergence. These investigations have used approaches derived from evolutionary quantitative genetics, with genetic drift as a null hypothesis ^20–22^. Some confirm the importance of natural selection in diversification (e.g., for *Tamias* chipmunks^4^) whereas others point to genetic drift as a cardinal differentiating force (e.g., in early *Homo*^16^), highlighting the lineage-specific nature of organismal divergence.

**Figure 1:**
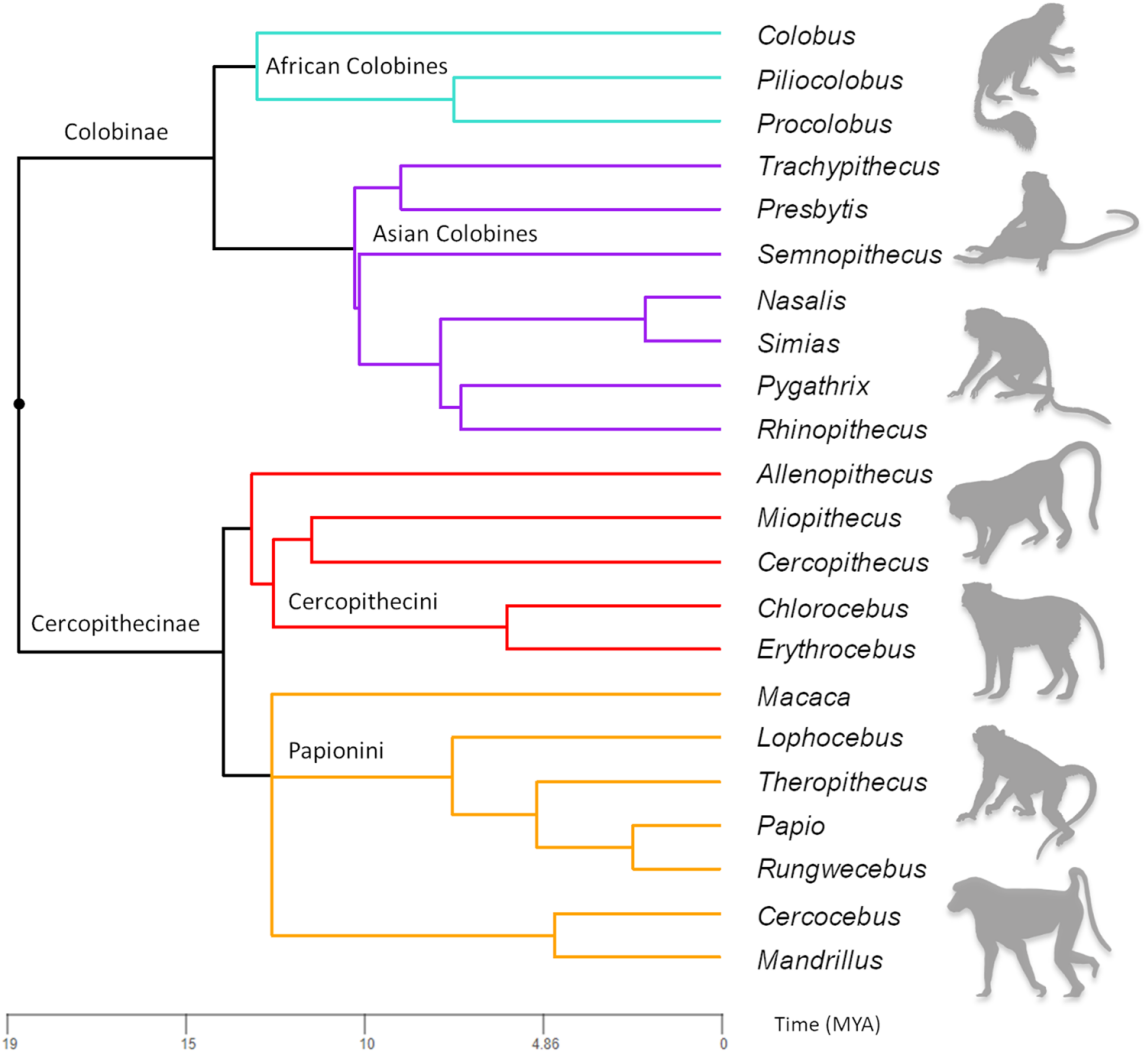
Phylogenetic tree depicting relationships between Cercopithecidae genera. Branch lengths are proportional to time (in millions of years [MYA]). African colobines are depicted in turquoise, Asian colobines in purple, guenons in red, and papionins in orange. Tree was constructed using a newick file downloaded from timetree.org^77^. Silhouettes taken from Phylopic.org. Image of *Cercopithecus* by Kai R. Casper (https://creativecommons.org/licenses/by/3.0/).

Of most relevance here, a large-scale cranial analysis across American monkeys (parvorder Platyrrhini) differentiated between selection and drift at several levels in a taxonomic hierarchy; across species within genera, to families within superfamilies^8^. In contrast, the evolution of the Afro-Eurasian monkeys (Family Cercopithecidae), the most species rich modern primate family^23^, has not been investigated in this manner. Comprising 23 extant genera and 152 extant species^24^, alongside at least 14 extinct genera and tens of species known only from the fossil record, Afro-Eurasian monkeys originated in the Miocene (although, some have proposed an earlier origin in the Oligocene^25,26^), and are split into two subfamilies, the Cercopithecinae and the Colobinae, which also diverged in the Miocene, with molecular data indicating that the two modern radiations initiated at least ca.11–12 Ma^27^ (see ^28^ for evidence of divergence dates that predate this interval). Although some modern taxa, such as Allen’s swamp monkey (*Allenopithecus nigroviridis*)*,* originated quite early in these radiations, much of the species diversity we see today evolved in the Pliocene and Pleistocene (Fig. 1).

It has been hypothesised (e.g.,^29,30^) that climatic fluctuations in the Plio-Pleistocene shaped some of the taxonomic diversity of Afro-Eurasian monkeys, through expansion and contraction of suitable habitats. These habitat pockets may be equivalent to isolates, with little gene flow with other populations^31^. Thus, differentiation in some lineages may have been driven not by selection but by stochastic processes^32^. However, there has been little formal investigation of this, especially with skeletal data (but see ^33^ for discussion of the possible influence of drift on facial colouration in primates). Work on morphological divergence in other primate groups (e.g.,^8,16^) indicates that drift is more influential at lower taxonomic levels, after initial niche filling has taken place and small population effects may be more influential. At higher taxonomic levels, it is more likely that adaptive signatures will be detected. These levels contain a myriad of species that have diverged in a multitude of ways, including adaptive radiation, rapid niche filling, and strong directional selection.

Here we investigate whether this is the case, examining skull morphology at various hierarchical taxonomic levels (Family, Subfamily, Tribe) across Afro-Eurasian monkeys. Investigating this with skeletal data is particularly important as it opens up a window for interpreting the fossil past (sensu^15,16^), which has the potential to elucidate how factors like climatic change, refugia, and genetic drift impact primate communities over time. Clarifying the relative roles of drift and selection in creating Afro-Eurasian monkey diversity can also aid in conservation planning (sensu^34^), as the varied evolutionary history of species (including the influence of evolutionary processes on variation) may impact their ability to respond to rapid environmental change. In light of the factors mentioned above, we predict that selection will be the dominant evolutionary process shaping diversity across these monkeys, with the exception of lineages (i.e., African colobines, guenons) with taxa that may have been “isolated” in refugial forest pockets in the Plio-Pleistocene, or on southeast Asian islands (Asian colobines), and thus are more likely to have differentiated via drift.

## Results

The null hypothesis of evolution by multivariate genetic drift was evaluated using two complementary tests (detailed below in Methods). These tests, based on theory from evolutionary quantitative genetics^7,20,35^, assess proportionality among between- and within-population variance, as well as correlations among the between-population principal components. If significant correlations are detected, or deviations from proportionality are observed, genetic drift can be rejected. This is usually then interpreted as being indicative of natural selection differentiating groups. While it is important to note that there are other factors that may influence the proportionality of between- and within-population variation such as phenotypic plasticity (e.g.,^36^), the framework used here, which includes direct tests of selection (specifically “co-selection” as defined in^8^), provides a clearer link to selection when genetic drift is rejected. Analyses were conducted hierarchically, first across all Afro-Eurasian monkeys, and then within each of the subfamilies, Colobinae and Cercopithecinae. Below these levels, within Colobinae, we also performed separate tests within Asian and African taxa; and within Cercopithecinae, we performed separate tests at the Tribe level, separating guenons and papionins. This approach allows us to first assess whether selection is acting across all Afro-Eurasian monkeys, and if so, then we can drill down to determine whether it is widespread or limited to certain groups or levels in the taxonomic hierarchy. Tests were carried out on the full set of 62 skull traits, as well as separately for the cranium and mandible, in order to further localize any effects.

**Figure 2:**
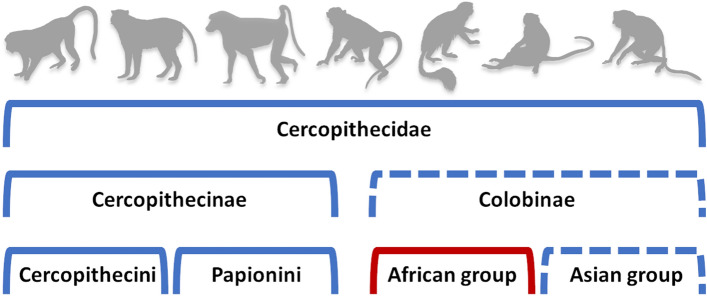
Summary of evolutionary processes across Cercopithecidae as detailed in Table 1. Blue indicates rejection of drift (and possibly diversification by natural selection), and red specifies groups for which the null hypothesis of genetic drift has not been rejected. Dashed lines depict levels in the phylogeny with varying genetic drift versus selection results for the full skull, cranial, and mandibular analyses. Silhouettes taken from Phylopic.org. Image of *Cercopithecus* by Kai R. Casper (https://creativecommons.org/licenses/by/3.0/).

Table 1 summarises the results of regression and correlation tests for each analysis using raw data. These results are also visualized in Fig. 2. Results for analyses conducted on log-scale ratio data are presented in Supplementary Table S1. These results are discussed in some detail below, however we focus more on interpretations of analyses of the raw data as this is comparable to the strategy in Marroig and Cheverud^8^. Genetic drift is labelled as rejected in the regression tests when the p-value is below 0.05, as possibly rejected when it is between 0.05 and 0.10, and as not rejected when it is greater than 0.10. Because this is a conservative test (i.e., it is difficult to reject drift when few taxa are being compared), we consider “possibly rejected” to be a fairly strong indication that drift is rejected, though it is not definitive (see discussion in^16^). In the correlation tests, if any significant correlations were detected using a Bonferroni correction, genetic drift was rejected. Significant correlations that do not reach the Bonferroni criterion (p < 0.05) are also noted. All principal component (PC) scores, PC correlations, and regression plots are provided in the Supplementary Information.

**Table 1:** Results of regression analysis of between- versus within-group variance, and between-group principal component (PC) correlation analysis as tests for genetic drift^#^.

Analysis	Regression test					Correlation test
	Rejection of drift?	Slope (95% Confidence Interval)	R^2^	t-statistic	p-value	Significantly correlated PCs*
**Full skull**						
**Family**						
Cercopithecidae	Yes	1.163 (1.056–1.270)	0.887	3.039	0.004	PC1–PCs 4,6,20; PC2–PCs 3,16; PC3–PCs 7,16; PC4–PCs 6,7,20; PC5–PC16; PC6–PC20; PC7–PC19
**Subfamily**						
Cercopithecinae	Yes	1.240 (1.110–1.370)	0.859	3.696	< 0.001	PC1–PCs 4,7; PC4–PC7
**Tribe**						
Papionini	Yes	1.179 (1.027–1.330)	0.801	2.359	0.022	PC2–PC3
Cercopithecini	Yes	1.278 (1.145–1.411)	0.860	4.174	< 0.001	*(PC1*–*PC4)*
**Subfamily**						
Colobinae	No	1.045 (0.938–1.151)	0.865	0.841	0.517	PC1–PC2
**Region**						
Asian colobines	No	1.036 (0.910–1.162)	0.819	0.568	0.572	*(PC1*–*PC2)*
African colobines	No	1.162 (0.915–1.409)	0.596	1.315	0.193	None
**Cranium**						
**Family**						
Cercopithecidae	Yes	1.216 (1.092–1.341)	0.914	3.516	0.001	PC1–PCs 5,7,8; PC2–PCs 3,4,11,12; PC3–PCs 4,11,12; PC4–PC5,11,12; PC5–PC12,17; PC6–PCs 19,20; PC8–PC10; PC10–PC20; PC11–PC12; PC12–PC13; PC19–PC20
**Subfamily**						
Cercopithecinae	Yes	1.304 (1.120–1.488)	0.848	3.343	0.002	PC1–PC6,8; PC4–PC5; PC5–PC6
**Tribe**						
Papionini	Yes	1.229 (1.022–1.436)	0.796	2.236	0.031	*(PC4*–*PCs 2,5)*
Cercopithecini	Yes	1.336 (1.156–1.517)	0.859	3.774	< 0.001	*(PC1*–*PC2; PC3*–*PC5)*
**Subfamily**						
Colobinae	Possibly	1.166 (0.647–1.345)	0.825	1.882	0.068	PC1–PC3
**Region**						
Asian colobines	Yes	0.593 (0.443–0.744)	0.632	5.467	< 0.001	*(PC1*–*PC2; PC2*–*PCs 3,5; PC3*–*PC4)*
African colobines	No	1.036 (0.726–1.346)	0.554	0.237	0.814	None
**Mandible**						
**Family**						
Cercopithecidae	Yes	1.233 (1.059–1.407)	0.912	2.783	0.011	PC1–PCs 4,15,16; PC2–PC6; PC4–PC16; PC5–PC20; PC6–PC17; PC7–PC16; PC10–PC16; PC11–PC13
**Subfamily**						
Cercopithecinae	Yes	1.296 (1.031–1.560)	0.832	2.325	0.030	PC1–PC4
**Tribe**						
Papionini	Possibly	1.224 (0.981–1.467)	0.839	1.915	0.069	*(PC1*–*PC4)*
Cercopithecini	Yes	1.283 (1.055–1.512)	0.867	2.580	0.017	None
**Subfamily**						
Colobinae	No	1.046 (0.814–1.278)	0.807	0.410	0.686	*(PC1*–*PC4; PC3*–*PCs 6,7)*
**Region**						
Asian colobines	Yes	0.738 (0.501–0.974)	0.668	2.311	0.031	*(PC1*–*PC2; PC4*–*PCs 2,3)*
African colobines	No	1.421 (0.818–2.023)	0.534	1.453	0.161	None

*Full PC correlation results are provided in Supplementary Info. Italicized comparisons in parentheses are those with p-values below 0.05 that do not meet the Bonferroni criterion.

For Afro-Eurasian monkeys as a whole, both the regression and correlation analyses for raw data reject drift whether all the skull traits are assessed together, or instead analysed separately as cranium and mandible (Table 1; Supplementary Table S1). The first PC (most often associated with allometric variation) is always the most divergent in these analyses (Supplementary Figure S1), which potentially indicates a major role of selection for different body sizes. The loadings on each PC for the full skull, cranium, and mandible analyses are provided in Supplementary Tables S2.1, S2.2, S2.15, S2.16, S2.29, S2.30. The correlation analysis supports this notion, with influential morphological traits, like those related to basicranial flexion, neurocranial shape, and ramus height and depth, always co-selected with size-dominated PC1. In addition, analyses utilizing size-adjusted (log-shape ratio) data show a contrasting pattern, with no rejections of genetic drift detected in the cranial and mandibular analyses, and a potential rejection (p = 0.07) in the full skull analysis, further supporting the importance of selection for body size differentiation in the Cercopithecidae Family.

For Cercopithecinae, genetic drift was rejected or possibly rejected for all but the log-shape ratio mandibular analysis, again pointing to an important role for adaptive divergence in this clade (Table 1; Supplementary Table S1). The highest PCs in the regression analysis were related to snout morphology and size, which was co-selected with neurocranial traits in the correlation analysis (PC loadings given in Supplementary Table S2.3, S2.4, S2.17, S2.18, S2.31, S2.32). When guenons (Cercopithecini) and papionins (Papionini) were analysed separately, adaptive divergence was detected within both of these tribes. For the skulls, there is a clear indication of adaptive divergence within guenons in both log-shape ratio and raw data, and within papionins mainly for the raw data analyses, but also the log-shape ratio cranial analysis (Table 1; Supplementary Table S1).

Separate analyses of the crania and mandibles indicate that diversification of papionins largely involved adaptive changes in cranial shape and size (Table 1; Supplementary Table S1). Interestingly, the correlation analysis for papionin skulls shows a significant correlation between PC2 and PC3, indicating co-selection of cranial traits related to anterior cranial height and mandibular ramus height (see Supplementary Tables S2.7, S2.8, S2.21, S2.22, S2.35, S2.36 for PC loadings). In addition, in papionins there is a significant correlation (p = 0.02) between PC1 and PC5, suggesting co-selection between snout and neurocranial morphology (although this correlation did not reach significance according to Bonferroni criterion; Supplementary Table S3.7). The log-shape ratio mandibular analysis suggests that mandibular shape in papionins may have arisen through drift (Supplementary Table S1). For guenons, the correlation and regression analyses present somewhat contradictory results. There are no significant correlations, and therefore no rejection of drift in the mandibular correlation analysis (Table 1). However, there are signs of size-related adaptive diversification across the cranium which mirror that of the signal from the Cercopithecinae full skull correlation analysis (co-selection of snout morphology and neurocranial height; Supplementary Table S2.23), although these significant correlations do not reach the conservative Bonferroni criteria.

Within the subfamily Colobinae, regression analyses of the skulls of all colobines, as well as African and Asian colobines separately, do not reject genetic drift for either log-shape ratio or raw data indicating that selection was a lesser player for this subfamily as a whole (Table 1; Supplementary Table S1). The correlation analyses, however, do suggest a possible rejection of drift at the subfamily level, and in the Asian group (Table 1). Traits that are co-selected are related to basicranial flexion and mandibular ramus height at the subfamily level, with the Asian group demonstrating higher PCs in mandibular traits (loadings in Supplementary Table S2.11, S2.12). When crania and mandibles are analyzed separately, a similar pattern emerges. There is no rejection of drift in African colobines, while Asian colobines have a more widespread adaptive signature across log-scale ratio and raw data for both crania and mandibles (Table 1; Supplementary Table S1). This is reflected in the correlation analyses too, where significant correlations are detected consistently in only the Asian group (Table 1), however this could also be a reflection of the small number of taxa being compared in the African group.

## Discussion

Afro-Eurasian monkeys are found in Africa and Asia (and in the past, Europe), distributed across multiple biomes including many different forest types, woodlands, grasslands, occasionally deserts, as well as multiple elevations, from mountains to shorelines, with some species extending out of the tropics into temperate latitudes. The diversity of biomes and habitats exploited by the Afro-Eurasian monkeys is echoed by their ecological, behavioural, and morphological diversity. The high species diversity of modern Afro-Eurasian monkeys has been attributed to moderately increased speciation over time alongside decreased extinction rates, leading to high net diversification rates^23^. Our analyses show that this diversification is driven by a combination of adaptive and neutral processes, at least when considering cranio-mandibular form.

Starting with Afro-Eurasian monkeys as a whole, the analyses indicate that diversification was not neutral (Table 1), possibly pointing to adaptive radiation into distinct niches, consistent with exploitation of different habitats and the diversification of body sizes in the family (as supported by the size-adjusted results in Supplementary Table S1). Afro-Eurasian monkey taxa differ widely in body mass, ranging from less than 1 kg to greater than 40 kg, and in the past even larger^37,38^. Body size is fundamental to an organism’s ecology and biology, linked closely with life history, diet, and substrate use. Size is also labile, and change in size could be a ‘line of least evolutionary resistance’ like that seen in American monkeys^39^, a relatively easy way for a radiation to diversify into different niches. Many species also exhibit considerable sexual dimorphism in body mass, which is likely to have been shaped by sexual selection^40^, and in some groups possibly after lineages were established, as indicated by a recent study showing variable rates of body mass evolution in Afro-Eurasian monkeys^41^. Although we did not analyse allometric patterns in this study, the importance of size identified here may also indicate that allometric relationships between body size and skull traits could have constrained the action of evolutionary processes in certain taxa and limited morphological variability, which is a promising avenue for future research (e.g.,^42,43^). Climatic niche shifts may also have promoted diversification in Afro-Eurasian monkeys^44^ into diverse biomes and habitats, and our findings of selection rather than drift are consistent with adaptation to (rather than neutral occupation of) these biomes/habitats, at least for the Family as a whole.

Of course, this does not mean that selection was uniform across all taxa, just that there is selection acting within the clade. When considering the next hierarchical level, the subfamilies, a more complex pattern emerges. Diversification of papionins largely involved adaptive changes in cranial size and shape. Evidence also exists for co-selection of cranial traits related to anterior cranial height and mandibular ramus height, and possible co-selection between snout and neurocranial morphology. This finding highlights the variable muzzle expression in the group, and given the probable importance in behavioural signalling within the papionins^45^, suggests that the adaptation detected here may be a combination of sexual and natural selection. There is also some indication that mandibular form and shape in papionins may have arisen through drift, which may be explained by the relatively generalised and eclectic diets in many members of the clade, and correlated weak dietary selective pressures.

For guenons, the pattern of size-related adaptive diversification in the cranium (but not the mandible) is consistent with phenetic analyses that highlight the fundamental role that size plays in the tribe’s variation^46^. Nonetheless, the rejection of drift for the guenons runs contrary to expectations that isolation in refugial forest fragments may have promoted neutral diversification. Much guenon species diversity is contained within a single genus, *Cercopithecus*, which shows high rates of species diversification^47^. It is common to find *Cercopithecus* in multi-species aggregations^48^. *Cercopithecus* species are quite similar in hard-tissue cranial morphology^47^, with most morphological differentiation being in soft tissue features such as facial colouration and pelage^46,49,50^. Subtle cranial variations appear to track specific differentiation based on soft tissue^46^. Our expectation of neutral diversification may not have been supported because those soft tissue facial features, which may have evolved when groups were isolated from one another in forest refugia, have been under selection subsequently to minimize hybridisation between different taxa^50^. Alternatively, the unexpected result may reflect the scale at which the analysis was performed: following the early divergence of the basal *Allenopithecus*, then *Miopithecus* clades, guenons are divided into two main clades, the arboreal genus *Cercopithecus* and the terrestrial genera most commonly including *Chlorocebus*, *Erythrocebus* and *Allochrocebus* (although the taxonomy of the terrestrial clade is dynamic). Molecular analyses indicate a single move to terrestriality within guenons^51^ and ecomorphological research on the guenon postcranium suggests that within more general stochastic variation, there are some clear adaptive signals in terrestrial guenon differentiation^52^. Thus, our analysis may be picking up the selection inherent in the terrestrial transition. Sampling limitations prevent us from subdividing our analyses further and exploring signals at a lower taxonomic level, but it may be an interesting line of future research, not only for guenons but also for the papionin macaques, some of which are island endemics and thus may have been more subject to stochastic evolutionary processes than other members of the tribe.

Turning to the subfamily Colobinae, indications of selection appear to be primarily in the Asian taxa, where there is a widespread adaptive signature across the skull, with strong selection for neurocranial traits and basicranial flexion, which were in turn co-selected with mandibular ramus height. This is consistent with the divergent morphology of colobines, a generally short-faced group of monkeys with globular frontal squama and high cranial vaults^53^. Importantly, although the colobines *Nasalis*, *Simias* and multiple species of *Presbytis* and *Trachypithecus* are found in island Southeast Asia^54^, and therefore we might expect a primary role for drift, there is no evidence that drift played a role in their diversification, in contrast to the patterns seen in the African clade.

The potential importance of drift in African colobine skull evolution is consistent with their evolutionary history occupying forest patches that probably decreased in size with climatic cooling, reducing gene flow between groups and promoting stochastic evolutionary processes in allopatry^29,30^. In particular, the complexity of red colobus (*Piliocolobus*) taxonomy (reviewed in ^55^), the probable presence of a “hybrid swarm” of some taxa^56^, and the difficulties of assigning clear morphological boundaries between taxa^57^ suggests an evolutionary history that is far from straightforward. There is equivocal evidence for niche differentiation in red colobus and it is possible that *Piliocolobus* is a non-adaptive radiation^32^. Evolutionary analyses of the extant African colobines as a whole (*Colobus*, *Piliocolobus* and *Procolobus*) showed no “early burst” of evolution and little ecological opportunity^58^, expected under classic adaptive radiation models, which lends support to the findings of neutral morphological evolution in our current study. Extant African colobines are confined to tropical forest, whereas Asian colobines are much more ecologically diverse (sensu ^59^), which implies the potential for more adaptive differentiation. Indeed, the contrasting patterns in the African and Asian colobines are consistent with other work that has found that diet-related ecological opportunity was unlikely to have driven morphological diversification in African colobines^58^. In the absence of this, as identified in our study, traits may evolve stochastically. This is not to say, however, that no adaptive diversification has occurred in African colobines. As noted by Tran^58^, future studies need to consider the processes by which the large extinct African colobines, many of which were more terrestrial than modern taxa, evolved and diverged.

As a whole, the findings from our study of Afro-Eurasian monkeys broadly mirror those of Marroig and Cheverud’s work on American monkeys^8^ in that the hypothesis of drift could be rejected at the family level. This reiterates the importance of selection in primate morphological evolution. However, there are some key differences. Evidence for adaptive diversification was found in American monkeys at all taxonomic levels above the species^8^, whereas in Afro-Eurasian monkeys, neutral evolutionary processes may have been at play in skull divergence within the subfamily Colobinae, and in particular the African colobine clade. Nevertheless, this result is consistent more generally with work on morphological divergence in other primate groups (e.g.^8,10^) indicating that drift is more influential at lower taxonomic levels. In hominoids, the extant catarrhine sister clade of cercopithecids, stabilizing selection was the predominant force in cranial evolution, again emphasizing the importance of selection, although drift could not be rejected in the divergence of mountain gorillas from its conspecifics, and orangutans from African apes^10^. Both these taxa were likely to have experienced small population sizes due to changes in habitat availability caused by climate change or anthropogenic factors, with consequent stochastic evolution. Here, there are clear parallels with the African colobines. Also within apes, drift could not be rejected for the divergence of southeast Asian *Hylobates* from its ancestor with siamangs, possibly because selective pressures relaxed in an insular environment, resulting in the evolution through drift of a smaller-bodied gibbon form^10^. We found no evidence of a similar role for insularity in southeast Asian monkeys.

These results, in combination with those from previous studies, provide information about evolutionary process that has potential implications for conservation biology. Three of the catarrhine taxa identified above as being subject to non-adaptive evolutionary processes possibly because of habitat fragmentation and loss of diversity—African colobines, orangutans, and the mountain gorilla—have declining populations, with most species listed at least as vulnerable but more commonly endangered or critically endangered^24^. Small and declining population sizes make it likely that drift will continue in these taxa, which could have dire implications by reducing within-group variation and thus evolutionary resilience in the face of further environmental change. This is not to say, however, that taxa with adaptive signals fare much better: chimpanzees and the vast majority of Asian colobines, for example, also have decreasing populations and are threatened^24^. However, understanding past selection and how it continues to shape such primate communities may provide some insight into which groups are more or less adaptable in the face of environmental change, helping conservationists to prioritise resources that are often quite limited^34^. Finally, and exemplified by studies of African red colobus monkeys, better understanding of evolutionary process, especially alongside understanding the variation across a taxon’s range, can help to unravel taxonomic complexities, assist in protecting possible cryptic diversity and help ensure that meaningful taxonomic units are recognised in conservation planning^55,60^.

## Methods

### Morphological dataset

**Figure 3:**
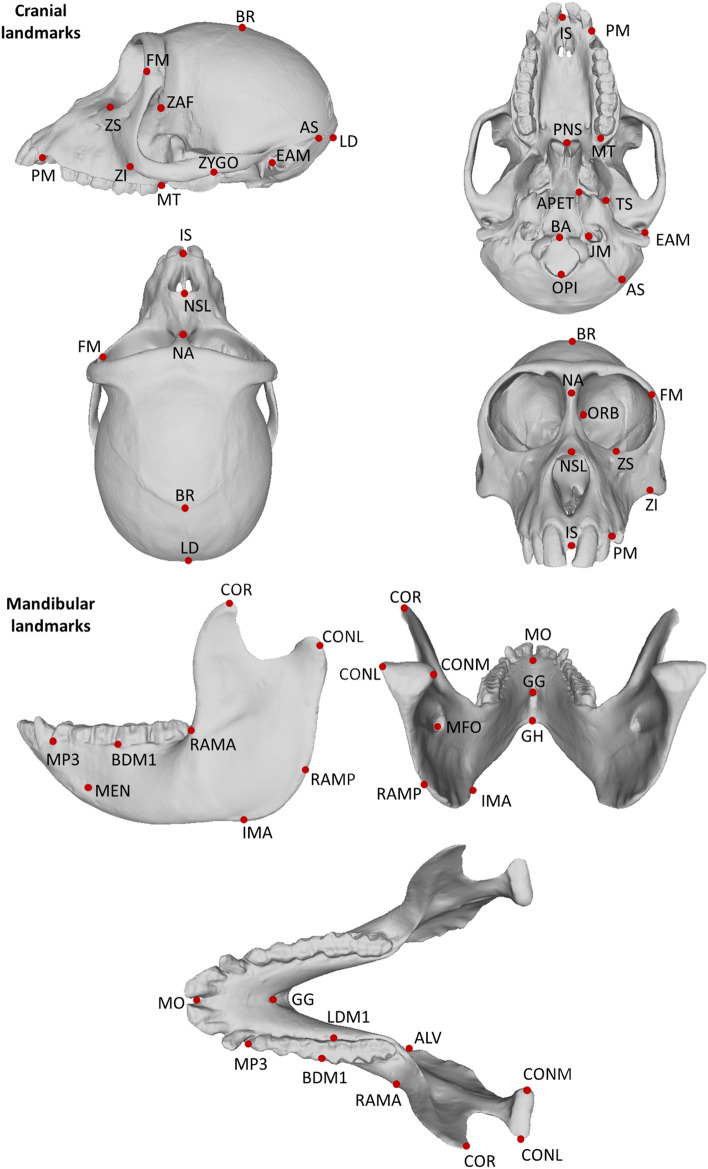
Landmarks recorded on Cercopithecidae crania and mandibles. Landmark descriptions and definitions of abbreviations given in Table 3. Scan images taken from scan of a *Macaca mulatta* individual (Specimen number: IMNH r389) downloaded from www.Morphosource.org, Duke University. Idaho Museum of Natural History provided access to these data. The collection of which was funded by Rick Carron Foundation.

We used a large morphometric dataset to analyse the cercopithecid skull (matched cranial and mandibular specimens, n = 3407), cranium (n = 3556) and mandible (n = 3711). Our sample comprised 80 cercopithecid species across 21 genera, and included males and females. Detailed sample composition is given in Table 2. Specimens were housed in the collections of the National Museum of Natural History (Washington, USA), the American Museum of Natural History (New York, USA), the Museum of Comparative Zoology of Harvard University (Cambridge, USA), the Field Museum of Natural History (Chicago, USA), the Museo di Storia Naturale, Università di Pavia (Pavia, Italy), the Museum für Naturkunde of the Humboldt University (Berlin, Germany), Staatliches Museum für Naturkunde Karlsruhe (Karlsruhe, Germany), Senckenberg Natural History Museum (Frankfurt am Main, Germany), Anthropology Institute and Museum (AIM) at the University of Zurich (Zurich, Switzerland), the Zoologische Sammlung des Bayerischen Staates (Munich, Germany), the Royal Museum for Central Africa (Tervuren, Belgium), the Hunterian Museum of the Royal College of Surgeons (London, UK), the Natural History Museum London (London, UK), and the Powell-Cotton Museum (Birchington, UK). Some data were also derived from field collections^63^. Data were collected, on the left side of each specimen to avoid redundant information, by Andrea Cardini using a 3D MicroScribe. In their original geometric morphometric form, much of the data have been used in previous published works^46,61–65^. In this current study, a total of 62 interlandmark distances (39 cranial and 23 mandibular traits) were extracted from 21 cranial landmarks and 15 mandibular landmarks (Table 3; Fig. 3). The 39 cranial traits were carefully chosen to be directly comparable to previous analyses of platyrrhines^8^. Additionally, although we are aware of the power of geometric morphometric data (raw and Procrustes transformed landmarks) for visualization and analyses of shape (e.g.,^66–68^), here we chose to use interlandmark distances to mirror the approaches of previous studies that have utilized the particular quantitative genetic framework we used in our study^20^. Analyses were conducted on both raw and log-shape ratio data. The latter is size-adjusted, and is obtained by first dividing each individual by its geometric mean (of all traits in each analysis), and then logging this measure (following^69,70^). For subsequent principle components analyses using log-shape ratio data, one degree of freedom is lost due to scaling.

**Table 2:** Cercopithecidae sample sizes for each analysis in this study.

Subfamily	Tribe/region	Genus	Full skull analysis sample size (Female/Male/Unknown)	Cranial analysis sample size (Female/Male/Unknown)	Mandibular analysis sample size (Female/Male/Unknown)
Cercopithecinae	Cercopithecini	*Allenopithecus*	21 (8/13/0)	22 (9/13/0)	22 (8/14/0)
Cercopithecinae	Cercopithecini	*Allochrocebus*	43 (19/24/0)	45 (19/26/2)	46 (20/26/0)
Cercopithecinae	Cercopithecini	*Cercopithecus*	786 (366/413/7)	801 (372/421/8)	834 (387/440/7)
Cercopithecinae	Cercopithecini	*Cholorocebus*	382 (156/224/2)	409 (170/237/2)	419 (167/247/5)
Cercopithecinae	Cercopithecini	*Erythrocebus*	37 (12/24/1)	37 (12/24/1)	37 (12/24/1)
Cercopithecinae	Cercopithecini	*Miopithecus*	36 (18/18/0)	36 (18/18/0)	42 (21/21/0)
Cercopithecinae	Papionini	*Cercocebus*	139 (63/74/2)	142 (65/75/2)	149 (66/81/2)
Cercopithecinae	Papionini	*Lophocebus*	93 (45/45/3)	98 (47/48/3)	97 (47/46/4)
Cercopithecinae	Papionini	*Macaca*	865 (365/486/14)	895 (377/504/14)	943 (391/536/16)
Cercopithecinae	Papionini	*Mandrillus*	66 (31/35/0)	68 (32/36/0)	80 (35/45/0)
Cercopithecinae	Papionini	*Papio*	427 (99/325/3)	454 (109/340/5)	496 (120/373/3)
Cercopithecinae	Papionini	*Theropithecus*	30 (14/16/0)	32 (14/18/0)	36 (15/21/0)
Colobinae	Africa	*Colobus*	140 (72/63/5)	140 (72/63/5)	142 (72/65/5)
Colobinae	Africa	*Piliocolobus*	255 (152/100/3)	289 (180/106/3)	277 (169/105/3)
Colobinae	Africa	*Procolobus*	26 (17/7/2)	26 (17/7/2)	28 (19/7/2)
Colobinae	Asia	*Nasalis*	21 (10/11/0)	21 (10/11/0)	23 (12/11/0)
Colobinae	Asia	*Presbytis*	13 (7/6/0)	14 (7/7/0)	13 (7/6/0)
Colobinae	Asia	*Pygathrix*	4 (2/2/0)	4 (2/2/0)	4 (2/2/0)
Colobinae	Asia	*Rhinopithecus*	3 (0/3/0)	3 (0/3/0)	3 (0/3/0)
Colobinae	Asia	*Semnopithecus*	6 (3/3/0)	6 (3/3/0)	6 (3/3/0)
Colobinae	Asia	*Trachypithecus*	14 (7/7/0)	14 (7/7/0)	14 (7/7/0)
		Total	3407	3556	3711

**Table 3:** Description of landmarks used in this study.

Landmark description	Abbreviation
Cranium*	
Prosthion (anteroinferior point on projection of premaxilla between central incisors)	IS
Anteriormost point of canine alveolus	PM
Posterior midpoint onto alveolar margin of M3	MT
Tip of posterior nasal spine	PNS
Meeting point between the basisphenoid, basioccipital and petrous part of temporal bone	APET
Meeting point of petrous part of temporal bone, alisphenoid and base of zygomatic process of temporal bone	TS
Anterior tip of the external auditory meatus	EAM
Medial extremity of jugular foramen	JM
Basion: anterior-most point of foramen magnum	BA
Opisthion: posterior-most point of foramen magnum	OPI
Most lateral meeting point of mastoid part of temporal bone and supraoccipital	AS
Rhinion: most anterior midline point on nasals	NSL
Nasion: midline point on fronto-nasal suture	NA
Frontomalare orbitale: where frontozygomatic suture crosses inner orbital rim	FM
Zygo-max superior: antero-superior point of zygomaticomaxillary suture taken at orbit rim	ZS
Zygo-max inferior: antero-inferior point of zygomaticomaxillary suture taken at maxillary margin	ZI
Centre of nasolacrimal foramen (fossa for lacrimal duct)	ORB
Zygo-temp inferior: infero-lateral point of zygomaticotemporal suture on lateral face of zygomatic arch	ZYGO
Meeting point of zygomatic arch, alisphenoid and frontal bone	ZAF
Bregma: junction of coronal and sagittal sutures	BR
Lambda: junction of sagittal and lamboid sutures	LD
**Mandible**^#^	
Antero-superior point of mandible between central incisors	MO
Mesial P3: most mesial point on P3 alveolus, projected onto alveolar margin	MP3
Contact points between adjacent pre-molars/molars, projected buccally onto alveolar margin	BDM1
Posterior midpoint onto alveolar margin of M3	ALV
Contact points between adjacent pre-molars/molars, projected lingually onto alveolar margin	LDM1
Anterior ramus on oblique line of the mandible	RAMA
Superior tip of coronoid process	COR
Most lateral point on mandible condylar surfaces	CONL
Most medial point on mandible condylar surfaces	CONM
Most posterior extension of ramus	RAMP
Anterior-most point on roughening for attachment of masseter on inferior margin of the angle of mandible	IMA
Mandibular foramen	MFO
Region of insertion of genioglossus muscles (midline posterior-most point on upper ‘ridge behind incisors’)	GG
Region of insertion of geniohyoid muscles (midline posterior-most point on lower ‘ridge behind incisors’)	GH
Mental foramen (most anteriorly projecting point)	MEN

*Cranial interlandmark distances were extracted from this list as follows: IS-PM, BR-APET, MT-PNS, IS-NSL, ZAF-FM, PNS-APET, IS-PNS, TS-MT, APET-BA, PM-ZS, ZAF-BA, APET-TS, PM-ZI, ZAF-EAM, BA-EAM, PM-MT, ZAF-ZYGO, EAM-ZYGO, NSL-NA, AS-EAM, ORB-ZS, NSL-ZS, FM-ZS, LD-AS, NSL-ZI, FM-MT, BR-LD, NA-BR, ZS-ZI, OPI-LD, NA-FM, ZI-MT, ZAF-AS, NA-PNS, ZI-ZYGO, JP-AS, BR-ZAF, NA-ORB, BA-OPI.

^#^Mandibular interlandmark distances were extracted from this list as follows: MO-GG, GG-GH, GH-IMA, IMA-RAMP, RAMP-CONL, CONL-CONM, CONL-COR, COR-RAMA, RAMA-RAMP, COR-IMA, MFO-ALV, MO-MP3, MP3-BDM1, BDM1-RAMA, CONM-ALV, MFO-CONM, RAMA-GH, MP3-MEN, MEN-GH, ALV-IMA, ALV-RAMA, LDM1-BDM1, LDM1-GH.

### Quantitative genetic methodological framework

The methodological approach taken here closely follows that of Ackermann and Cheverud^7^, who evaluated the evolutionary processes contributing to craniofacial variation in tamarins (genus: *Saguinus*), as well as Marriog and Cheverud^8^, who applied these methods across American monkeys. This involves comparing between-group phenotypic variation and within-group phenotypic variation, based on an application of the Lande model^3,20–22^ in evolutionary quantitative genetics, which tests whether the morphological patterns in the data follow expected patterns of differentiation through multivariate genetic drift. This expectation is given by the equation:

$${B}_{t} = G(t / {N}_{e}),$$

where ${B}_{t}$ is the between population phenotypic variance/covariance (V/CV) matrix, $t$ is the number of generations since divergence from the ancestral population, $G$ is the additive genetic V/CV matrix, and ${N}_{e}$ is the effective population size^3,7,8^. The phenotypic within-group V/CV ($P$) matrix can be substituted for $G$, following Cheverud’s conjecture^71^, which shows that proportionality between $G$ and $P$ matrices is common. Although this substitution has been the subject of some criticism (e.g.,^72^), a recent study by Sodini and colleagues^73^ provides further support for it. Because $t$ and ${N}_{e}$ are constants for any given comparison, we are able to focus on ${B}_{t}$ and $P$ for investigating whether morphological differentiation follows a model of genetic drift.

Building on this equation, Ackermann and Cheverud^7^ developed two methods for evaluating the null hypothesis of evolution by multivariate genetic drift. The first method tests the proportionality of the between-group and within-group variation, which is expected to be proportional under genetic drift. First, a pooled across taxon within-group covariance matrix was estimated separately for each analysis. As the calculation of each covariance matrix comprised a different number of individuals, with some estimated from ~ 3000 individuals, we also performed a sampling test, using a subset of cranial analyses as a model, whereby we randomly selected 50 individuals for each covariance matrix estimate to standardize the number of individuals in each analysis (Supplementary Table S4). As the results were consistent with the original covariance estimates, we chose to perform all analyses using all individuals available. The residual covariance matrix from a MANOVA was then used to correct for sex and population structure (by genus) in each matrix. Logged within-group eigenvalues ($W$), obtained from principal components (PCs) calculated from the pooled covariance matrices, were then regressed onto logged between-group variances, calculated as the variance among group mean differences projected onto those PCs. If groups have diversified through random evolutionary processes such as genetic drift, the prediction is that the relationship between within-group and between-group morphological variation will be directly proportional (i.e., slope of regression not significantly different from 1 determined using a t-test), indicating that the pattern of variance within and between these groups are comparable. A non-proportional relationship, or rejection of drift, indicates that morphology is too variable for divergence to have occurred through random forces alone; in this case, non-random forces, such as directional selection, are likely to be at work.

The second method, used to complement and verify the results of the regression analysis, tested for significant correlations among the between-group PCs, expected to be uncorrelated under a model of genetic drift^7,8,35,74^. This was done by projecting the group means onto the PCs of each pooled within-group covariance matrix (described above), and then calculating Pearson product-moment correlations between $k - 1$ of these resultant scores ($k$ = number of taxa). If any significant correlations were detected we rejected the null hypothesis of genetic drift. For any significant correlations, we also evaluated which traits were being co-selected. No phylogenetic correction was applied in either method. Rather, we follow the approach of Marriog and Cheverud^8^ who instead emphasize the importance of monophyly in comparisons of between and within-taxon variation. As all of our comparisons utilized monophyletic groups, and controlled for population structure in covariance estimation, we consider this to meet the above criterion (especially as our focus was on broad patterns in the Cercopithecidae family).

Tests were performed using the functions “DriftTest” and “PCScoreCorrelation” in the “evolqg” package in R^75,76^.

## Supplementary Information

Supplementary Information 1.

Supplementary Information 2.

## Data Availability

The dataset used in this study is available in the Supplementary Information.
