# Supplementary material for: Skull variation in Afro-Eurasian monkeys results from both adaptive and non-adaptive evolutionary processes
Source: Sci Rep. 2022 Jul 22;12:12516. doi: 10.1038/s41598-022-16734-x (PMC9307787; doi:10.1038/s41598-022-16734-x)
Supplement: Supplementary file 1 — Supplementary Information 1. [file 41598_2022_16734_MOESM1_ESM.pdf]

Skull variation in Afro-Eurasian monkeys results from both adaptive and non-adaptive evolutionary processes

Lauren Schroeder, Sarah Elton, Rebecca R Ackermann

Supplementary Information

**Table S1.** Results of regression analysis of between- versus within-group variance, and between-group principal component (PC) correlation analysis as tests for genetic drift (log-scale ratio data) [#]	pp. 2-3
**Table series S2.** First 10 principal components for each analysis (42 tables in total).	pp. 4-68
**Table series S3.** Pearson product-moment correlation values (below diagonal) and associated p-values (above diagonal) for each analysis (42 tables in total)	pp. 69-83
**Table S4.** Results of the sampling test for covariance matrix estimation using a selection of raw cranial data. Comparative results for the full dataset are provided for comparison	p. 83
**Figure S1.** Regression scatterplots of logged between- and within-population variance across Afro-Eurasian monkeys. A) Skull raw data, B) Skull log-scale ratio data, C) Cranial raw data, D) Cranial log-scale ratio data, E) Mandibular raw data, F) Mandibular log-scale ratio data	p.84

Table S1. Results of regression analysis of between- versus within-group variance, and between-group principal component (PC) correlation analysis as tests for genetic drift (log-scale ratio data) [#]

	Regression test					Correlation test
**Analysis**	**Rejection of Drift?**	**Slope (95% CI)**	**R^2**	**t-statistic**	**p-value**	**Significantly correlated PCs***
**<u>Full skull</u>**						
**Family**						
Cercopithecidae	Possibly	1.096 (0.990-1.201)	0.88	1.819	0.074	PC1 – PCs 6,11,12,15,17; PC5 – PC19; PC6 – PCs 9,11,12,15,16,17,20; PC7 – PC12; PC8 – PC18; PC9 – PC15,17,18; PC11 – PC20; PC14 – PC15; PC15 – PCs 17,18; PC17 – PCs 18,20
**Subfamily**						
Cercopithecinae	Yes	1.332 (1.021-1.245)	0.874	2.377	0.021	PC2 – PCs 3,6,10; PC3 – PCs 6,11; PC6 – PCs 7,10,11; PC7 – PCs 8,10; PC8 – PC10
**Tribe**						
Papionini	No	1.017 (0.872-1.162)	0.77	0.239	0.812	*(PC1 – PC3)*
Cercopithecini	Yes	1.172 (1.025-1.320)	0.811	2.337	0.023	*(PC3 – PC4)*
**Subfamily**						
Colobinae	No	0.928 (0.818-1.037)	0.83	1.323	0.191	*(PC2 – PC3)*
**Region**						
Asian colobines	No	0.911 (0.771-1.051)	0.763	1.269	0.209	None
African colobines	No	0.967 (0.747-1.187)	0.567	0.298	0.767	None
**<u>Cranium</u>**						
**Family**						
Cercopithecidae	No	1.111 (0.963-1.260)	0.865	1.519	0.137	PC1 – PCs 3,7,11; PC3 – PCs 4,7,9,11; PC4 – PCs 10,11; PC7 – PCs 8,10,11,13,17; PC8 – PC13; PC10 - PCs 11,13; PC11 - PCs 14,17; PC13 - PC17
**Subfamily**						
Cercopithecinae	Possibly	1.134 (0.984-1.283)	0.868	1.812	0.782	PC1 – PCs 2,3,6,7,11; PC3 – PCs 6,7,11; PC6 – PC11
**Tribe**						
Papionini	Possibly	1.134 (0.977-1.291)	0.856	1.733	0.091	*(PC1 - PCs 2,3)*

Cercopithecini	No	1.096 (0.893-1.299)	0.769	0.957	0.345	*(PC3 – PC4)*
**Subfamily**						
Colobinae	No	1.010 (0.866-1.153)	0.849	0.134	0.894	*(PC2 – PC5; PC3 - PC6)*
**Region**						
Asian colobines	Yes	0.693 (0.568-0.817)	0.779	4.999	<0.001	*(PC1 – PC5)*
African colobines	No	1.051 (0.747-1.354)	0.578	0.338	0.738	None
**Mandible**						
**Family**						
Cercopithecidae	No	1.045 (0.860-1.230)	0.874	0.507	0.618	PC2 - PC15; PC4 - PCs 13,16
**Subfamily**						
Cercopithecinae	No	0.951 (0.754-1.148)	0.835	0.523	0.606	PC1 – PC6
**Tribe**						
Papionini	No	0.966 (0.785-1.147)	0.861	0.391	0.7	None
Cercopithecini	No	1.024 (0.780-1.268	0.793	0.204	0.84	*(PC2 - PC5)*
**Subfamily**						
Colobinae	No	0.984 (0.817-1.150)	0.884	0.204	0.841	*(PC1 - PC7)*
**Region**						
Asian colobines	Yes	0.728 (0.572-0.885)	0.825	3.626	0.002	*(PC3 - PCs 4,5)*
African colobines	No	1.152 (0.925-1.80)	0.848	1.398	0.177	None

* Full PC correlation results are provided in Supplementary Table series S3. Italicized comparisons in parentheses are those with p-values below 0.05 that do not meet the Bonferroni criterion.

Table series S2. First 10 principal components for each analysis (42 tables in total).

Table S2.1. First 10 principal components for raw skull data across all Cercopithecidae. Highlighted values depict relatively large loadings (<-0.2 and >0.2).

	PC1	PC2	PC3	PC4	PC5	PC6	PC7	PC8	PC9	PC10
IS-PM	-0.031	-0.028	0.024	-0.055	0.011	0.034	-0.018	-0.033	-0.054	0.007
IS-NSL	-0.164	-0.005	-0.046	0.037	-0.148	-0.018	-0.095	-0.149	**-0.381**	0.113
IS-PNS	**-0.270**	0.036	-0.119	-0.069	-0.108	0.087	-0.014	-0.170	**-0.219**	0.000
PM-ZS	**-0.290**	0.027	**-0.210**	-0.195	-0.146	0.000	0.128	-0.056	-0.116	-0.009
PM-ZI	**-0.220**	0.070	-0.184	-0.092	-0.153	0.047	0.001	-0.078	-0.140	-0.068
PM-MT	-0.154	0.034	0.052	**-0.219**	-0.039	0.186	-0.041	-0.129	**-0.212**	-0.027
NSL-NA	-0.172	-0.044	**-0.207**	**-0.402**	0.050	0.013	**0.362**	**0.232**	**0.296**	0.075
NSL-ZS	-0.145	-0.003	-0.177	**-0.284**	-0.018	0.022	**0.226**	0.006	**0.231**	**-0.222**
NSL-ZI	-0.130	0.001	-0.059	-0.154	0.004	0.055	0.079	-0.013	0.075	-0.137
NA-BR	-0.102	-0.010	**0.403**	0.095	**-0.278**	-0.069	0.080	-0.088	0.038	**-0.291**
NA-FM	-0.060	-0.022	0.049	-0.030	0.023	0.006	-0.035	-0.033	-0.001	-0.063
NA-PNS	-0.134	-0.070	0.053	-0.080	0.060	-0.021	0.017	-0.013	0.012	0.080
BR-ZAF	-0.108	-0.026	**0.339**	0.021	-0.150	-0.015	0.102	-0.092	0.155	**-0.335**
BR-APET	-0.078	0.011	0.163	-0.001	-0.023	0.009	-0.152	**-0.218**	0.128	-0.106
ZAF-FM	-0.045	-0.056	-0.106	0.085	-0.116	0.170	-0.134	-0.072	0.082	-0.195
TS-MT	**-0.229**	0.015	-0.182	**0.287**	-0.069	-0.199	0.016	0.057	0.088	0.020
ZAF-BA	-0.132	0.056	**0.235**	-0.085	0.137	**-0.241**	0.065	-0.058	-0.073	**0.245**
ZAF-EAM	-0.149	0.019	**0.208**	-0.088	0.141	**-0.206**	0.074	-0.013	0.006	0.144
ZAF-ZYGO	-0.105	0.009	0.103	-0.096	0.103	-0.157	-0.005	0.003	0.035	0.179
AS-EAM	-0.047	0.011	0.104	-0.035	0.134	-0.150	0.020	0.121	-0.198	**-0.282**
FM-ZS	-0.038	-0.016	0.037	0.009	-0.001	-0.010	-0.029	0.062	-0.016	0.172
FM-MT	-0.189	-0.046	-0.054	0.081	-0.033	-0.055	-0.003	-0.042	0.044	0.159
ZS-ZI	-0.099	-0.034	0.032	-0.041	0.048	0.013	0.044	0.042	-0.029	0.095
ZI-MT	-0.050	0.007	0.029	-0.071	0.004	0.063	-0.042	-0.101	-0.028	0.108
ZI-ZYGO	-0.124	-0.041	0.041	0.034	0.060	-0.100	-0.055	-0.001	0.093	0.052
NA-ORB	-0.042	-0.028	-0.005	-0.060	0.028	0.008	0.037	0.069	0.040	0.078

MT-PNS	-0.107	0.035	-0.119	0.157	-0.047	-0.054	0.004	-0.006	0.056	-0.008
PNS-APET	-0.098	-0.024	-0.003	0.002	-0.001	-0.073	0.017	0.082	0.031	-0.019
APET-BA	-0.050	0.005	0.043	-0.015	0.021	-0.029	-0.006	-0.023	-0.005	0.037
APET-TS	-0.025	0.014	0.027	0.004	0.009	-0.014	-0.009	-0.011	0.007	0.011
BA-EAM	-0.105	-0.007	0.064	-0.024	0.039	-0.005	-0.050	-0.042	0.061	0.004
EAM-ZYGO	-0.097	0.008	0.070	0.046	0.045	0.019	0.038	0.014	-0.020	0.088
ORB-ZS	-0.012	-0.053	-0.017	0.001	-0.023	-0.008	-0.022	-0.096	0.040	-0.180
LD-AS	-0.083	-0.017	0.092	-0.043	-0.194	**0.226**	-0.109	**-0.286**	**0.442**	0.191
BR-LD	-0.075	0.054	**-0.244**	-0.140	**0.561**	-0.069	**-0.515**	**-0.287**	0.150	**-0.210**
OPI-LD	-0.081	-0.002	0.167	-0.015	-0.140	0.090	-0.072	-0.185	**0.246**	0.091
ZAF-AS	-0.172	0.032	**0.278**	-0.110	**0.245**	**-0.258**	0.095	0.071	-0.106	-0.152
JP-AS	-0.084	0.025	0.113	-0.059	0.072	-0.041	-0.057	-0.009	-0.009	-0.103
BA-OPI	-0.022	-0.015	0.029	-0.036	0.022	0.009	-0.024	0.029	0.028	-0.042
MO-GG	-0.095	0.047	-0.009	-0.065	-0.086	0.066	-0.031	-0.167	**-0.228**	0.009
GG-GH	-0.064	-0.101	-0.007	-0.009	0.003	-0.017	-0.010	-0.047	-0.019	-0.015
GH-IMA	-0.166	**0.529**	0.080	0.030	-0.016	0.088	-0.192	**0.306**	0.107	-0.026
IMA-PMA	-0.132	**-0.678**	0.011	0.037	-0.010	-0.059	-0.145	0.078	-0.002	0.036
PMA-CONL	-0.071	**0.292**	0.080	0.177	0.175	0.200	0.130	-0.090	0.026	0.121
CONL-CONM	-0.053	-0.015	0.024	0.005	0.036	0.030	-0.024	-0.011	0.017	-0.009
CONL-COR	-0.066	-0.026	0.059	-0.041	-0.077	**-0.245**	-0.160	-0.067	0.068	**0.229**
COR-RAMA	-0.175	-0.066	-0.013	**0.295**	**0.302**	**0.331**	**0.271**	-0.018	-0.047	-0.133
RAMA-PMA	-0.161	-0.079	0.006	0.081	0.062	-0.043	0.031	-0.095	0.050	0.052
COR-IMA	-0.184	**-0.229**	0.116	**0.211**	**0.292**	**0.340**	0.106	0.074	-0.040	0.001
MFO-ALV	-0.138	0.043	-0.175	**0.255**	-0.052	**-0.286**	0.045	-0.050	0.084	-0.152
MO-MP3	-0.052	0.053	0.062	-0.080	-0.011	0.101	-0.026	-0.049	-0.126	0.004
MP3-BDM1	-0.080	-0.003	0.029	-0.119	-0.020	0.098	-0.031	-0.006	-0.070	0.023
BDM1-RAMA	-0.089	-0.010	-0.098	0.064	-0.100	-0.092	-0.112	0.157	0.012	-0.074
CON-MALV	**-0.243**	0.068	-0.153	**0.333**	-0.046	-0.172	0.020	0.038	0.118	0.050
MFO-CONM	-0.111	-0.013	0.050	0.077	0.032	0.170	-0.046	0.069	0.015	**0.277**
RAMA-GH	-0.128	-0.006	0.088	-0.109	-0.090	0.082	**-0.298**	**0.367**	0.006	-0.030
MP3-MEN	-0.095	0.048	-0.063	0.001	-0.095	-0.028	-0.027	-0.072	**-0.209**	-0.025

MEN-GH	-0.037	-0.013	0.021	-0.003	0.016	0.034	0.009	-0.068	0.008	0.005
ALV-IMA	-0.114	0.199	0.019	0.097	0.050	0.041	0.032	-0.008	0.016	0.046
ALV-RAMA	-0.023	-0.021	0.030	0.016	0.053	0.070	0.037	-0.063	-0.033	0.028
LDM1-BDM1	-0.120	-0.048	-0.066	0.085	-0.075	0.015	-0.112	0.153	-0.014	-0.067
LDM1-GH	-0.168	-0.066	0.108	-0.038	-0.066	**0.206**	**-0.306**	**0.407**	-0.005	-0.029
Percent variance explained (%)	41.071	6.666	4.428	3.780	3.607	2.955	2.878	2.550	2.288	2.071

Table S2.2. First 10 principal components for log-scale ratio skull data across all Cercopithecidae. Highlighted values depict relatively large loadings (<-0.2 and >0.2).

Trait	PC1	PC2	PC3	PC4	PC5	PC6	PC7	PC8	PC9	PC10
IS-PM	-0.154	0.013	0.026	0.076	0.063	0.114	0.080	0.021	0.015	-0.051
IS-NSL	0.099	0.017	-0.027	-0.029	-0.016	0.158	0.043	0.079	0.040	-0.066
IS-PNS	0.044	-0.019	0.004	-0.027	0.005	0.081	0.061	0.018	0.015	-0.048
PM-ZS	0.146	-0.009	-0.020	-0.027	-0.047	0.119	0.061	0.015	-0.102	-0.078
PM-ZI	0.113	-0.007	0.017	-0.024	-0.001	0.088	0.053	-0.013	-0.049	-0.029
PM-MT	-0.056	-0.055	0.027	0.015	0.034	0.091	0.041	0.010	-0.008	-0.018
NSL-NA	0.034	-0.008	-0.089	0.116	0.012	0.032	0.020	**-0.240**	**-0.557**	-0.132
NSL-ZS	-0.018	0.028	0.191	0.000	-0.124	0.014	0.074	-0.079	**-0.232**	-0.095
NSL-ZI	-0.019	0.001	0.035	0.036	0.004	0.001	0.038	-0.055	-0.060	0.016
NA-BR	-0.064	-0.020	0.034	0.057	0.022	-0.037	-0.031	0.034	0.087	0.028
NA-FM	-0.077	-0.001	0.062	0.056	0.007	-0.029	-0.011	-0.045	-0.020	0.021
NA-PNS	-0.043	0.021	-0.002	0.044	0.012	-0.008	-0.023	-0.056	-0.109	-0.011
BR-ZAF	-0.075	-0.012	0.035	0.046	0.034	-0.044	-0.020	-0.031	0.028	0.024
BR-APET	-0.088	-0.026	0.044	0.046	0.053	-0.042	-0.048	0.019	0.045	0.050
ZAF-FM	-0.019	**0.359**	0.124	**-0.478**	**0.681**	0.006	-0.075	-0.004	-0.079	0.044
TS-MT	**0.260**	0.067	-0.054	-0.081	-0.072	-0.086	-0.012	-0.021	0.042	-0.054
ZAF-BA	-0.042	-0.088	-0.004	0.131	-0.106	-0.033	-0.016	0.034	0.035	-0.022
ZAF-EAM	-0.007	-0.067	-0.024	0.121	-0.097	-0.045	-0.028	0.032	0.027	-0.048
ZAF-ZYGO	-0.015	-0.071	-0.029	0.151	-0.134	-0.054	-0.026	0.063	0.014	-0.008

AS-EAM	-0.074	-0.038	0.039	0.068	0.001	-0.063	-0.008	-0.030	-0.013	0.063
FM-ZS	-0.024	-0.045	-0.172	0.147	**0.200**	-0.039	-0.090	-0.037	0.030	0.049
FM-MT	0.028	0.044	-0.030	-0.015	0.034	-0.018	-0.050	0.009	-0.008	-0.006
ZS-ZI	0.044	-0.038	-0.181	0.052	0.086	0.014	0.043	-0.021	0.063	0.105
ZI-MT	-0.187	-0.030	-0.046	-0.007	0.104	0.057	-0.097	0.116	-0.025	-0.055
ZI-ZYGO	0.006	0.019	-0.023	0.021	-0.018	-0.078	-0.034	0.055	0.043	-0.014
NA-ORB	-0.035	0.004	-0.098	0.128	0.074	0.013	-0.054	**-0.259**	**-0.482**	-0.088
MT-PNS	**0.235**	0.026	-0.015	-0.109	-0.054	-0.101	0.009	-0.052	0.087	-0.011
PNS-APET	0.041	0.042	-0.008	0.025	0.023	-0.028	-0.019	-0.025	-0.032	-0.092
APET-BA	-0.026	-0.019	0.005	0.040	0.020	-0.036	-0.021	0.030	0.010	0.009
APET-TS	-0.071	-0.064	0.010	0.069	-0.009	-0.051	-0.047	-0.045	-0.012	0.101
BA-EAM	-0.033	-0.017	0.013	0.028	0.016	-0.048	-0.025	0.006	0.020	0.019
EAM-ZYGO	0.026	-0.019	-0.050	-0.016	0.013	-0.039	-0.057	-0.032	0.067	-0.084
ORB-ZS	**-0.259**	**0.289**	**0.684**	-0.151	**-0.353**	-0.080	0.006	-0.023	-0.004	-0.058
LD-AS	-0.091	-0.014	0.053	0.049	0.066	-0.013	-0.008	-0.023	0.066	-0.008
BR-LD	-0.093	-0.019	0.053	0.038	0.054	-0.088	-0.083	-0.035	-0.011	0.119
OPI-LD	-0.073	-0.032	0.057	0.055	0.045	-0.020	-0.018	0.015	0.084	-0.038
ZAF-AS	-0.037	-0.055	0.003	0.095	-0.051	-0.052	-0.016	-0.022	0.019	-0.002
JP-AS	-0.052	-0.054	0.038	0.072	0.015	-0.030	-0.028	-0.006	0.029	0.031
BA-OPI	-0.132	-0.011	0.045	0.112	0.035	-0.088	-0.077	-0.068	0.005	0.080
MO-GG	0.057	-0.107	0.083	-0.025	0.082	**0.457**	0.144	0.081	0.124	**-0.347**
GG-GH	-0.043	**0.216**	-0.138	-0.012	-0.152	-0.017	**0.285**	**0.222**	**-0.201**	**0.535**
GH-IMA	0.092	**-0.249**	0.113	-0.037	0.072	-0.089	-0.042	-0.003	0.012	-0.017
IMA-PMA	-0.091	**0.581**	**-0.271**	**0.208**	-0.108	0.113	-0.007	-0.091	**0.259**	-0.145
PMA-CONL	0.044	**-0.372**	0.117	-0.195	0.044	**-0.218**	-0.109	-0.043	0.053	0.183
CONL-CONM	-0.012	-0.007	-0.050	-0.030	-0.014	-0.045	0.023	0.003	0.082	0.055
CONL-COR	-0.013	0.067	-0.042	0.138	0.045	-0.136	**-0.353**	**0.678**	-0.108	-0.161
COR-RAMA	0.040	-0.009	-0.113	-0.195	-0.125	-0.017	0.082	**-0.340**	0.169	0.051
RAMA-PMA	0.019	0.046	-0.065	-0.031	-0.040	-0.013	-0.011	0.049	0.062	-0.088
COR-IMA	-0.012	0.036	-0.065	-0.018	-0.039	-0.022	0.018	-0.161	0.158	0.059

MFO-ALV	**0.433**	0.111	-0.018	-0.182	-0.175	**-0.214**	0.026	0.128	-0.114	**-0.228**
MO-MP3	-0.083	**-0.207**	0.086	0.001	0.059	**0.248**	**0.235**	0.019	0.090	**-0.242**
MP3-BDM1	-0.083	-0.041	0.011	0.038	0.052	0.100	0.014	0.015	-0.011	0.033
BDM1-RAMA	**0.263**	0.135	0.050	0.085	-0.009	-0.066	-0.006	-0.077	0.032	0.092
CON-MALV	**0.235**	0.015	-0.014	-0.071	-0.050	-0.099	-0.023	0.003	0.068	-0.070
MFO-CONM	-0.015	-0.033	-0.036	0.013	0.059	0.042	-0.103	-0.100	**0.246**	0.111
RAMA-GH	0.002	0.000	0.042	0.111	0.067	-0.010	0.006	-0.044	0.043	0.036
MP3-MEN	**0.246**	-0.015	0.125	-0.019	-0.117	**0.600**	-0.149	0.150	-0.088	**0.460**
MEN-GH	-0.135	-0.054	-0.115	-0.089	0.077	-0.195	**0.722**	**0.264**	-0.027	0.015
ALV-IMA	0.137	-0.194	0.025	-0.116	-0.029	-0.029	0.010	0.042	0.031	0.019
ALV-RAMA	**-0.386**	-0.097	**-0.409**	**-0.571**	**-0.345**	0.109	**-0.235**	0.025	-0.089	-0.074
LDM1-BDM1	0.110	0.077	-0.028	0.015	-0.013	-0.032	-0.015	-0.079	0.067	0.045
LDM1-GH	-0.016	0.008	-0.017	0.050	0.029	-0.007	-0.018	-0.075	0.072	0.035
Percent variance explained (%)	9.344	9.127	7.293	6.312	5.905	4.516	4.098	3.622	3.304	3.003

Table S2.3. First 10 principal components for raw skull data across Cercopithecinae. Highlighted values depict relatively large loadings (<-0.2 and >0.2).

	PC1	PC2	PC3	PC4	PC5	PC6	PC7	PC8	PC9	PC10
IS-PM	-0.032	-0.029	0.026	-0.054	0.008	-0.029	0.030	-0.043	-0.054	0.007
IS-NSL	-0.163	-0.007	-0.045	0.055	-0.160	-0.103	-0.038	-0.172	**-0.351**	0.145
IS-PNS	**-0.272**	0.044	-0.121	-0.055	-0.121	-0.043	0.076	-0.193	-0.191	-0.015
PM-ZS	**-0.294**	0.029	**-0.215**	-0.190	-0.151	0.110	0.010	-0.066	-0.111	0.027
PM-ZI	**-0.222**	0.076	-0.184	-0.083	-0.159	-0.021	0.044	-0.092	-0.130	-0.080
PM-MT	-0.156	0.033	0.055	**-0.212**	-0.062	-0.095	0.166	-0.151	**-0.200**	-0.046
NSL-NA	-0.177	-0.045	**-0.213**	**-0.424**	0.074	**0.354**	0.063	**0.254**	**0.255**	0.126
NSL-ZS	-0.149	-0.005	-0.189	**-0.296**	-0.012	**0.210**	0.069	0.000	**0.212**	**-0.245**
NSL-ZI	-0.131	0.003	-0.058	-0.166	0.005	0.060	0.072	-0.020	0.073	-0.165
NA-BR	-0.099	-0.011	**0.396**	0.082	**-0.286**	0.103	-0.076	-0.070	0.059	**-0.249**
NA-FM	-0.059	-0.023	0.050	-0.034	0.020	-0.041	-0.001	-0.032	-0.005	-0.059

NA-PNS	-0.133	-0.073	0.056	-0.085	0.066	0.017	-0.035	-0.003	0.006	0.148
BR-ZAF	-0.106	-0.028	**0.333**	0.002	-0.151	0.112	-0.032	-0.054	0.167	**-0.276**
BR-APET	-0.077	0.009	0.162	-0.003	-0.043	-0.158	-0.028	-0.166	0.149	-0.080
ZAF-FM	-0.046	-0.062	-0.093	0.080	-0.111	-0.143	0.144	-0.019	0.076	-0.147
TS-MT	**-0.227**	0.017	-0.185	**0.294**	-0.049	0.065	-0.179	0.065	0.095	0.007
ZAF-BA	-0.129	0.058	**0.219**	-0.080	0.122	0.079	**-0.232**	-0.102	-0.046	**0.216**
ZAF-EAM	-0.147	0.016	**0.201**	-0.090	0.133	0.088	-0.186	-0.039	0.020	0.117
ZAF-ZYGO	-0.105	0.010	0.103	-0.094	0.089	0.005	-0.141	-0.013	0.033	0.134
AS-EAM	-0.045	0.013	0.108	-0.045	0.131	0.065	-0.164	0.088	**-0.225**	**-0.259**
FM-ZS	-0.037	-0.019	0.041	0.006	0.004	-0.025	-0.020	0.085	-0.014	**0.210**
FM-MT	-0.190	-0.050	-0.057	0.085	-0.026	0.012	-0.067	-0.011	0.054	**0.233**
ZS-ZI	-0.100	-0.034	0.039	-0.050	0.048	0.042	0.003	0.052	-0.028	0.131
ZI-MT	-0.051	0.009	0.024	-0.065	-0.005	-0.064	0.049	-0.093	-0.008	0.136
ZI-ZYGO	-0.123	-0.046	0.044	0.037	0.051	-0.039	-0.092	0.010	0.088	0.029
NA-ORB	-0.042	-0.029	-0.003	-0.059	0.040	0.038	0.015	0.073	0.023	0.107
MT-PNS	-0.107	0.040	-0.123	0.164	-0.039	0.023	-0.048	0.003	0.068	-0.012
PNS-APET	-0.097	-0.031	-0.003	0.000	0.007	0.029	-0.058	0.078	0.020	-0.039
APET-BA	-0.050	0.004	0.045	-0.016	0.019	-0.004	-0.037	-0.024	-0.004	0.046
APET-TS	-0.024	0.015	0.028	0.002	0.008	-0.010	-0.022	-0.009	0.011	0.018
BA-EAM	-0.104	-0.008	0.068	-0.026	0.033	-0.055	-0.014	-0.035	0.065	0.004
EAM-ZYGO	-0.096	0.002	0.070	0.042	0.055	0.031	0.018	0.011	-0.013	0.099
ORB-ZS	-0.011	-0.061	-0.020	0.008	-0.027	-0.026	-0.001	-0.105	0.032	**-0.208**
LD-AS	-0.084	-0.018	0.086	-0.039	**-0.205**	-0.172	**0.233**	**-0.217**	**0.482**	0.129
BR-LD	-0.076	0.049	**-0.233**	-0.123	**0.552**	**-0.536**	-0.146	**-0.237**	0.175	-0.191
OPI-LD	-0.081	-0.005	0.161	-0.008	-0.153	-0.089	0.086	-0.145	**0.262**	0.032
ZAF-AS	-0.168	0.026	**0.273**	-0.124	**0.239**	0.135	**-0.259**	0.028	-0.113	-0.151
JP-AS	-0.083	0.023	0.118	-0.060	0.063	-0.050	-0.054	-0.017	-0.008	-0.105
BA-OPI	-0.021	-0.017	0.030	-0.038	0.025	-0.025	0.012	0.038	0.018	-0.041
MO-GG	-0.095	0.049	-0.011	-0.055	-0.096	-0.057	0.055	-0.187	**-0.208**	0.009
GG-GH	-0.064	-0.101	-0.007	-0.011	-0.005	-0.013	-0.021	-0.058	-0.015	-0.016

GH-IMA	-0.164	**0.525**	0.096	0.029	-0.005	-0.173	0.065	**0.334**	0.066	-0.049
IMA-PMA	-0.131	**-0.676**	0.007	0.052	-0.011	-0.124	-0.067	0.084	-0.014	0.008
PMA-CONL	-0.071	**0.289**	0.095	0.165	0.181	0.092	**0.202**	-0.112	0.028	0.150
CONL-CONM	-0.053	-0.015	0.026	0.002	0.040	-0.030	0.030	-0.008	0.017	-0.010
CONL-COR	-0.065	-0.037	0.052	-0.028	-0.076	-0.135	**-0.273**	-0.035	0.088	**0.241**
COR-RAMA	-0.176	-0.056	0.014	**0.268**	**0.303**	**0.230**	**0.381**	-0.055	-0.059	-0.164
RAMA-PMA	-0.161	-0.087	0.011	0.075	0.054	0.027	-0.028	-0.076	0.059	0.024
COR-IMA	-0.184	**-0.229**	0.140	0.196	**0.297**	0.067	**0.347**	0.034	-0.065	0.001
MFO-ALV	-0.136	0.046	-0.180	**0.254**	-0.040	0.094	**-0.263**	-0.042	0.093	-0.164
MO-MP3	-0.052	0.055	0.067	-0.078	-0.019	-0.049	0.097	-0.073	-0.124	-0.004
MP3-BDM1	-0.082	-0.004	0.029	-0.114	-0.028	-0.054	0.088	-0.010	-0.073	0.018
BDM1-RAMA	-0.088	-0.011	-0.102	0.073	-0.084	-0.080	-0.105	0.159	-0.006	-0.087
CON-MALV	**-0.242**	0.070	-0.151	**0.339**	-0.029	0.062	-0.151	0.048	0.120	0.026
MFO-CONM	-0.112	-0.016	0.060	0.087	0.038	-0.061	0.165	0.064	0.018	**0.273**
RAMA-GH	-0.127	-0.003	0.092	-0.094	-0.077	**-0.274**	0.034	**0.380**	-0.048	-0.034
MP3-MEN	-0.092	0.050	-0.066	0.007	-0.102	-0.030	-0.031	-0.092	**-0.203**	-0.020
MEN-GH	-0.037	-0.010	0.024	-0.007	0.010	-0.004	0.033	-0.066	0.022	-0.002
ALV-IMA	-0.113	**0.207**	0.025	0.096	0.052	0.030	0.035	-0.014	0.027	0.053
ALV-RAMA	-0.023	-0.017	0.034	0.014	0.045	0.022	0.072	-0.066	-0.025	0.033
LDM1-BDM1	-0.120	-0.045	-0.068	0.093	-0.065	-0.100	-0.002	0.169	-0.036	-0.085
LDM1-GH	-0.168	-0.056	0.114	-0.023	-0.055	**-0.295**	0.153	**0.435**	-0.068	-0.043
Percent variance explained (%)	42.592	6.634	4.405	3.822	3.503	2.889	2.833	2.461	2.279	1.983

Table S2.4. First 10 principal components for log-scale ratio skull data across Cercopithecinae. Highlighted values depict relatively large loadings (<-0.2 and >0.2).

	PC1	PC2	PC3	PC4	PC5	PC6	PC7	PC8	PC9	PC10
IS-PM	0.067	-0.149	-0.009	0.066	0.063	-0.120	0.084	-0.003	0.019	0.081
IS-NSL	-0.016	0.103	0.017	-0.031	-0.018	-0.167	0.059	-0.066	0.036	0.066
IS-PNS	-0.035	0.039	-0.015	-0.026	0.005	-0.081	0.069	-0.013	0.012	0.054
PM-ZS	-0.061	0.141	0.010	-0.020	-0.040	-0.124	0.077	-0.006	-0.105	0.092

PM-ZI	-0.048	0.106	-0.029	-0.016	0.001	-0.090	0.060	0.015	-0.051	0.025
PM-MT	-0.032	-0.070	-0.022	0.013	0.033	-0.095	0.041	-0.006	-0.012	0.020
NSL-NA	-0.019	0.028	0.095	0.101	0.012	-0.007	0.039	**0.231**	**-0.522**	0.195
NSL-ZS	0.029	-0.009	-0.179	0.022	-0.112	-0.015	0.089	0.078	**-0.243**	0.120
NSL-ZI	0.008	-0.024	-0.031	0.042	0.005	0.001	0.033	0.056	-0.068	-0.019
NA-BR	0.003	-0.075	-0.021	0.063	0.020	0.033	-0.037	-0.035	0.084	-0.060
NA-FM	0.027	-0.082	-0.053	0.065	0.008	0.033	-0.019	0.040	-0.029	-0.040
NA-PNS	0.035	-0.036	0.009	0.043	0.008	0.009	-0.025	0.056	-0.119	0.026
BR-ZAF	0.015	-0.082	-0.021	0.050	0.031	0.052	-0.030	0.026	0.023	-0.050
BR-APET	0.007	-0.097	-0.031	0.056	0.051	0.041	-0.058	-0.026	0.042	-0.076
ZAF-FM	**0.340**	0.102	-0.179	**-0.474**	**0.673**	-0.005	-0.098	0.016	-0.088	-0.046
TS-MT	-0.025	**0.276**	0.027	-0.077	-0.071	0.083	-0.006	0.021	0.047	0.061
ZAF-BA	-0.065	-0.073	0.022	0.129	-0.101	0.030	-0.015	-0.038	0.034	0.018
ZAF-EAM	-0.056	-0.030	0.040	0.115	-0.088	0.037	-0.021	-0.023	0.026	0.063
ZAF-ZYGO	-0.063	-0.036	0.047	0.150	-0.123	0.028	-0.024	-0.054	0.009	0.009
AS-EAM	-0.014	-0.095	-0.023	0.077	-0.003	0.064	-0.011	0.013	-0.040	-0.141
FM-ZS	-0.030	-0.040	**0.202**	0.124	0.199	0.032	-0.100	0.037	0.031	-0.092
FM-MT	0.032	0.048	0.030	-0.016	0.027	0.013	-0.050	-0.007	-0.013	0.017
ZS-ZI	-0.047	0.030	0.172	0.040	0.073	-0.017	0.021	0.020	0.046	-0.097
ZI-MT	0.034	-0.183	0.064	-0.032	0.099	-0.072	-0.102	-0.109	-0.025	0.104
ZI-ZYGO	0.015	0.016	0.027	0.020	-0.013	0.059	-0.036	-0.050	0.041	0.012
NA-ORB	0.019	-0.040	0.119	0.095	0.060	0.005	-0.049	**0.252**	**-0.490**	0.111
MT-PNS	-0.060	**0.240**	-0.017	-0.096	-0.057	0.112	0.011	0.042	0.087	-0.016
PNS-APET	0.030	0.049	0.018	0.020	0.031	0.024	-0.013	0.040	-0.023	0.111
APET-BA	-0.007	-0.035	0.002	0.042	0.012	0.032	-0.026	-0.043	0.011	-0.018
APET-TS	-0.034	-0.092	-0.006	0.078	-0.017	0.048	-0.055	0.021	-0.017	-0.116
BA-EAM	-0.004	-0.041	-0.008	0.032	0.016	0.050	-0.026	-0.015	0.022	-0.026
EAM-ZYGO	-0.020	0.021	0.054	-0.026	0.005	0.048	-0.049	0.035	0.079	0.127
ORB-ZS	**0.366**	-0.173	**-0.696**	-0.058	**-0.348**	0.112	0.012	0.018	0.014	0.081
LD-AS	0.020	-0.097	-0.038	0.043	0.068	0.021	-0.011	0.028	0.079	-0.013

BR-LD	0.016	-0.093	-0.038	0.059	0.054	0.090	-0.101	0.013	-0.015	-0.128
OPI-LD	-0.001	-0.087	-0.038	0.050	0.048	0.030	-0.015	-0.013	0.087	-0.006
ZAF-AS	-0.036	-0.058	0.015	0.095	-0.050	0.051	-0.016	0.019	0.006	-0.015
JP-AS	-0.031	-0.074	-0.022	0.080	0.019	0.031	-0.027	-0.005	0.026	-0.064
BA-OPI	0.036	-0.137	-0.021	0.118	0.035	0.084	-0.086	0.070	0.004	-0.117
MO-GG	-0.114	0.013	-0.099	-0.012	0.091	**-0.438**	0.177	-0.047	0.146	**0.323**
GG-GH	**0.210**	0.039	0.125	-0.040	-0.152	-0.013	**0.248**	**-0.216**	**-0.229**	**-0.429**
GH-IMA	**-0.261**	-0.004	-0.109	-0.010	0.076	0.086	-0.045	0.003	0.015	-0.003
IMA-PMA	**0.581**	0.117	**0.305**	0.146	-0.120	-0.114	0.018	0.104	**0.266**	0.128
PMA-CONL	**-0.361**	-0.076	-0.137	-0.171	0.040	**0.207**	-0.151	0.029	0.061	-0.078
CONL-CONM	-0.007	-0.016	0.047	-0.039	-0.017	0.051	0.011	0.001	0.085	-0.056
CONL-COR	0.082	0.013	0.074	0.123	0.048	0.118	**-0.288**	**-0.698**	-0.083	**0.274**
COR-RAMA	-0.036	0.055	0.072	-0.194	-0.139	0.013	0.049	**0.346**	0.142	-0.052
RAMA-PMA	0.039	0.041	0.062	-0.037	-0.045	0.004	-0.002	-0.032	0.061	0.105
COR-IMA	0.036	0.007	0.064	-0.029	-0.048	0.019	-0.005	0.159	0.152	-0.035
MFO-ALV	-0.049	**0.451**	-0.034	-0.143	-0.164	**0.214**	0.033	-0.125	-0.107	0.144
MO-MP3	-0.169	-0.155	-0.084	-0.005	0.065	**-0.251**	**0.248**	0.017	0.106	**0.246**
MP3-BDM1	-0.009	-0.090	-0.001	0.031	0.051	-0.104	0.012	-0.010	-0.019	-0.030
BDM1-RAMA	0.043	**0.282**	-0.046	0.107	0.006	0.074	-0.005	0.060	0.037	-0.140
CON-MALV	-0.066	**0.233**	-0.006	-0.062	-0.044	0.096	-0.015	0.001	0.075	0.086
MFO-CONM	-0.020	-0.017	0.042	-0.012	0.053	-0.035	-0.094	0.102	**0.255**	0.004
RAMA-GH	-0.001	-0.009	-0.021	0.110	0.076	0.014	-0.002	0.038	0.049	-0.061
MP3-MEN	-0.086	**0.209**	-0.163	0.025	-0.099	**-0.596**	-0.144	-0.173	-0.107	**-0.465**
MEN-GH	-0.020	-0.148	0.110	-0.133	0.091	**0.236**	**0.738**	**-0.240**	0.006	-0.111
ALV-IMA	**-0.235**	0.076	-0.049	-0.104	-0.028	0.033	-0.002	-0.046	0.034	0.003
ALV-RAMA	0.005	**-0.339**	**0.324**	**-0.632**	**-0.376**	-0.112	**-0.223**	-0.045	-0.083	0.020
LDM1-BDM1	0.034	0.135	0.024	0.020	-0.010	0.033	-0.022	0.069	0.062	-0.075
LDM1-GH	0.008	-0.011	0.026	0.044	0.031	0.009	-0.028	0.068	0.068	-0.052
Percent variance explained (%)	9.683	9.420	7.335	6.274	5.867	4.525	4.238	3.651	3.298	2.933

Table S2.5. First 10 principal components for raw skull data across Colobinae. Highlighted values depict relatively large loadings (<-0.2 and >0.2).

	PC1	PC2	PC3	PC4	PC5	PC6	PC7	PC8	PC9	PC10
IS-PM	-0.018	0.013	-0.010	0.003	-0.011	-0.023	-0.020	0.001	-0.048	0.031
IS-NSL	-0.179	-0.023	-0.004	-0.080	0.054	0.020	-0.052	**-0.215**	-0.171	0.007
IS-PNS	**-0.202**	0.042	0.061	-0.089	-0.026	-0.076	-0.081	-0.131	**-0.220**	0.051
PM-ZS	-0.183	-0.027	-0.007	-0.064	0.100	0.003	-0.050	-0.093	-0.032	-0.020
PM-ZI	-0.163	-0.003	0.022	-0.113	0.140	-0.004	-0.017	-0.124	-0.109	0.055
PM-MT	-0.097	-0.038	0.011	-0.007	-0.119	-0.189	-0.037	-0.058	**-0.228**	0.069
NSL-NA	-0.050	0.028	-0.016	-0.040	0.074	-0.045	0.025	-0.188	0.001	-0.025
NSL-ZS	-0.058	-0.033	-0.090	0.037	0.085	-0.025	-0.052	0.109	**0.243**	0.005
NSL-ZI	-0.120	0.023	0.031	-0.027	0.076	-0.001	0.019	-0.047	-0.022	0.032
NA-BR	-0.173	0.012	-0.185	-0.173	**-0.286**	**0.328**	-0.134	**0.395**	**-0.220**	-0.070
NA-FM	-0.087	0.021	0.020	0.016	-0.018	0.018	-0.011	0.023	0.029	0.026
NA-PNS	-0.132	0.041	0.037	-0.034	-0.054	0.002	-0.034	-0.055	-0.069	0.051
BR-ZAF	-0.152	0.014	-0.062	**-0.281**	**-0.446**	0.092	-0.162	0.123	0.096	**-0.269**
BR-APET	-0.094	-0.029	0.123	-0.034	**-0.286**	0.056	**-0.214**	0.033	0.100	**0.201**
ZAF-FM	-0.012	-0.017	**0.220**	**-0.312**	0.081	0.099	-0.170	0.061	-0.080	**0.204**
TS-MT	**-0.257**	0.007	0.033	-0.081	**0.222**	**0.205**	0.059	-0.059	0.065	-0.134
ZAF-BA	**-0.219**	-0.006	-0.174	**0.355**	**-0.200**	-0.072	0.081	-0.075	0.019	-0.095
ZAF-EAM	-0.194	-0.034	-0.157	**0.360**	-0.081	0.065	0.085	-0.008	-0.087	-0.050
ZAF-ZYGO	-0.103	0.013	-0.099	**0.417**	0.118	0.159	0.071	0.165	-0.155	**0.316**
AS-EAM	-0.102	0.017	0.066	0.061	0.008	-0.082	-0.197	0.024	0.151	-0.179
FM-ZS	-0.055	-0.012	0.042	-0.046	-0.008	0.078	0.034	-0.111	-0.192	0.042
FM-MT	-0.164	-0.012	0.076	-0.082	-0.046	0.091	-0.030	-0.020	-0.116	0.154
ZS-ZI	-0.074	0.034	0.115	-0.037	-0.029	0.008	0.050	-0.084	-0.159	0.000
ZI-MT	-0.030	0.008	0.001	0.036	-0.130	-0.069	-0.027	-0.011	-0.142	0.099
ZI-ZYGO	-0.124	-0.007	0.030	0.195	0.069	0.135	-0.045	**0.228**	-0.068	0.156
NA-ORB	-0.060	0.025	-0.051	-0.043	0.027	-0.056	-0.026	-0.183	0.055	-0.011
MT-PNS	-0.106	0.010	0.033	-0.064	0.056	0.041	-0.010	-0.036	0.055	-0.026

PNS-APET	-0.123	-0.053	-0.073	0.021	0.115	0.041	0.044	-0.025	0.028	-0.102
APET-BA	-0.060	-0.009	0.030	0.009	-0.027	0.007	-0.014	-0.024	0.006	0.046
APET-TS	-0.027	0.010	0.033	-0.019	-0.033	0.009	0.017	0.011	-0.026	-0.026
BA-EAM	-0.108	0.010	0.048	0.010	-0.027	-0.021	-0.048	-0.021	0.092	0.005
EAM-ZYGO	-0.117	-0.073	-0.014	-0.087	-0.114	-0.066	0.120	-0.179	0.019	**-0.210**
ORB-ZS	-0.028	-0.036	-0.045	0.032	0.027	-0.002	-0.056	0.158	**0.201**	0.088
LD-AS	-0.071	0.001	-0.124	-0.095	-0.190	0.066	0.076	-0.099	0.151	**0.415**
BR-LD	-0.047	-0.109	**0.487**	**0.281**	0.031	**-0.209**	**-0.532**	-0.059	0.040	0.031
OPI-LD	-0.091	-0.034	-0.159	-0.021	**-0.221**	-0.079	0.008	-0.177	**0.373**	**0.416**
ZAF-AS	**-0.253**	-0.062	-0.055	**0.251**	-0.173	-0.099	-0.094	0.024	0.081	**-0.332**
JP-AS	-0.093	-0.020	0.008	0.066	-0.012	-0.100	-0.124	-0.022	0.154	-0.044
BA-OPI	-0.025	-0.007	-0.015	0.026	0.003	0.064	-0.058	0.066	-0.079	0.002
MO-GG	-0.096	-0.020	-0.007	-0.088	-0.091	-0.054	-0.074	**-0.217**	-0.061	0.051
GG-GH	-0.047	0.106	0.036	0.033	0.037	0.016	0.026	0.088	-0.023	-0.011
GH-IMA	**-0.208**	**-0.506**	-0.043	-0.080	**0.217**	-0.162	-0.031	**0.252**	-0.049	0.081
IMA-PMA	-0.122	**0.654**	-0.074	0.057	0.067	0.006	-0.076	-0.115	0.078	0.036
PMA-CONL	-0.067	**-0.297**	0.136	-0.066	-0.153	-0.166	**0.395**	0.048	0.124	0.039
CONL-CONM	-0.062	0.027	0.013	-0.019	-0.021	0.012	-0.013	0.002	0.020	0.016
CONL-COR	-0.081	-0.110	-0.169	0.072	0.017	0.125	-0.019	**-0.212**	-0.093	0.107
COR-RAMA	-0.133	0.171	**0.453**	0.032	-0.061	0.024	**0.266**	**0.216**	-0.041	-0.030
RAMA-PMA	-0.148	-0.038	0.157	0.076	-0.043	0.188	0.077	-0.086	-0.043	0.076
COR-IMA	-0.166	**0.240**	**0.261**	-0.026	-0.076	**-0.272**	**0.348**	0.179	0.122	0.056
MFO-ALV	-0.182	-0.011	0.098	-0.025	0.176	**0.327**	-0.031	0.005	**0.282**	-0.006
MO-MP3	-0.044	-0.026	-0.033	-0.006	-0.016	-0.088	-0.022	-0.016	-0.053	0.022
MP3-BDM1	-0.039	-0.009	-0.028	0.011	-0.049	-0.103	-0.017	0.003	-0.142	0.049
BDM1-RAMA	-0.121	0.019	-0.106	-0.119	0.197	-0.045	0.006	0.033	0.118	-0.063
CON-MALV	**-0.257**	-0.059	0.058	-0.028	0.198	**0.218**	0.130	-0.040	0.119	-0.067
MFO-CONM	-0.087	-0.014	-0.057	-0.031	0.020	-0.181	0.163	-0.101	**-0.280**	-0.088
RAMA-GH	-0.155	0.080	**-0.249**	-0.116	**0.216**	**-0.312**	-0.128	0.180	-0.002	0.073
MP3-MEN	-0.141	-0.010	0.015	-0.064	0.039	-0.019	-0.028	-0.194	0.054	-0.046

MEN-GH	-0.034	0.044	0.067	0.013	-0.038	0.026	0.043	0.004	-0.065	0.046
ALV-IMA	-0.125	-0.080	0.050	-0.037	-0.032	-0.136	0.125	-0.011	0.087	0.063
ALV-RAMA	-0.020	0.067	0.101	0.042	-0.080	0.004	0.019	0.014	-0.112	0.049
LDM1-BDM1	-0.115	0.080	-0.028	-0.096	0.065	-0.032	0.026	0.082	0.040	-0.027
LDM1-GH	-0.160	**0.207**	-0.175	-0.114	0.092	**-0.326**	-0.084	**0.268**	-0.080	0.096
Percent variance explained (%)	23.644	7.988	5.746	5.497	4.822	4.317	3.616	3.401	3.150	2.814

Table S2.6. First 10 principal components for log-scale ratio skull data across Colobinae. Highlighted values depict relatively large loadings (<-0.2 and >0.2).

	PC1	PC2	PC3	PC4	PC5	PC6	PC7	PC8	PC9	PC10
IS-PM	0.064	0.068	0.037	-0.046	0.096	-0.043	0.001	-0.069	0.078	0.099
IS-NSL	-0.063	-0.047	0.015	-0.017	-0.058	-0.012	-0.036	-0.133	-0.152	0.063
IS-PNS	-0.001	-0.027	0.029	-0.022	0.011	-0.027	0.026	-0.063	-0.019	0.015
PM-ZS	-0.075	-0.038	-0.062	0.011	-0.095	-0.053	-0.012	0.019	-0.046	0.038
PM-ZI	-0.065	-0.050	0.007	0.006	-0.010	-0.055	-0.009	0.021	-0.042	0.019
PM-MT	0.041	0.075	0.071	-0.053	0.029	-0.025	0.060	-0.044	0.026	0.045
NSL-NA	-0.024	-0.127	-0.048	**-0.291**	0.033	**-0.266**	-0.121	**0.564**	**0.433**	-0.040
NSL-ZS	-0.057	**0.255**	-0.125	0.147	-0.026	-0.146	0.010	0.050	0.034	-0.088
NSL-ZI	-0.012	0.006	0.006	0.010	0.044	-0.001	0.019	-0.005	0.006	-0.029
NA-BR	0.012	0.045	0.034	0.039	0.052	0.026	-0.033	-0.058	-0.033	0.031
NA-FM	0.017	0.055	0.001	0.010	0.045	0.006	0.014	-0.048	0.016	-0.051
NA-PNS	0.022	0.004	0.024	-0.009	0.043	-0.012	-0.015	0.025	0.035	-0.008
BR-ZAF	0.012	0.040	0.058	-0.007	0.028	-0.053	0.071	-0.029	0.013	-0.032
BR-APET	0.050	0.054	0.080	0.031	0.022	0.013	0.023	-0.057	0.080	0.005
ZAF-FM	0.071	**-0.234**	**0.589**	**0.479**	0.074	**-0.264**	-0.153	0.090	-0.074	0.172
TS-MT	-0.145	-0.165	-0.077	0.056	-0.057	0.096	-0.079	0.058	-0.129	-0.081
ZAF-BA	-0.006	0.089	-0.109	-0.103	0.011	0.108	0.005	-0.088	0.052	-0.055
ZAF-EAM	-0.020	0.088	-0.137	-0.091	-0.014	0.176	-0.074	-0.074	0.024	-0.007
ZAF-ZYGO	0.031	0.118	**-0.207**	-0.001	0.072	**0.256**	**-0.206**	-0.074	0.094	0.052
AS-EAM	-0.006	0.039	0.022	0.063	0.043	-0.012	-0.027	-0.096	0.026	-0.119

FM-ZS	0.016	-0.082	0.148	-0.105	0.078	0.093	-0.102	-0.010	0.076	-0.014
FM-MT	0.015	-0.017	0.066	0.039	0.004	0.012	-0.054	-0.016	0.012	0.036
ZS-ZI	0.049	**-0.285**	0.131	-0.131	0.083	0.183	0.129	-0.076	0.044	0.032
ZI-MT	0.135	0.111	0.088	-0.028	0.042	-0.021	-0.050	-0.034	0.077	0.151
ZI-ZYGO	0.021	0.033	-0.064	0.095	0.037	0.153	-0.116	-0.040	0.040	0.015
NA-ORB	-0.069	-0.021	0.049	**-0.208**	0.148	-0.170	-0.165	0.156	0.087	-0.166
MT-PNS	-0.102	-0.140	-0.011	0.070	-0.050	0.007	-0.008	-0.019	-0.111	-0.147
PNS-APET	-0.084	0.027	-0.044	0.012	-0.029	0.022	-0.092	0.075	-0.114	0.032
APET-BA	-0.003	-0.003	0.071	0.010	0.039	0.032	-0.029	-0.056	0.141	-0.082
APET-TS	0.063	-0.032	0.063	-0.051	0.049	0.091	0.098	0.094	0.132	-0.185
BA-EAM	0.011	-0.001	0.015	0.013	0.008	0.007	-0.001	-0.007	0.033	-0.089
EAM-ZYGO	-0.039	-0.004	0.078	-0.094	-0.106	0.008	0.039	0.044	-0.097	-0.081
ORB-ZS	-0.005	**0.467**	**-0.232**	**0.424**	-0.171	**-0.306**	0.154	0.107	0.066	0.084
LD-AS	-0.001	0.092	0.047	-0.054	0.046	-0.053	-0.009	-0.064	0.076	-0.039
BR-LD	0.090	0.068	0.096	0.076	-0.009	0.036	0.084	-0.032	0.155	-0.040
OPI-LD	-0.037	0.123	0.037	-0.078	-0.010	-0.069	-0.010	-0.149	0.095	-0.078
ZAF-AS	-0.020	0.074	-0.037	-0.042	0.002	0.082	0.012	-0.083	0.012	-0.083
JP-AS	-0.003	0.054	0.010	-0.008	-0.014	-0.015	-0.008	-0.046	0.063	-0.119
BA-OPI	0.054	0.102	0.019	0.017	0.077	0.057	-0.116	0.051	0.005	0.009
MO-GG	-0.063	-0.051	0.104	**-0.229**	**-0.215**	**-0.270**	0.097	**-0.311**	0.084	0.179
GG-GH	0.111	-0.144	**-0.265**	0.123	**0.319**	0.188	**0.292**	**0.269**	-0.045	**0.460**
GH-IMA	-0.135	0.116	0.161	0.041	-0.200	0.165	-0.029	0.048	-0.092	-0.029
IMA-PMA	0.075	-0.136	**-0.269**	-0.010	**0.372**	**-0.287**	-0.135	**-0.219**	-0.077	-0.085
PMA-CONL	-0.019	0.152	**0.226**	-0.025	**-0.238**	**0.305**	**0.292**	**0.323**	0.068	-0.086
CONL-CONM	0.005	-0.065	-0.031	0.019	-0.007	-0.016	0.024	-0.054	0.032	0.027
CONL-COR	-0.046	0.147	0.027	-0.131	-0.108	0.132	**-0.499**	0.077	-0.162	**0.359**
COR-RAMA	0.168	-0.172	-0.035	0.150	-0.019	0.090	**0.220**	-0.147	-0.021	**-0.300**
RAMA-PMA	0.025	-0.033	0.005	0.032	-0.090	0.102	-0.070	-0.114	0.055	0.067
COR-IMA	0.040	-0.029	-0.009	0.028	0.091	0.015	0.168	-0.043	0.010	-0.153
MFO-ALV	**-0.220**	**-0.319**	**-0.215**	**0.311**	**-0.234**	0.135	**-0.226**	-0.049	**0.272**	-0.006

MO-MP3	-0.001	0.133	0.068	-0.124	0.004	-0.053	0.041	-0.149	0.021	0.124
MP3-BDM1	0.054	0.091	0.067	-0.069	0.061	-0.022	0.052	-0.058	0.019	0.096
BDM1-RAMA	**-0.275**	-0.096	-0.131	0.073	0.062	-0.086	0.030	**0.229**	**-0.253**	-0.151
CON-MALV	-0.144	-0.093	-0.064	0.073	-0.086	0.105	-0.089	0.025	-0.113	-0.051
MFO-CONM	-0.001	0.100	0.084	**-0.228**	0.036	-0.012	0.082	0.131	**-0.540**	-0.021
RAMA-GH	-0.087	0.064	-0.030	-0.011	0.163	-0.077	0.010	0.083	-0.110	-0.023
MP3-MEN	**-0.252**	**-0.249**	-0.161	-0.184	**-0.332**	**-0.241**	**0.298**	-0.085	-0.004	**0.288**
MEN-GH	0.137	-0.069	-0.035	0.052	0.154	0.132	0.176	0.009	0.075	**0.276**
ALV-IMA	-0.039	0.010	0.039	-0.038	-0.104	0.052	0.117	0.003	0.014	0.029
ALV-RAMA	**0.754**	-0.156	-0.199	-0.049	**-0.418**	-0.115	-0.139	0.124	-0.176	-0.084
LDM1-BDM1	-0.044	-0.054	-0.046	0.039	0.064	-0.033	0.043	0.022	-0.134	-0.122
LDM1-GH	0.021	0.040	-0.027	-0.012	0.153	-0.069	0.025	0.003	-0.140	-0.061
Percent variance explained (%)	10.612	8.045	6.638	6.435	5.356	5.130	4.240	3.969	3.548	3.332

Table S2.7. First 10 principal components for raw skull data across Papionini. Highlighted values depict relatively large loadings (<-0.2 and >0.2).

	PC1	PC2	PC3	PC4	PC5	PC6	PC7	PC8	PC9	PC10
IS-PM	-0.034	-0.031	0.028	-0.045	0.007	-0.031	0.012	-0.070	0.029	-0.003
IS-NSL	-0.158	0.006	-0.014	0.104	-0.154	-0.112	-0.018	**-0.282**	**0.272**	0.149
IS-PNS	**-0.280**	0.050	-0.107	0.008	-0.112	-0.081	0.090	**-0.248**	0.096	-0.095
PM-ZS	**-0.307**	0.051	**-0.201**	-0.148	-0.174	0.085	0.044	-0.108	0.113	0.075
PM-ZI	**-0.227**	0.116	-0.158	-0.042	-0.153	-0.046	0.098	-0.158	0.076	-0.107
PM-MT	-0.156	0.003	0.077	-0.187	-0.070	-0.141	0.172	**-0.254**	0.090	-0.114
NSL-NA	-0.197	-0.032	**-0.253**	**-0.463**	0.000	**0.316**	0.064	**0.295**	-0.101	0.170
NSL-ZS	-0.168	0.002	**-0.213**	**-0.295**	-0.028	0.190	0.090	0.091	-0.197	**-0.205**
NSL-ZI	-0.133	0.021	-0.064	-0.178	0.007	0.044	0.084	-0.005	-0.112	-0.185
NA-BR	-0.087	-0.035	**0.399**	0.105	**-0.257**	0.189	-0.048	0.002	-0.094	-0.145
NA-FM	-0.055	-0.035	0.060	-0.025	0.032	-0.033	-0.028	-0.036	-0.018	-0.021
NA-PNS	-0.130	-0.079	0.058	-0.086	0.064	0.035	-0.096	-0.011	0.034	**0.292**
BR-ZAF	-0.102	-0.043	**0.337**	0.002	-0.147	0.191	-0.062	0.049	-0.167	-0.150

BR-APET	-0.071	0.002	0.172	0.019	-0.046	-0.068	-0.071	-0.092	-0.190	0.014
ZAF-FM	-0.046	-0.042	-0.053	0.090	-0.057	-0.110	0.118	0.025	-0.104	-0.008
TS-MT	**-0.226**	0.072	-0.179	**0.307**	-0.023	0.083	-0.138	0.118	-0.038	0.006
ZAF-BA	-0.118	0.038	0.188	-0.089	0.065	0.089	**-0.215**	-0.137	0.024	0.090
ZAF-EAM	-0.141	-0.003	0.172	-0.106	0.071	0.099	-0.168	-0.054	-0.029	0.029
ZAF-ZYGO	-0.104	-0.003	0.077	-0.085	0.038	-0.006	-0.119	-0.019	-0.032	0.040
AS-EAM	-0.039	-0.003	0.092	-0.065	0.134	0.089	**-0.205**	-0.003	**0.245**	**-0.203**
FM-ZS	-0.036	-0.013	0.036	-0.013	-0.003	-0.012	-0.014	0.064	0.041	**0.203**
FM-MT	-0.195	-0.008	-0.048	0.113	-0.014	0.044	-0.070	0.042	-0.003	**0.364**
ZS-ZI	-0.104	-0.040	0.037	-0.071	0.035	0.049	-0.040	0.031	0.065	0.166
ZI-MT	-0.055	0.030	0.038	-0.058	-0.012	-0.075	0.055	-0.088	-0.027	0.174
ZI-ZYGO	-0.118	-0.061	0.027	0.041	0.048	-0.031	-0.106	0.025	-0.083	-0.024
NA-ORB	-0.039	-0.027	0.005	-0.070	0.035	0.028	0.013	0.059	0.020	0.148
MT-PNS	-0.110	0.071	-0.124	0.196	-0.017	0.036	-0.031	0.059	-0.044	-0.010
PNS-APET	-0.091	-0.022	-0.003	-0.021	-0.001	0.037	-0.042	0.046	0.003	-0.072
APET-BA	-0.045	0.004	0.045	-0.022	0.012	0.009	-0.042	-0.025	-0.005	0.052
APET-TS	-0.022	0.017	0.026	0.003	0.010	-0.003	-0.024	-0.004	-0.014	0.039
BA-EAM	-0.101	-0.024	0.071	-0.032	0.033	-0.042	-0.036	-0.023	-0.087	0.009
EAM-ZYGO	-0.092	0.001	0.080	0.014	0.036	0.030	0.027	0.011	0.014	0.080
ORB-ZS	-0.012	-0.071	-0.018	0.056	-0.006	-0.026	-0.021	-0.068	-0.068	-0.163
LD-AS	-0.091	-0.028	0.087	0.015	-0.196	-0.173	**0.238**	-0.042	**-0.559**	0.062
BR-LD	-0.077	0.076	**-0.210**	-0.123	**0.532**	**-0.447**	**-0.255**	-0.178	**-0.295**	-0.072
OPI-LD	-0.081	-0.031	0.143	0.045	-0.130	-0.072	0.059	-0.051	**-0.276**	0.004
ZAF-AS	-0.159	0.003	**0.234**	-0.160	0.186	0.153	**-0.278**	-0.039	0.102	-0.176
JP-AS	-0.082	0.003	0.115	-0.056	0.065	-0.038	-0.085	-0.039	-0.013	-0.077
BA-OPI	-0.021	-0.020	0.029	-0.046	0.021	-0.023	0.005	0.034	-0.016	-0.039
MO-GG	-0.097	0.054	0.015	-0.016	-0.094	-0.068	0.075	**-0.240**	0.120	-0.017
GG-GH	-0.065	-0.095	-0.008	0.001	-0.003	-0.026	-0.037	-0.072	-0.026	-0.017
GH-IMA	-0.154	**0.495**	0.179	-0.025	0.026	**-0.216**	0.028	**0.335**	0.034	-0.117
IMA-PMA	-0.134	**-0.664**	-0.079	0.082	-0.035	-0.119	-0.113	0.060	0.017	-0.028

PMA-CONL	-0.066	**0.265**	0.158	0.114	**0.234**	0.094	0.184	-0.056	-0.023	**0.252**
CONL-CONM	-0.054	-0.023	0.031	-0.005	0.044	-0.036	0.006	-0.006	-0.028	-0.009
CONL-COR	-0.059	-0.027	0.046	-0.012	-0.113	-0.102	**-0.292**	-0.027	-0.097	**0.257**
COR-RAMA	-0.178	-0.073	0.049	**0.204**	**0.382**	0.196	**0.398**	-0.021	0.055	-0.193
RAMA-PMA	-0.161	-0.091	0.002	0.075	0.053	0.025	-0.021	-0.049	-0.080	-0.042
COR-IMA	-0.182	**-0.282**	0.145	0.121	**0.343**	0.038	**0.266**	0.062	0.080	0.046
MFO-ALV	-0.133	0.084	-0.178	**0.276**	-0.016	0.136	**-0.233**	-0.004	-0.092	-0.156
MO-MP3	-0.052	0.021	0.086	-0.068	-0.020	-0.076	0.082	-0.119	0.070	-0.036
MP3-BDM1	-0.083	-0.018	0.026	-0.103	-0.031	-0.069	0.089	-0.059	0.041	0.001
BDM1-RAMA	-0.086	0.017	-0.097	0.076	-0.084	-0.082	-0.114	0.139	0.056	-0.095
CON-MALV	**-0.236**	0.100	-0.135	**0.339**	0.005	0.095	-0.105	0.111	-0.067	0.008
MFO-CONM	-0.108	-0.011	0.075	0.058	0.061	-0.072	0.163	0.099	0.022	**0.247**
RAMA-GH	-0.122	-0.031	0.108	-0.102	-0.084	**-0.308**	-0.049	**0.318**	0.142	-0.031
MP3-MEN	-0.092	0.062	-0.042	0.032	-0.099	-0.025	-0.013	-0.125	0.170	-0.027
MEN-GH	-0.038	-0.029	0.021	0.005	0.029	0.010	0.018	-0.086	-0.087	-0.003
ALV-IMA	-0.105	0.190	0.051	0.051	0.073	0.024	0.018	0.018	-0.030	0.078
ALV-RAMA	-0.023	-0.025	0.040	0.009	0.059	0.014	0.075	-0.069	-0.001	0.043
LDM1-BDM1	-0.118	-0.031	-0.065	0.099	-0.060	-0.120	0.010	0.141	0.086	-0.117
LDM1-GH	-0.161	-0.097	0.130	-0.037	-0.055	**-0.350**	0.099	**0.375**	0.178	-0.062
Percent variance explained (%)	44.999	6.089	4.431	4.002	3.427	2.860	2.688	2.432	2.296	1.903

Table S2.8. First 10 principal components for log-scale ratio skull data across Papionini. Highlighted values depict relatively large loadings (<-0.2 and >0.2).

	PC1	PC2	PC3	PC4	PC5	PC6	PC7	PC8	PC9	PC10
IS-PM	-0.141	-0.033	-0.029	-0.055	-0.020	0.042	-0.178	0.075	0.016	0.087
IS-NSL	0.103	0.015	0.027	0.003	0.048	0.078	-0.140	0.104	-0.042	-0.034
IS-PNS	0.060	0.003	0.013	0.023	0.019	0.011	-0.104	0.035	-0.015	-0.008
PM-ZS	0.165	-0.011	0.021	-0.011	0.061	0.035	-0.122	0.034	0.088	-0.038
PM-ZI	0.125	0.012	-0.017	0.027	0.010	0.016	-0.124	0.013	0.044	-0.029
PM-MT	-0.049	-0.046	-0.038	0.004	-0.024	0.040	-0.118	0.031	-0.007	0.022

NSL-NA	0.069	-0.068	0.007	-0.127	0.013	0.047	-0.080	-0.199	**0.476**	-0.135
NSL-ZS	0.014	0.088	-0.079	0.004	0.060	-0.033	-0.137	-0.063	**0.270**	-0.061
NSL-ZI	-0.017	-0.004	-0.045	-0.018	-0.023	-0.014	-0.055	-0.042	0.082	0.016
NA-BR	-0.068	-0.027	-0.073	-0.020	-0.036	-0.020	0.064	0.024	-0.111	0.016
NA-FM	-0.089	0.006	-0.076	-0.038	-0.031	-0.025	0.040	-0.056	0.006	0.041
NA-PNS	-0.042	-0.006	-0.018	-0.056	-0.005	0.025	0.026	-0.066	0.134	-0.029
BR-ZAF	-0.071	-0.015	-0.052	-0.022	-0.060	-0.023	0.054	-0.036	-0.042	0.008
BR-APET	-0.083	-0.030	-0.068	-0.017	-0.061	-0.020	0.091	0.021	-0.056	0.019
ZAF-FM	-0.071	**0.303**	**0.478**	**0.263**	**-0.664**	0.193	-0.010	-0.024	0.071	0.117
TS-MT	**0.257**	0.093	0.093	0.047	0.072	-0.067	0.056	-0.025	-0.037	-0.079
ZAF-BA	-0.041	-0.076	-0.131	-0.060	0.082	-0.053	0.042	0.031	0.000	-0.006
ZAF-EAM	-0.009	-0.051	-0.076	-0.050	0.045	-0.025	0.020	0.007	0.023	-0.016
ZAF-ZYGO	0.002	-0.059	-0.074	-0.068	0.065	-0.015	0.052	0.028	0.012	-0.007
AS-EAM	-0.085	-0.042	-0.089	-0.033	-0.007	-0.048	0.091	-0.041	0.074	**0.593**
FM-ZS	-0.010	-0.167	0.054	-0.122	-0.169	0.050	0.106	-0.088	-0.022	0.003
FM-MT	0.048	0.022	0.051	0.011	-0.022	0.021	0.039	-0.003	0.040	-0.056
ZS-ZI	0.072	-0.142	0.062	-0.102	0.004	0.021	0.019	0.015	-0.045	0.090
ZI-MT	-0.151	-0.133	0.031	0.045	-0.095	0.088	-0.023	0.116	0.088	-0.078
ZI-ZYGO	0.005	0.009	0.034	-0.040	0.014	-0.037	0.102	0.026	-0.075	0.032
NA-ORB	-0.047	-0.094	0.012	-0.126	-0.051	0.105	0.005	**-0.329**	**0.460**	**-0.202**
MT-PNS	**0.277**	0.104	0.070	0.108	0.058	-0.105	0.100	-0.071	-0.100	-0.092
PNS-APET	0.016	0.015	0.028	-0.044	-0.039	-0.009	-0.053	-0.002	0.057	0.048
APET-BA	-0.040	-0.035	-0.057	-0.018	-0.012	-0.026	0.078	0.024	0.018	0.011
APET-TS	-0.058	-0.066	-0.105	-0.016	-0.002	-0.034	0.118	-0.076	0.015	-0.038
BA-EAM	-0.039	-0.018	-0.034	-0.019	-0.024	-0.029	0.064	-0.004	-0.031	0.021
EAM-ZYGO	0.006	-0.033	0.021	0.022	-0.006	0.027	0.011	-0.035	-0.033	-0.091
ORB-ZS	**-0.393**	**0.724**	**-0.296**	0.184	0.166	-0.114	-0.010	-0.023	0.054	-0.089
LD-AS	-0.073	0.000	-0.021	-0.021	-0.082	-0.003	-0.012	-0.036	-0.142	**-0.468**
BR-LD	-0.087	-0.021	-0.058	-0.019	-0.054	-0.056	0.161	-0.061	0.017	**0.202**
OPI-LD	-0.061	-0.011	-0.032	-0.019	-0.058	-0.020	0.035	0.012	-0.114	-0.166

ZAF-AS	-0.039	-0.047	-0.078	-0.041	0.016	-0.032	0.039	-0.036	0.026	0.147
JP-AS	-0.050	-0.041	-0.078	-0.027	-0.039	-0.020	0.056	-0.008	-0.053	0.141
BA-OPI	-0.136	-0.038	-0.098	-0.074	-0.039	-0.008	0.105	-0.106	-0.050	0.062
MO-GG	0.072	-0.048	-0.082	0.084	-0.061	0.111	**-0.417**	0.153	-0.123	0.037
GG-GH	-0.047	0.082	0.176	-0.152	0.160	-0.098	-0.172	0.198	0.138	0.085
GH-IMA	0.075	-0.106	-0.166	0.168	-0.116	-0.067	0.068	-0.019	-0.021	-0.032
IMA-PMA	-0.089	**0.210**	**0.314**	**-0.486**	0.182	0.174	0.005	-0.084	**-0.261**	0.024
PMA-CONL	0.036	-0.160	-0.167	**0.418**	-0.081	-0.129	0.118	-0.036	-0.054	-0.007
CONL-CONM	-0.013	-0.021	0.052	0.008	0.006	-0.014	0.035	0.010	-0.086	0.046
CONL-COR	-0.039	-0.006	0.018	-0.153	-0.092	-0.003	**0.387**	**0.671**	0.136	-0.176
COR-RAMA	0.053	-0.008	0.111	0.150	0.188	0.029	-0.063	**-0.305**	-0.147	0.055
RAMA-PMA	0.019	0.019	0.100	-0.013	0.068	0.017	0.012	0.022	-0.034	-0.016
COR-IMA	-0.021	0.000	0.053	-0.030	0.069	0.024	0.000	-0.140	-0.158	0.043
MFO-ALV	**0.411**	0.198	0.127	0.093	0.116	**-0.225**	0.157	0.042	0.160	0.163
MO-MP3	-0.082	-0.109	-0.096	0.074	-0.059	0.009	**-0.395**	0.073	-0.123	0.080
MP3-BDM1	-0.070	-0.055	-0.020	-0.009	-0.031	0.074	-0.084	0.030	0.017	-0.009
BDM1-RAMA	**0.222**	0.136	-0.051	-0.154	-0.046	-0.060	0.041	-0.046	-0.079	0.037
CON-MALV	**0.218**	0.067	0.043	0.062	0.040	-0.079	0.052	-0.021	-0.065	-0.076
MFO-CONM	-0.009	-0.046	-0.008	0.042	-0.020	0.077	-0.005	-0.074	**-0.265**	**-0.262**
RAMA-GH	-0.017	-0.009	-0.076	-0.107	-0.089	0.009	0.022	-0.027	-0.095	0.022
MP3-MEN	**0.250**	0.123	**-0.238**	0.056	0.117	**0.525**	-0.132	**0.258**	0.070	0.098
MEN-GH	-0.153	-0.112	**0.294**	0.005	0.051	**-0.608**	**-0.317**	0.169	0.042	-0.039
ALV-IMA	0.118	-0.072	-0.066	0.170	0.001	-0.069	0.013	0.035	0.003	-0.037
ALV-RAMA	**-0.288**	-0.196	**0.380**	**0.420**	**0.497**	**0.282**	0.191	0.053	0.071	0.011
LDM1-BDM1	0.103	0.052	0.031	-0.064	0.012	0.010	0.037	-0.065	-0.105	0.006
LDM1-GH	-0.020	-0.019	-0.009	-0.060	-0.023	0.049	0.039	-0.067	-0.119	-0.009
Percent variance explained (%)	10.387	9.596	7.027	6.309	6.049	5.153	4.449	3.833	3.117	2.940

Table S2.9. First 10 principal components for raw skull data across Cercopithecini. Highlighted values depict relatively large loadings (<-0.2 and >0.2).

	PC1	PC2	PC3	PC4	PC5	PC6	PC7	PC8	PC9	PC10
IS-PM	-0.023	-0.031	0.009	0.048	0.034	-0.050	-0.037	-0.002	0.051	-0.044
IS-NSL	-0.180	-0.040	-0.143	0.103	0.036	0.115	-0.126	-0.059	-0.072	-0.198
IS-PNS	**-0.234**	0.053	-0.098	0.148	0.040	-0.006	**-0.234**	-0.089	0.032	-0.097
PM-ZS	**-0.231**	0.019	-0.153	0.146	0.069	0.178	-0.195	-0.031	0.020	-0.044
PM-ZI	-0.194	0.018	-0.172	0.135	0.044	0.117	-0.066	-0.029	0.051	-0.098
PM-MT	-0.148	0.068	-0.015	0.183	0.090	-0.027	-0.155	-0.019	0.114	-0.137
NSL-NA	-0.093	-0.027	0.004	-0.036	0.030	0.081	-0.133	-0.105	**0.329**	-0.031
NSL-ZS	-0.069	0.020	-0.035	0.144	0.065	0.114	**-0.281**	0.072	-0.003	0.153
NSL-ZI	-0.119	-0.033	-0.032	0.088	0.016	0.011	-0.120	0.055	0.072	-0.077
NA-BR	-0.145	-0.024	0.185	**0.320**	**-0.216**	0.042	0.092	**0.291**	**-0.290**	0.067
NA-FM	-0.070	-0.014	-0.019	0.080	0.044	0.009	-0.069	0.036	0.020	0.023
NA-PNS	-0.143	-0.075	0.003	0.078	0.048	-0.042	-0.127	-0.036	0.078	0.026
BR-ZAF	-0.117	-0.048	0.133	**0.234**	-0.140	-0.199	0.022	**0.412**	**-0.247**	-0.034
BR-APET	-0.100	-0.007	0.045	0.178	0.144	**-0.264**	0.104	0.032	-0.169	0.100
ZAF-FM	-0.044	-0.096	**-0.303**	0.138	-0.004	-0.113	0.069	0.016	-0.065	0.101
TS-MT	**-0.227**	-0.063	-0.133	-0.162	-0.102	0.158	0.117	-0.001	-0.164	0.058
ZAF-BA	-0.171	0.058	**0.375**	-0.091	0.073	0.076	-0.049	-0.124	-0.040	-0.154
ZAF-EAM	-0.171	0.017	**0.363**	-0.139	0.073	0.081	0.013	-0.043	-0.022	-0.150
ZAF-ZYGO	-0.111	0.026	**0.312**	-0.080	0.118	0.152	-0.020	**-0.256**	0.047	**0.216**
AS-EAM	-0.069	0.024	0.112	0.030	0.033	0.088	-0.034	**0.251**	0.157	**0.218**
FM-ZS	-0.045	-0.039	0.042	-0.038	-0.020	-0.006	0.187	-0.117	0.065	-0.103
FM-MT	-0.164	-0.119	-0.023	0.001	0.025	0.000	0.028	-0.114	-0.067	-0.017
ZS-ZI	-0.084	-0.032	0.018	-0.029	-0.024	-0.079	0.045	-0.005	0.084	-0.119
ZI-MT	-0.034	-0.040	0.032	0.043	0.061	-0.054	-0.055	-0.071	-0.053	-0.014
ZI-ZYGO	-0.140	-0.023	0.081	-0.022	0.028	0.080	0.022	-0.178	-0.042	**0.463**
NA-ORB	-0.050	-0.042	-0.030	-0.006	0.022	0.044	-0.033	-0.030	0.094	-0.019
MT-PNS	-0.092	0.008	-0.058	-0.051	-0.036	0.013	0.035	-0.015	-0.052	0.000
PNS-APET	-0.116	-0.065	-0.034	-0.052	-0.006	0.083	0.141	0.055	-0.009	-0.074
APET-BA	-0.069	-0.012	0.024	0.009	0.023	-0.005	-0.018	-0.026	-0.008	-0.029

APET-TS	-0.034	0.006	0.031	0.022	0.001	-0.004	0.001	0.011	0.000	0.020
BA-EAM	-0.117	0.005	0.023	0.023	0.031	-0.035	0.036	-0.034	-0.028	0.019
EAM-ZYGO	-0.111	-0.024	0.019	-0.150	-0.017	-0.091	0.049	0.153	-0.055	**-0.536**
ORB-ZS	-0.007	-0.027	-0.030	0.120	0.053	0.031	**-0.213**	0.060	-0.048	0.111
LD-AS	-0.053	0.007	0.062	**0.217**	-0.052	**-0.336**	0.097	**-0.308**	-0.134	-0.058
BR-LD	-0.070	-0.009	-0.128	-0.169	**0.836**	**-0.241**	0.119	0.164	-0.152	0.076
OPI-LD	-0.077	0.045	0.167	**0.258**	-0.095	**-0.269**	0.063	**-0.274**	-0.001	0.146
ZAF-AS	**-0.204**	0.022	**0.410**	-0.106	0.066	0.080	-0.044	**0.293**	0.114	-0.030
JP-AS	-0.088	0.043	0.123	0.069	0.054	-0.009	0.036	0.077	0.070	0.144
BA-OPI	-0.021	-0.022	0.004	0.017	0.031	-0.020	0.051	0.100	0.009	-0.041
MO-GG	-0.081	0.035	-0.041	0.134	0.077	-0.001	-0.158	-0.050	-0.018	-0.059
GG-GH	-0.056	-0.111	-0.021	0.009	-0.016	0.040	-0.092	0.004	-0.017	-0.010
GH-IMA	**-0.203**	**0.524**	-0.092	0.007	-0.050	-0.017	**0.269**	0.052	0.124	0.096
IMA-PMA	-0.114	**-0.667**	0.034	-0.001	-0.028	-0.036	0.084	-0.042	0.083	0.050
PMA-CONL	-0.090	**0.298**	-0.021	-0.195	-0.081	**-0.226**	-0.117	-0.075	-0.117	-0.079
CONL-CONM	-0.046	-0.004	0.004	-0.024	-0.008	-0.050	-0.004	-0.009	0.013	0.037
CONL-COR	-0.087	-0.071	0.065	0.028	0.079	0.147	0.127	-0.199	**-0.247**	-0.128
COR-RAMA	-0.163	-0.036	-0.097	**-0.317**	-0.171	**-0.262**	**-0.248**	0.174	0.101	0.183
RAMA-PMA	-0.158	-0.079	0.032	-0.086	-0.013	-0.017	-0.053	-0.053	-0.140	0.096
COR-IMA	-0.189	-0.144	-0.032	**-0.280**	-0.174	**-0.386**	-0.192	0.044	0.119	0.061
MFO-ALV	-0.144	0.003	-0.128	-0.126	-0.064	**0.237**	0.035	0.081	**-0.221**	0.091
MO-MP3	-0.051	0.107	0.010	0.078	0.027	-0.042	-0.110	-0.033	0.068	-0.016
MP3-BDM1	-0.075	0.016	0.008	0.115	0.034	-0.033	0.005	0.018	0.120	-0.065
BDM1-RAMA	-0.092	-0.055	-0.122	-0.020	-0.034	0.104	0.161	0.031	0.034	-0.029
CON-MALV	**-0.263**	0.027	-0.157	**-0.202**	-0.104	0.140	0.144	-0.069	**-0.221**	0.070
MFO-CONM	-0.124	-0.048	-0.014	-0.069	-0.059	-0.186	0.074	**-0.234**	0.004	-0.077
RAMA-GH	-0.142	0.022	-0.063	0.139	0.011	-0.009	**0.296**	0.049	**0.315**	-0.036
MP3-MEN	-0.092	0.033	-0.116	0.088	0.042	0.082	-0.097	-0.045	-0.048	-0.017
MEN-GH	-0.033	0.028	-0.007	0.050	-0.011	-0.027	0.010	0.007	0.072	-0.024
ALV-IMA	-0.148	**0.224**	-0.059	-0.112	-0.105	-0.059	-0.066	-0.047	-0.115	0.052

ALV-RAMA	-0.020	-0.006	0.011	-0.016	-0.019	-0.048	-0.069	0.010	-0.004	-0.005
LDM1-BDM1	-0.128	-0.067	-0.093	-0.031	-0.035	0.060	0.157	0.034	0.089	0.025
LDM1-GH	-0.193	-0.010	-0.060	0.086	-0.014	-0.051	**0.317**	0.066	**0.369**	0.045
Percent variance explained (%)	36.365	8.974	4.633	4.224	3.827	3.068	2.687	2.504	2.377	2.314

Table S2.10. First 10 principal components for log-scale ratio skull data across Cercopithecini. Highlighted values depict relatively large loadings (<-0.2 and >0.2).

	PC1	PC2	PC3	PC4	PC5	PC6	PC7	PC8	PC9	PC10
IS-PM	0.044	0.185	0.080	-0.050	0.073	-0.023	-0.057	-0.033	0.031	0.119
IS-NSL	0.016	-0.099	0.019	0.046	-0.012	-0.154	-0.075	0.043	0.004	0.019
IS-PNS	-0.037	-0.020	0.038	0.023	0.005	-0.079	-0.024	0.007	0.028	0.062
PM-ZS	-0.024	-0.128	0.037	0.043	-0.092	-0.114	0.017	0.078	0.121	0.074
PM-ZI	-0.015	-0.093	0.055	0.030	-0.027	-0.061	0.019	0.017	0.054	0.022
PM-MT	-0.054	0.062	0.037	0.006	0.006	-0.041	-0.018	0.035	0.019	0.037
NSL-NA	0.012	0.007	-0.056	-0.094	0.045	0.122	**0.344**	**0.216**	**0.508**	**0.205**
NSL-ZS	-0.028	0.097	0.149	-0.013	**-0.346**	0.049	0.108	0.004	0.109	0.008
NSL-ZI	0.010	0.037	0.029	-0.033	-0.036	0.031	0.059	-0.046	0.018	0.012
NA-BR	-0.003	0.073	0.019	-0.050	0.006	0.018	-0.027	-0.018	-0.068	-0.073
NA-FM	-0.002	0.086	0.047	-0.038	-0.035	0.036	0.034	-0.001	0.027	-0.032
NA-PNS	0.028	0.050	0.014	-0.021	0.002	0.034	0.054	0.036	0.086	0.025
BR-ZAF	0.000	0.099	0.011	-0.030	0.010	0.053	0.022	-0.041	-0.027	-0.061
BR-APET	-0.011	0.109	0.048	-0.034	0.033	0.044	-0.027	-0.011	-0.037	-0.059
ZAF-FM	**0.256**	-0.021	**0.534**	**0.498**	**0.288**	**0.228**	-0.038	0.118	0.034	0.028
TS-MT	0.048	**-0.271**	-0.048	0.024	-0.048	0.032	-0.029	-0.052	0.035	-0.033
ZAF-BA	-0.057	0.047	-0.099	-0.137	-0.034	-0.021	-0.043	-0.015	-0.024	-0.039
ZAF-EAM	-0.045	0.001	-0.146	-0.175	-0.034	-0.005	-0.062	-0.010	-0.030	-0.017
ZAF-ZYGO	-0.060	0.033	-0.170	**-0.257**	-0.096	-0.076	-0.039	0.039	-0.012	0.027
AS-EAM	-0.035	0.086	0.020	-0.063	-0.006	0.007	0.077	-0.023	0.022	-0.122
FM-ZS	0.031	0.018	-0.110	-0.148	**0.357**	0.011	-0.024	0.059	-0.013	-0.058
FM-MT	0.061	-0.014	-0.010	-0.011	0.039	0.027	-0.039	0.037	-0.011	0.017

ZS-ZI	0.018	-0.034	-0.064	-0.002	**0.207**	0.009	0.018	-0.077	-0.031	0.112
ZI-MT	0.046	0.197	-0.015	0.048	0.040	0.035	-0.158	0.161	-0.060	0.049
ZI-ZYGO	0.010	-0.013	-0.025	-0.048	-0.015	-0.009	-0.061	0.079	0.060	0.035
NA-ORB	0.046	0.014	-0.057	-0.064	0.067	0.083	**0.275**	**0.234**	**0.371**	0.021
MT-PNS	-0.011	-0.169	-0.024	0.032	-0.009	0.031	0.016	-0.108	-0.021	-0.046
PNS-APET	0.051	-0.071	-0.005	-0.022	0.010	0.104	-0.053	-0.009	0.074	-0.034
APET-BA	0.005	0.007	0.012	-0.018	0.028	-0.008	-0.018	0.002	-0.024	-0.005
APET-TS	-0.032	0.095	-0.014	-0.055	-0.015	-0.003	0.018	-0.016	-0.016	-0.096
BA-EAM	-0.013	0.031	0.006	-0.019	0.013	0.034	-0.005	0.001	-0.022	-0.047
EAM-ZYGO	0.009	-0.085	-0.083	0.016	0.060	0.141	-0.077	-0.093	-0.115	-0.035
ORB-ZS	0.047	**0.261**	**0.310**	0.018	**-0.600**	0.124	-0.002	-0.149	-0.084	-0.030
LD-AS	-0.022	0.140	0.045	-0.048	0.046	0.060	-0.033	-0.054	-0.045	-0.043
BR-LD	-0.006	0.106	0.073	-0.048	0.026	0.091	-0.064	0.046	-0.003	-0.070
OPI-LD	-0.043	0.122	0.047	-0.062	0.019	0.030	-0.048	-0.057	-0.014	-0.044
ZAF-AS	-0.036	0.040	-0.082	-0.120	-0.024	0.016	0.001	-0.043	-0.006	-0.053
JP-AS	-0.050	0.075	0.011	-0.084	0.004	0.005	0.018	0.000	0.003	-0.074
BA-OPI	0.015	0.135	0.041	-0.094	0.038	0.142	0.041	-0.009	-0.067	-0.116
MO-GG	-0.108	0.009	0.191	0.044	0.016	**-0.439**	**-0.315**	0.006	**0.262**	0.052
GG-GH	**0.237**	-0.009	-0.154	0.101	-0.186	-0.066	**0.375**	**0.252**	**-0.396**	**0.481**
GH-IMA	**-0.267**	-0.085	0.052	-0.003	0.044	0.069	0.027	-0.019	-0.011	-0.084
IMA-PMA	**0.674**	0.039	-0.136	-0.100	0.007	-0.190	-0.140	**-0.232**	0.055	0.002
PMA-CONL	**-0.365**	-0.070	-0.034	0.090	0.020	**0.339**	-0.081	0.019	-0.180	**0.211**
CONL-CONM	-0.012	0.004	-0.083	0.033	0.012	0.013	0.051	-0.097	-0.114	0.086
CONL-COR	0.106	-0.037	-0.048	-0.132	-0.004	0.086	**-0.252**	**0.586**	**-0.212**	-0.195
COR-RAMA	-0.011	-0.093	-0.134	0.158	-0.025	0.037	0.025	**-0.361**	0.094	**0.208**
RAMA-PMA	0.045	-0.041	-0.058	-0.008	-0.014	-0.016	-0.088	0.006	-0.015	0.073
COR-IMA	0.041	-0.034	-0.077	0.038	0.019	0.042	-0.046	-0.192	-0.028	0.173
MFO-ALV	0.032	**-0.443**	-0.048	0.083	**-0.240**	0.018	0.036	0.069	0.086	**-0.266**
MO-MP3	**-0.250**	0.116	0.068	0.019	0.034	**-0.259**	-0.069	-0.057	0.156	**0.205**
MP3-BDM1	-0.029	0.091	0.044	-0.025	0.045	-0.050	-0.005	0.031	-0.026	0.030

BDM1-RAMA	0.107	**-0.278**	0.076	-0.039	-0.041	0.068	0.072	-0.123	-0.009	**-0.201**
CON-MALV	-0.015	**-0.264**	-0.024	0.016	-0.033	0.057	-0.063	-0.023	-0.001	-0.017
MFO-CONM	0.011	-0.010	-0.028	-0.005	0.156	0.060	-0.199	-0.101	-0.131	**0.266**
RAMA-GH	-0.011	-0.006	0.064	-0.063	0.070	0.016	0.065	-0.071	-0.001	-0.094
MP3-MEN	-0.064	-0.144	0.182	0.056	-0.044	**-0.509**	0.056	**0.203**	**-0.220**	-0.004
MEN-GH	-0.091	0.100	0.052	0.083	**0.257**	**-0.255**	**0.541**	-0.168	**-0.255**	**-0.311**
ALV-IMA	**-0.232**	-0.194	-0.048	0.105	-0.031	-0.023	-0.050	0.006	-0.066	0.103
ALV-RAMA	-0.039	**0.293**	**-0.536**	**0.649**	-0.106	-0.058	-0.085	0.081	0.116	**-0.267**
LDM1-BDM1	0.069	-0.133	0.000	-0.016	-0.006	0.040	0.013	-0.089	0.013	-0.080
LDM1-GH	0.003	-0.006	0.007	-0.030	0.057	0.017	0.032	-0.075	0.006	-0.054
Percent variance explained (%)	12.791	8.630	7.021	6.104	5.249	4.573	3.787	3.603	3.346	3.151

Table S2.11. First 10 principal components for raw skull data across Asian colobines. Highlighted values depict relatively large loadings (<-0.2 and >0.2).

	PC1	PC2	PC3	PC4	PC5	PC6	PC7	PC8	PC9	PC10
IS-PM	-0.018	0.013	-0.010	0.003	-0.011	-0.023	-0.020	0.001	-0.048	0.031
IS-NSL	-0.179	-0.023	-0.004	-0.080	0.054	0.020	-0.052	**-0.215**	-0.171	0.007
IS-PNS	**-0.202**	0.042	0.061	-0.089	-0.026	-0.076	-0.081	-0.131	**-0.220**	0.051
PM-ZS	-0.183	-0.027	-0.007	-0.064	0.100	0.003	-0.050	-0.093	-0.032	-0.020
PM-ZI	-0.163	-0.003	0.022	-0.113	0.140	-0.004	-0.017	-0.124	-0.109	0.055
PM-MT	-0.097	-0.038	0.011	-0.007	-0.119	-0.189	-0.037	-0.058	**-0.228**	0.069
NSL-NA	-0.050	0.028	-0.016	-0.040	0.074	-0.045	0.025	-0.188	0.001	-0.025
NSL-ZS	-0.058	-0.033	-0.090	0.037	0.085	-0.025	-0.052	0.109	**0.243**	0.005
NSL-ZI	-0.120	0.023	0.031	-0.027	0.076	-0.001	0.019	-0.047	-0.022	0.032
NA-BR	-0.173	0.012	-0.185	-0.173	**-0.286**	**0.328**	-0.134	**0.395**	**-0.220**	-0.070
NA-FM	-0.087	0.021	0.020	0.016	-0.018	0.018	-0.011	0.023	0.029	0.026
NA-PNS	-0.132	0.041	0.037	-0.034	-0.054	0.002	-0.034	-0.055	-0.069	0.051
BR-ZAF	-0.152	0.014	-0.062	**-0.281**	**-0.446**	0.092	-0.162	0.123	0.096	**-0.269**
BR-APET	-0.094	-0.029	0.123	-0.034	**-0.286**	0.056	**-0.214**	0.033	0.100	**0.201**
ZAF-FM	-0.012	-0.017	**0.220**	**-0.312**	0.081	0.099	-0.170	0.061	-0.080	**0.204**

TS-MT	**-0.257**	0.007	0.033	-0.081	**0.222**	**0.205**	0.059	-0.059	0.065	-0.134
ZAF-BA	**-0.219**	-0.006	-0.174	**0.355**	**-0.200**	-0.072	0.081	-0.075	0.019	-0.095
ZAF-EAM	-0.194	-0.034	-0.157	**0.360**	-0.081	0.065	0.085	-0.008	-0.087	-0.050
ZAF-ZYGO	-0.103	0.013	-0.099	**0.417**	0.118	0.159	0.071	0.165	-0.155	**0.316**
AS-EAM	-0.102	0.017	0.066	0.061	0.008	-0.082	-0.197	0.024	0.151	-0.179
FM-ZS	-0.055	-0.012	0.042	-0.046	-0.008	0.078	0.034	-0.111	-0.192	0.042
FM-MT	-0.164	-0.012	0.076	-0.082	-0.046	0.091	-0.030	-0.020	-0.116	0.154
ZS-ZI	-0.074	0.034	0.115	-0.037	-0.029	0.008	0.050	-0.084	-0.159	0.000
ZI-MT	-0.030	0.008	0.001	0.036	-0.130	-0.069	-0.027	-0.011	-0.142	0.099
ZI-ZYGO	-0.124	-0.007	0.030	0.195	0.069	0.135	-0.045	**0.228**	-0.068	0.156
NA-ORB	-0.060	0.025	-0.051	-0.043	0.027	-0.056	-0.026	-0.183	0.055	-0.011
MT-PNS	-0.106	0.010	0.033	-0.064	0.056	0.041	-0.010	-0.036	0.055	-0.026
PNS-APET	-0.123	-0.053	-0.073	0.021	0.115	0.041	0.044	-0.025	0.028	-0.102
APET-BA	-0.060	-0.009	0.030	0.009	-0.027	0.007	-0.014	-0.024	0.006	0.046
APET-TS	-0.027	0.010	0.033	-0.019	-0.033	0.009	0.017	0.011	-0.026	-0.026
BA-EAM	-0.108	0.010	0.048	0.010	-0.027	-0.021	-0.048	-0.021	0.092	0.005
EAM-ZYGO	-0.117	-0.073	-0.014	-0.087	-0.114	-0.066	0.120	-0.179	0.019	**-0.210**
ORB-ZS	-0.028	-0.036	-0.045	0.032	0.027	-0.002	-0.056	0.158	**0.201**	0.088
LD-AS	-0.071	0.001	-0.124	-0.095	-0.190	0.066	0.076	-0.099	0.151	**0.415**
BR-LD	-0.047	-0.109	**0.487**	**0.281**	0.031	**-0.209**	**-0.532**	-0.059	0.040	0.031
OPI-LD	-0.091	-0.034	-0.159	-0.021	**-0.221**	-0.079	0.008	-0.177	**0.373**	**0.416**
ZAF-AS	**-0.253**	-0.062	-0.055	**0.251**	-0.173	-0.099	-0.094	0.024	0.081	**-0.332**
JP-AS	-0.093	-0.020	0.008	0.066	-0.012	-0.100	-0.124	-0.022	0.154	-0.044
BA-OPI	-0.025	-0.007	-0.015	0.026	0.003	0.064	-0.058	0.066	-0.079	0.002
MO-GG	-0.096	-0.020	-0.007	-0.088	-0.091	-0.054	-0.074	**-0.217**	-0.061	0.051
GG-GH	-0.047	0.106	0.036	0.033	0.037	0.016	0.026	0.088	-0.023	-0.011
GH-IMA	**-0.208**	**-0.506**	-0.043	-0.080	**0.217**	-0.162	-0.031	**0.252**	-0.049	0.081
IMA-PMA	-0.122	**0.654**	-0.074	0.057	0.067	0.006	-0.076	-0.115	0.078	0.036
PMA-CONL	-0.067	**-0.297**	0.136	-0.066	-0.153	-0.166	**0.395**	0.048	0.124	0.039
CONL-CONM	-0.062	0.027	0.013	-0.019	-0.021	0.012	-0.013	0.002	0.020	0.016

CONL-COR	-0.081	-0.110	-0.169	0.072	0.017	0.125	-0.019	**-0.212**	-0.093	0.107
COR-RAMA	-0.133	0.171	**0.453**	0.032	-0.061	0.024	**0.266**	**0.216**	-0.041	-0.030
RAMA-PMA	-0.148	-0.038	0.157	0.076	-0.043	0.188	0.077	-0.086	-0.043	0.076
COR-IMA	-0.166	**0.240**	**0.261**	-0.026	-0.076	**-0.272**	**0.348**	0.179	0.122	0.056
MFO-ALV	-0.182	-0.011	0.098	-0.025	0.176	**0.327**	-0.031	0.005	**0.282**	-0.006
MO-MP3	-0.044	-0.026	-0.033	-0.006	-0.016	-0.088	-0.022	-0.016	-0.053	0.022
MP3-BDM1	-0.039	-0.009	-0.028	0.011	-0.049	-0.103	-0.017	0.003	-0.142	0.049
BDM1-RAMA	-0.121	0.019	-0.106	-0.119	0.197	-0.045	0.006	0.033	0.118	-0.063
CON-MALV	**-0.257**	-0.059	0.058	-0.028	0.198	**0.218**	0.130	-0.040	0.119	-0.067
MFO-CONM	-0.087	-0.014	-0.057	-0.031	0.020	-0.181	0.163	-0.101	**-0.280**	-0.088
RAMA-GH	-0.155	0.080	**-0.249**	-0.116	**0.216**	**-0.312**	-0.128	0.180	-0.002	0.073
MP3-MEN	-0.141	-0.010	0.015	-0.064	0.039	-0.019	-0.028	-0.194	0.054	-0.046
MEN-GH	-0.034	0.044	0.067	0.013	-0.038	0.026	0.043	0.004	-0.065	0.046
ALV-IMA	-0.125	-0.080	0.050	-0.037	-0.032	-0.136	0.125	-0.011	0.087	0.063
ALV-RAMA	-0.020	0.067	0.101	0.042	-0.080	0.004	0.019	0.014	-0.112	0.049
LDM1-BDM1	-0.115	0.080	-0.028	-0.096	0.065	-0.032	0.026	0.082	0.040	-0.027
LDM1-GH	-0.160	**0.207**	-0.175	-0.114	0.092	**-0.326**	-0.084	**0.268**	-0.080	0.096
Percent variance explained (%)	23.644	7.988	5.746	5.497	4.822	4.317	3.616	3.401	3.150	2.814

Table S2.12. First 10 principal components for log-scale ratio skull data across Asian colobines. Highlighted values depict relatively large loadings (<-0.2 and >0.2).

	PC1	PC2	PC3	PC4	PC5	PC6	PC7	PC8	PC9	PC10
IS-PM	-0.154	0.013	0.026	0.076	0.063	0.114	0.080	0.021	0.015	-0.051
IS-NSL	0.099	0.017	-0.027	-0.029	-0.016	0.158	0.043	0.079	0.040	-0.066
IS-PNS	0.044	-0.019	0.004	-0.027	0.005	0.081	0.061	0.018	0.015	-0.048
PM-ZS	0.146	-0.009	-0.020	-0.027	-0.047	0.119	0.061	0.015	-0.102	-0.078
PM-ZI	0.113	-0.007	0.017	-0.024	-0.001	0.088	0.053	-0.013	-0.049	-0.029
PM-MT	-0.056	-0.055	0.027	0.015	0.034	0.091	0.041	0.010	-0.008	-0.018
NSL-NA	0.034	-0.008	-0.089	0.116	0.012	0.032	0.020	**-0.240**	**-0.557**	-0.132

NSL-ZS	-0.018	0.028	0.191	0.000	-0.124	0.014	0.074	-0.079	**-0.232**	-0.095
NSL-ZI	-0.019	0.001	0.035	0.036	0.004	0.001	0.038	-0.055	-0.060	0.016
NA-BR	-0.064	-0.020	0.034	0.057	0.022	-0.037	-0.031	0.034	0.087	0.028
NA-FM	-0.077	-0.001	0.062	0.056	0.007	-0.029	-0.011	-0.045	-0.020	0.021
NA-PNS	-0.043	0.021	-0.002	0.044	0.012	-0.008	-0.023	-0.056	-0.109	-0.011
BR-ZAF	-0.075	-0.012	0.035	0.046	0.034	-0.044	-0.020	-0.031	0.028	0.024
BR-APET	-0.088	-0.026	0.044	0.046	0.053	-0.042	-0.048	0.019	0.045	0.050
ZAF-FM	-0.019	**0.359**	0.124	**-0.478**	**0.681**	0.006	-0.075	-0.004	-0.079	0.044
TS-MT	**0.260**	0.067	-0.054	-0.081	-0.072	-0.086	-0.012	-0.021	0.042	-0.054
ZAF-BA	-0.042	-0.088	-0.004	0.131	-0.106	-0.033	-0.016	0.034	0.035	-0.022
ZAF-EAM	-0.007	-0.067	-0.024	0.121	-0.097	-0.045	-0.028	0.032	0.027	-0.048
ZAF-ZYGO	-0.015	-0.071	-0.029	0.151	-0.134	-0.054	-0.026	0.063	0.014	-0.008
AS-EAM	-0.074	-0.038	0.039	0.068	0.001	-0.063	-0.008	-0.030	-0.013	0.063
FM-ZS	-0.024	-0.045	-0.172	0.147	**0.200**	-0.039	-0.090	-0.037	0.030	0.049
FM-MT	0.028	0.044	-0.030	-0.015	0.034	-0.018	-0.050	0.009	-0.008	-0.006
ZS-ZI	0.044	-0.038	-0.181	0.052	0.086	0.014	0.043	-0.021	0.063	0.105
ZI-MT	-0.187	-0.030	-0.046	-0.007	0.104	0.057	-0.097	0.116	-0.025	-0.055
ZI-ZYGO	0.006	0.019	-0.023	0.021	-0.018	-0.078	-0.034	0.055	0.043	-0.014
NA-ORB	-0.035	0.004	-0.098	0.128	0.074	0.013	-0.054	**-0.259**	**-0.482**	-0.088
MT-PNS	**0.235**	0.026	-0.015	-0.109	-0.054	-0.101	0.009	-0.052	0.087	-0.011
PNS-APET	0.041	0.042	-0.008	0.025	0.023	-0.028	-0.019	-0.025	-0.032	-0.092
APET-BA	-0.026	-0.019	0.005	0.040	0.020	-0.036	-0.021	0.030	0.010	0.009
APET-TS	-0.071	-0.064	0.010	0.069	-0.009	-0.051	-0.047	-0.045	-0.012	0.101
BA-EAM	-0.033	-0.017	0.013	0.028	0.016	-0.048	-0.025	0.006	0.020	0.019
EAM-ZYGO	0.026	-0.019	-0.050	-0.016	0.013	-0.039	-0.057	-0.032	0.067	-0.084
ORB-ZS	**-0.259**	**0.289**	**0.684**	-0.151	**-0.353**	-0.080	0.006	-0.023	-0.004	-0.058
LD-AS	-0.091	-0.014	0.053	0.049	0.066	-0.013	-0.008	-0.023	0.066	-0.008
BR-LD	-0.093	-0.019	0.053	0.038	0.054	-0.088	-0.083	-0.035	-0.011	0.119
OPI-LD	-0.073	-0.032	0.057	0.055	0.045	-0.020	-0.018	0.015	0.084	-0.038
ZAF-AS	-0.037	-0.055	0.003	0.095	-0.051	-0.052	-0.016	-0.022	0.019	-0.002

JP-AS	-0.052	-0.054	0.038	0.072	0.015	-0.030	-0.028	-0.006	0.029	0.031
BA-OPI	-0.132	-0.011	0.045	0.112	0.035	-0.088	-0.077	-0.068	0.005	0.080
MO-GG	0.057	-0.107	0.083	-0.025	0.082	**0.457**	0.144	0.081	0.124	**-0.347**
GG-GH	-0.043	**0.216**	-0.138	-0.012	-0.152	-0.017	**0.285**	**0.222**	**-0.201**	**0.535**
GH-IMA	0.092	**-0.249**	0.113	-0.037	0.072	-0.089	-0.042	-0.003	0.012	-0.017
IMA-PMA	-0.091	**0.581**	**-0.271**	**0.208**	-0.108	0.113	-0.007	-0.091	**0.259**	-0.145
PMA-CONL	0.044	**-0.372**	0.117	-0.195	0.044	**-0.218**	-0.109	-0.043	0.053	0.183
CONL-CONM	-0.012	-0.007	-0.050	-0.030	-0.014	-0.045	0.023	0.003	0.082	0.055
CONL-COR	-0.013	0.067	-0.042	0.138	0.045	-0.136	**-0.353**	**0.678**	-0.108	-0.161
COR-RAMA	0.040	-0.009	-0.113	-0.195	-0.125	-0.017	0.082	**-0.340**	0.169	0.051
RAMA-PMA	0.019	0.046	-0.065	-0.031	-0.040	-0.013	-0.011	0.049	0.062	-0.088
COR-IMA	-0.012	0.036	-0.065	-0.018	-0.039	-0.022	0.018	-0.161	0.158	0.059
MFO-ALV	**0.433**	0.111	-0.018	-0.182	-0.175	**-0.214**	0.026	0.128	-0.114	**-0.228**
MO-MP3	-0.083	**-0.207**	0.086	0.001	0.059	**0.248**	**0.235**	0.019	0.090	**-0.242**
MP3-BDM1	-0.083	-0.041	0.011	0.038	0.052	0.100	0.014	0.015	-0.011	0.033
BDM1-RAMA	**0.263**	0.135	0.050	0.085	-0.009	-0.066	-0.006	-0.077	0.032	0.092
CON-MALV	**0.235**	0.015	-0.014	-0.071	-0.050	-0.099	-0.023	0.003	0.068	-0.070
MFO-CONM	-0.015	-0.033	-0.036	0.013	0.059	0.042	-0.103	-0.100	**0.246**	0.111
RAMA-GH	0.002	0.000	0.042	0.111	0.067	-0.010	0.006	-0.044	0.043	0.036
MP3-MEN	**0.246**	-0.015	0.125	-0.019	-0.117	**0.600**	-0.149	0.150	-0.088	**0.460**
MEN-GH	-0.135	-0.054	-0.115	-0.089	0.077	-0.195	**0.722**	**0.264**	-0.027	0.015
ALV-IMA	0.137	-0.194	0.025	-0.116	-0.029	-0.029	0.010	0.042	0.031	0.019
ALV-RAMA	**-0.386**	-0.097	**-0.409**	**-0.571**	**-0.345**	0.109	**-0.235**	0.025	-0.089	-0.074
LDM1-BDM1	0.110	0.077	-0.028	0.015	-0.013	-0.032	-0.015	-0.079	0.067	0.045
LDM1-GH	-0.016	0.008	-0.017	0.050	0.029	-0.007	-0.018	-0.075	0.072	0.035
Percent variance explained (%)	9.370	9.153	7.313	6.330	5.921	4.528	4.109	3.632	3.314	3.011

Table S2.13. First 10 principal components for raw skull data across African colobines. Highlighted values depict relatively large loadings (<-0.2 and >0.2).

	PC1	PC2	PC3	PC4	PC5	PC6	PC7	PC8	PC9	PC10

IS-PM	-0.019	-0.014	-0.002	-0.001	-0.009	-0.019	-0.030	-0.013	-0.056	0.029
IS-NSL	-0.184	0.033	0.037	0.074	0.081	0.020	-0.081	**0.214**	-0.138	-0.002
IS-PNS	**-0.203**	-0.033	-0.082	0.084	0.044	-0.054	-0.104	0.148	**-0.225**	0.054
PM-ZS	-0.184	0.026	0.043	0.019	0.107	-0.009	-0.052	0.096	0.004	-0.027
PM-ZI	-0.161	0.026	0.031	0.062	0.153	-0.003	-0.022	0.119	-0.086	0.027
PM-MT	-0.096	0.054	-0.062	0.026	-0.097	-0.158	-0.051	0.053	**-0.249**	0.028
NSL-NA	-0.046	-0.010	0.048	0.015	0.057	-0.089	-0.036	0.172	-0.014	0.025
NSL-ZS	-0.062	0.010	0.120	-0.058	0.023	-0.057	-0.003	-0.137	**0.235**	-0.023
NSL-ZI	-0.119	-0.002	0.000	0.017	0.074	-0.007	0.016	0.035	-0.037	0.008
NA-BR	-0.178	-0.074	0.091	0.180	**-0.304**	**0.396**	-0.131	**-0.330**	**-0.213**	-0.163
NA-FM	-0.086	-0.026	-0.024	-0.020	-0.011	0.019	-0.001	-0.029	0.034	0.025
NA-PNS	-0.130	-0.036	-0.058	0.029	-0.029	0.000	-0.044	0.061	-0.066	0.063
BR-ZAF	-0.152	-0.052	-0.051	**0.297**	**-0.388**	0.098	-0.174	-0.119	0.131	**-0.311**
BR-APET	-0.087	0.031	**-0.202**	0.057	**-0.247**	0.085	-0.177	-0.060	0.129	0.185
ZAF-FM	-0.004	0.060	-0.139	**0.311**	0.165	0.174	-0.197	-0.066	-0.070	**0.202**
TS-MT	**-0.259**	-0.010	0.059	0.029	**0.231**	0.185	0.048	0.073	0.127	-0.091
ZAF-BA	**-0.229**	-0.045	0.054	**-0.316**	**-0.273**	-0.148	0.113	0.052	-0.004	-0.110
ZAF-EAM	**-0.204**	0.005	0.091	**-0.342**	-0.174	0.034	0.108	0.016	-0.099	-0.065
ZAF-ZYGO	-0.099	-0.017	0.077	**-0.421**	0.011	0.182	0.119	-0.165	-0.164	**0.290**
AS-EAM	-0.100	-0.038	-0.090	-0.059	0.045	-0.085	-0.198	0.004	0.151	-0.096
FM-ZS	-0.057	0.027	-0.036	0.044	0.011	0.073	-0.010	0.108	-0.196	0.063
FM-MT	-0.161	0.028	-0.068	0.084	-0.022	0.087	-0.043	-0.010	-0.138	0.136
ZS-ZI	-0.070	-0.002	-0.124	0.050	0.030	0.029	0.016	0.099	-0.157	0.006
ZI-MT	-0.023	0.002	-0.044	-0.002	-0.134	-0.058	-0.029	0.002	-0.189	0.073
ZI-ZYGO	-0.123	0.010	-0.029	**-0.209**	0.044	0.186	-0.008	**-0.228**	-0.033	0.151
NA-ORB	-0.056	-0.028	0.080	0.033	0.000	-0.096	-0.066	0.174	0.061	0.019
MT-PNS	-0.107	-0.013	-0.010	0.054	0.074	0.036	-0.027	0.036	0.059	-0.009
PNS-APET	-0.126	0.033	0.119	-0.050	0.071	0.010	0.035	-0.015	0.047	-0.110
APET-BA	-0.060	0.015	-0.039	-0.001	-0.019	0.006	-0.009	0.021	0.001	0.045
APET-TS	-0.027	0.002	-0.029	0.032	-0.024	0.025	0.000	0.017	-0.018	0.000

BA-EAM	-0.109	-0.009	-0.058	0.002	-0.005	-0.021	-0.057	0.019	0.120	0.043
EAM-ZYGO	-0.120	0.067	0.003	0.130	-0.110	-0.101	0.075	0.195	-0.033	-0.196
ORB-ZS	-0.028	0.034	0.051	-0.036	-0.010	-0.004	0.011	-0.197	**0.210**	0.026
LD-AS	-0.071	-0.025	0.079	0.130	**-0.236**	0.064	0.078	0.035	0.155	**0.408**
BR-LD	-0.049	0.179	**-0.461**	**-0.301**	0.119	-0.175	**-0.504**	-0.064	0.122	0.053
OPI-LD	-0.092	0.006	0.085	0.079	**-0.278**	-0.106	0.007	0.123	**0.335**	**0.464**
ZAF-AS	**-0.263**	0.020	-0.036	**-0.222**	-0.195	-0.127	-0.093	-0.004	0.082	**-0.284**
JP-AS	-0.094	0.020	-0.012	-0.038	-0.019	-0.120	-0.139	0.017	0.157	0.037
BA-OPI	-0.022	0.006	0.026	-0.051	-0.016	0.066	-0.061	-0.078	-0.060	0.017
MO-GG	-0.101	0.027	-0.010	0.106	-0.064	-0.048	-0.098	**0.203**	-0.041	0.031
GG-GH	-0.043	-0.101	-0.039	-0.046	0.048	0.021	0.051	-0.095	-0.012	-0.007
GH-IMA	**-0.211**	**0.492**	0.151	0.069	0.174	-0.096	-0.036	**-0.264**	-0.080	0.065
IMA-PMA	-0.110	**-0.652**	0.016	-0.042	0.080	-0.031	-0.062	0.117	0.039	0.087
PMA-CONL	-0.070	**0.329**	-0.122	0.129	-0.134	-0.160	**0.387**	-0.062	0.071	0.030
CONL-CONM	-0.059	-0.022	-0.016	0.026	-0.015	0.012	-0.020	-0.010	0.015	0.026
CONL-COR	-0.090	0.081	0.191	-0.105	-0.064	0.096	-0.026	**0.213**	-0.120	0.157
COR-RAMA	-0.124	-0.117	**-0.487**	0.030	0.117	0.047	**0.276**	-0.143	-0.031	-0.053
RAMA-PMA	-0.149	0.056	-0.143	-0.052	-0.007	0.182	0.073	0.130	-0.039	0.079
COR-IMA	-0.157	-0.183	**-0.310**	0.120	0.038	**-0.233**	**0.369**	-0.151	0.048	0.051
MFO-ALV	-0.177	-0.004	-0.006	-0.002	0.185	**0.321**	0.030	0.042	**0.249**	-0.015
MO-MP3	-0.046	0.020	0.017	0.016	-0.014	-0.080	-0.035	0.005	-0.060	0.018
MP3-BDM1	-0.042	0.016	-0.005	0.005	-0.046	-0.087	-0.036	-0.025	-0.162	0.044
BDM1-RAMA	-0.117	-0.012	0.170	0.072	0.156	-0.042	0.025	-0.055	0.136	-0.087
CON-MALV	**-0.254**	0.049	0.037	-0.008	0.192	0.187	0.134	0.070	0.155	-0.040
MFO-CONM	-0.089	0.030	0.050	0.024	0.018	-0.184	0.086	0.076	-0.185	-0.061
RAMA-GH	-0.150	-0.093	**0.269**	0.090	0.152	**-0.273**	-0.124	**-0.276**	-0.049	0.084
MP3-MEN	-0.142	0.042	0.026	0.057	0.051	-0.031	-0.037	0.166	0.108	-0.052
MEN-GH	-0.034	-0.027	-0.068	-0.001	-0.022	0.016	0.052	-0.019	-0.073	0.040
ALV-IMA	-0.125	0.086	-0.034	0.087	-0.020	-0.108	0.144	0.015	0.030	0.057
ALV-RAMA	-0.018	-0.064	-0.135	-0.010	-0.034	-0.003	0.013	0.004	-0.134	0.054

LDM1-BDM1	-0.106	-0.077	0.046	0.074	0.062	-0.030	0.042	-0.073	0.056	-0.051
LDM1-GH	-0.148	**-0.234**	0.145	0.120	0.077	**-0.300**	-0.086	**-0.326**	-0.132	0.114
Percent variance explained (%)	24.668	7.890	5.746	5.186	5.008	4.326	3.714	3.439	3.133	2.833

Table S2.14. First 10 principal components for log-scale ratio skull data across African colobines. Highlighted values depict relatively large loadings (<-0.2 and >0.2).

	PC1	PC2	PC3	PC4	PC5	PC6	PC7	PC8	PC9	PC10
IS-PM	0.068	0.084	0.026	0.016	0.104	0.012	-0.024	-0.041	0.116	-0.105
IS-NSL	-0.069	-0.067	0.027	0.010	-0.033	0.021	0.053	-0.152	-0.118	-0.080
IS-PNS	0.009	-0.037	0.027	0.017	0.023	0.012	-0.028	-0.045	0.001	-0.032
PM-ZS	-0.076	-0.055	-0.059	0.012	-0.088	0.082	0.027	0.014	0.009	-0.028
PM-ZI	-0.062	-0.043	0.006	0.006	-0.008	0.039	0.010	0.014	-0.008	0.000
PM-MT	0.046	0.081	0.066	0.040	0.017	-0.017	-0.058	-0.032	0.040	-0.051
NSL-NA	0.011	-0.061	-0.008	**0.335**	0.100	**0.248**	0.041	**0.580**	**0.407**	0.137
NSL-ZS	-0.078	**0.260**	-0.137	-0.097	-0.037	0.154	-0.034	0.015	-0.036	0.065
NSL-ZI	-0.014	0.013	0.008	-0.012	0.042	-0.029	-0.028	-0.001	-0.019	0.030
NA-BR	0.011	0.037	0.019	-0.056	0.054	-0.026	0.038	-0.064	-0.023	-0.015
NA-FM	0.014	0.059	-0.009	-0.018	0.050	-0.029	-0.023	-0.049	-0.012	0.042
NA-PNS	0.025	0.013	0.020	0.003	0.054	-0.007	0.009	0.027	0.022	0.026
BR-ZAF	0.019	0.053	0.042	0.001	0.027	0.042	-0.095	-0.063	-0.005	0.046
BR-APET	0.052	0.063	0.069	-0.043	0.023	-0.036	-0.029	-0.053	0.067	0.023
ZAF-FM	0.080	-0.144	**0.556**	**-0.501**	0.153	**0.329**	0.120	0.151	-0.075	-0.195
TS-MT	-0.152	-0.198	-0.074	-0.046	-0.045	-0.030	0.091	0.043	-0.112	0.075
ZAF-BA	-0.013	0.064	-0.106	0.097	-0.004	-0.124	-0.012	-0.095	0.041	0.079
ZAF-EAM	-0.032	0.052	-0.124	0.080	-0.034	-0.168	0.105	-0.076	0.033	0.034
ZAF-ZYGO	0.021	0.089	-0.190	-0.036	0.032	**-0.247**	**0.269**	-0.022	0.097	-0.007
AS-EAM	-0.011	0.025	0.008	-0.058	0.085	0.034	-0.017	-0.064	0.007	0.062
FM-ZS	0.037	-0.065	0.156	0.063	0.087	-0.103	0.103	-0.032	0.066	0.092
FM-MT	0.017	-0.002	0.063	-0.049	0.005	-0.006	0.054	-0.025	0.015	-0.012
ZS-ZI	0.068	**-0.288**	0.155	0.054	0.084	**-0.252**	-0.118	0.024	0.067	-0.008

ZI-MT	0.137	0.138	0.086	0.018	0.040	-0.008	0.063	-0.038	0.102	-0.130
ZI-ZYGO	0.014	0.017	-0.070	-0.124	0.013	-0.118	0.175	-0.004	0.039	0.022
NA-ORB	-0.057	0.020	0.061	**0.238**	**0.254**	0.131	0.033	0.113	-0.053	0.106
MT-PNS	-0.096	-0.161	-0.015	-0.053	-0.037	0.047	-0.022	-0.047	-0.130	0.088
PNS-APET	-0.098	0.028	-0.056	0.001	-0.028	0.008	0.080	0.052	-0.111	-0.060
APET-BA	-0.004	0.001	0.081	-0.026	0.060	-0.046	0.003	-0.044	0.108	0.160
APET-TS	0.064	-0.046	0.068	0.042	0.023	-0.078	-0.053	0.078	0.144	**0.300**
BA-EAM	0.011	-0.007	0.006	-0.018	0.006	0.017	-0.006	-0.002	0.016	0.105
EAM-ZYGO	-0.036	-0.017	0.096	0.119	-0.093	0.005	-0.061	-0.016	-0.120	0.025
ORB-ZS	-0.049	**0.472**	**-0.243**	**-0.330**	**-0.274**	**0.319**	-0.110	0.054	0.119	-0.021
LD-AS	0.011	0.110	0.042	0.038	0.055	0.031	-0.025	-0.123	0.040	0.114
BR-LD	0.088	0.091	0.091	-0.078	-0.032	-0.045	-0.057	-0.003	0.114	0.040
OPI-LD	-0.038	0.118	0.049	0.083	0.039	0.040	-0.061	-0.187	0.057	0.100
ZAF-AS	-0.026	0.052	-0.039	0.038	-0.001	-0.078	-0.016	-0.088	-0.004	0.066
JP-AS	-0.015	0.047	0.015	0.019	0.004	0.038	-0.017	-0.027	0.045	0.117
BA-OPI	0.053	0.132	-0.007	-0.023	0.054	-0.052	0.169	0.031	0.008	0.058
MO-GG	-0.056	-0.082	0.152	**0.240**	-0.091	**0.206**	-0.137	**-0.277**	0.194	-0.184
GG-GH	0.127	-0.115	**-0.337**	-0.155	0.135	**-0.232**	**-0.222**	**0.331**	0.047	**-0.483**
GH-IMA	-0.145	0.065	0.171	-0.039	**-0.207**	-0.105	0.096	0.082	-0.075	-0.012
IMA-PMA	0.079	-0.098	**-0.293**	0.017	**0.442**	**0.215**	-0.017	-0.192	-0.091	0.010
PMA-CONL	-0.021	0.147	**0.228**	0.019	**-0.364**	**-0.299**	-0.178	**0.280**	-0.025	0.094
CONL-CONM	0.009	-0.046	-0.025	-0.023	-0.022	0.027	-0.035	-0.080	0.046	-0.043
CONL-COR	-0.066	0.101	0.026	0.176	-0.045	-0.041	**0.587**	-0.022	-0.060	**-0.275**
COR-RAMA	0.171	-0.167	-0.038	-0.189	-0.066	-0.097	**-0.229**	-0.149	-0.094	**0.227**
RAMA-PMA	0.011	-0.055	0.019	-0.035	-0.070	-0.083	0.091	-0.133	0.081	-0.059
COR-IMA	0.047	-0.014	-0.020	-0.055	0.052	-0.060	-0.190	-0.024	-0.059	0.092
MFO-ALV	**-0.227**	**-0.363**	-0.182	**-0.271**	-0.145	0.018	**0.225**	-0.033	**0.284**	0.097
MO-MP3	-0.002	0.118	0.059	0.108	0.041	0.021	-0.085	-0.079	0.102	**-0.213**
MP3-BDM1	0.056	0.102	0.063	0.036	0.044	-0.022	-0.050	-0.046	0.035	-0.117
BDM1-RAMA	**-0.267**	-0.098	-0.139	-0.032	0.011	0.108	-0.035	**0.265**	**-0.259**	0.139

CON-MALV	-0.152	-0.120	-0.059	-0.050	-0.072	-0.040	0.103	0.011	-0.111	0.026
MFO-CONM	-0.007	0.087	0.069	**0.234**	-0.017	-0.039	-0.037	0.104	**-0.528**	-0.117
RAMA-GH	-0.077	0.074	-0.052	0.008	0.141	0.057	-0.039	0.139	-0.107	-0.019
MP3-MEN	**-0.242**	**-0.263**	-0.070	**0.210**	**-0.309**	**0.220**	**-0.234**	-0.122	0.094	**-0.290**
MEN-GH	0.136	-0.023	-0.048	-0.097	0.082	**-0.225**	-0.120	0.054	0.080	**-0.213**
ALV-IMA	-0.035	-0.005	0.048	0.037	-0.112	-0.064	-0.093	0.017	-0.022	-0.085
ALV-RAMA	**0.741**	-0.187	-0.165	0.119	**-0.380**	**0.249**	0.178	0.041	-0.157	0.072
LDM1-BDM1	-0.041	-0.041	-0.061	-0.029	0.040	0.027	-0.064	0.014	-0.163	0.097
LDM1-GH	0.028	0.056	-0.053	0.008	0.140	0.049	-0.066	0.018	-0.160	-0.008
Percent variance explained (%)	10.995	7.776	6.984	6.453	5.341	5.002	4.371	3.913	3.564	3.270

Table S2.15. First 10 principal components for raw cranial data across Cercopithecidae. Highlighted values depict relatively large loadings (<-0.2 and >0.2).

	PC1	PC2	PC3	PC4	PC5	PC6	PC7	PC8	PC9	PC10
IS-PM	-0.041	0.004	0.014	0.005	0.023	0.005	-0.054	0.044	-0.061	0.022
IS-NSL	**-0.204**	-0.006	-0.135	**0.205**	**-0.294**	-0.128	-0.176	**0.202**	-0.041	0.096
IS-PNS	**-0.333**	-0.105	-0.137	0.126	-0.090	-0.068	**-0.256**	0.008	-0.041	0.042
PM-ZS	**-0.365**	-0.195	-0.170	-0.097	-0.062	-0.015	-0.136	0.046	0.060	0.150
PM-ZI	**-0.273**	-0.156	-0.166	0.049	-0.059	-0.014	**-0.255**	0.033	0.087	-0.015
PM-MT	-0.195	-0.006	-0.025	0.018	0.120	-0.031	**-0.397**	**0.229**	-0.081	0.064
NSL-NA	**-0.227**	**-0.269**	-0.011	**-0.539**	**0.236**	0.067	**0.314**	0.133	-0.059	0.044
NSL-ZS	-0.190	**-0.203**	-0.052	**-0.291**	**0.292**	0.166	-0.122	**-0.305**	0.110	-0.021
NSL-ZI	-0.165	-0.081	-0.011	-0.102	0.152	0.097	-0.114	-0.058	0.052	-0.043
NA-BR	-0.130	**0.495**	-0.167	0.035	0.021	**0.278**	-0.020	-0.082	0.140	**0.376**
NA-FM	-0.074	0.038	0.038	0.032	0.046	0.063	-0.027	0.000	-0.059	-0.013
NA-PNS	-0.166	0.027	0.062	-0.028	0.011	0.055	0.193	**0.223**	**-0.211**	0.040
BR-ZAF	-0.138	**0.387**	-0.075	0.004	0.152	**0.357**	0.111	0.093	**0.257**	0.089
BR-APET	-0.097	0.159	0.036	**0.227**	0.192	0.120	0.018	0.051	-0.080	0.044
ZAF-FM	-0.049	-0.085	**-0.205**	**0.225**	0.037	**0.382**	0.145	0.086	**-0.214**	-0.157
TS-MT	**-0.266**	-0.073	-0.113	0.154	**-0.329**	-0.008	**0.326**	**-0.383**	**0.206**	**-0.217**

ZAF-BA	-0.170	**0.213**	**0.275**	-0.086	-0.045	**-0.361**	-0.052	-0.025	0.107	-0.044
ZAF-EAM	-0.188	0.180	**0.250**	-0.096	-0.012	-0.195	0.055	-0.009	0.148	0.033
ZAF-ZYGO	-0.133	0.074	0.184	-0.054	0.012	**-0.239**	0.025	**-0.227**	-0.192	**0.269**
AS-EAM	-0.059	0.093	**0.207**	-0.095	-0.175	**0.312**	-0.184	-0.062	**-0.309**	**-0.261**
FM-ZS	-0.047	0.033	0.004	0.014	-0.089	-0.070	**0.211**	**0.220**	-0.158	0.058
FM-MT	**-0.231**	-0.025	-0.077	0.103	-0.150	0.021	**0.327**	0.087	-0.167	-0.003
ZS-ZI	-0.122	0.010	0.032	-0.046	-0.052	-0.029	0.148	0.184	-0.064	0.144
ZI-MT	-0.067	-0.001	-0.003	0.047	0.051	-0.025	-0.038	**0.218**	-0.085	-0.017
ZI-ZYGO	-0.147	0.043	0.077	0.065	-0.021	-0.036	0.136	**-0.358**	**-0.352**	**0.269**
NA-ORB	-0.053	-0.020	0.021	-0.080	0.019	0.007	0.165	0.163	-0.101	-0.069
MT-PNS	-0.125	-0.061	-0.088	0.111	-0.141	-0.012	0.125	-0.196	0.090	-0.080
PNS-APET	-0.117	0.001	0.015	-0.046	-0.047	0.042	0.064	-0.057	0.102	-0.160
APET-BA	-0.062	0.040	0.039	0.013	-0.001	-0.035	0.010	0.023	-0.024	-0.030
APET-TS	-0.030	0.028	0.019	0.026	-0.015	-0.012	0.044	-0.013	0.008	0.007
BA-EAM	-0.127	0.049	0.058	0.054	0.066	-0.002	0.013	-0.018	-0.097	-0.092
EAM-ZYGO	-0.114	0.060	0.035	0.005	-0.048	-0.045	0.090	**0.297**	**0.395**	**-0.352**
ORB-ZS	-0.014	-0.004	-0.027	0.049	0.092	0.128	-0.143	**-0.281**	0.032	-0.138
LD-AS	-0.099	0.091	**-0.210**	**0.232**	**0.522**	**-0.332**	0.157	-0.031	0.099	-0.028
BR-LD	-0.092	**-0.399**	**0.583**	**0.493**	**0.218**	0.191	0.058	0.044	0.188	0.164
OPI-LD	-0.099	0.176	-0.103	0.145	**0.328**	**-0.208**	-0.016	-0.067	**-0.295**	**-0.435**
ZAF-AS	**-0.216**	**0.239**	**0.389**	-0.151	-0.092	0.103	-0.069	0.001	0.045	**-0.203**
JP-AS	-0.102	0.087	0.132	0.018	0.048	0.085	-0.102	-0.017	**-0.209**	-0.190
BA-OPI	-0.027	0.011	0.034	-0.009	0.037	0.058	0.015	0.014	0.042	0.096
Percent variance explained (%)	45.512	6.888	5.458	4.455	3.933	3.182	3.056	2.708	2.453	2.262

Table S2.16. First 10 principal components for log-scale ratio cranial data across Cercopithecidae. Highlighted values depict relatively large loadings (<-0.2 and >0.2).

	PC1	PC2	PC3	PC4	PC5	PC6	PC7	PC8	PC9	PC10
IS-PM	0.039	-0.030	-0.176	0.053	-0.028	**-0.223**	-0.187	-0.049	0.007	-0.058
IS-NSL	-0.057	0.069	**0.204**	-0.114	-0.090	-0.136	-0.045	-0.016	0.039	**-0.305**

IS-PNS	-0.030	0.034	0.114	-0.056	-0.023	-0.053	-0.083	-0.035	0.095	-0.032
PM-ZS	-0.084	0.051	**0.306**	0.008	-0.006	-0.111	-0.134	-0.038	0.150	0.072
PM-ZI	-0.044	0.057	**0.215**	-0.026	-0.014	-0.048	-0.087	-0.032	0.050	0.032
PM-MT	-0.011	-0.017	-0.035	-0.010	-0.044	-0.129	-0.109	-0.023	0.086	0.040
NSL-NA	-0.146	0.027	0.144	**0.607**	0.024	0.000	-0.113	-0.006	-0.072	**0.215**
NSL-ZS	0.142	-0.121	**0.201**	**0.211**	0.032	-0.041	-0.184	-0.041	**0.202**	**0.270**
NSL-ZI	0.003	-0.017	0.024	0.039	0.007	-0.018	-0.055	-0.024	-0.022	0.141
NA-BR	0.008	-0.044	-0.098	-0.139	-0.070	0.032	-0.009	**0.211**	-0.055	0.048
NA-FM	0.040	-0.056	-0.082	0.023	0.023	0.052	0.047	0.013	-0.052	-0.007
NA-PNS	-0.004	-0.004	-0.025	0.128	-0.001	0.030	0.037	0.015	0.011	-0.085
BR-ZAF	0.017	-0.030	-0.106	-0.044	-0.089	0.034	0.072	0.169	-0.024	0.103
BR-APET	0.024	-0.031	-0.154	-0.077	-0.017	0.072	0.016	0.006	0.037	0.074
ZAF-FM	**0.414**	**0.810**	-0.130	0.006	0.094	0.031	-0.040	0.048	0.039	0.073
TS-MT	-0.082	0.121	**0.410**	-0.137	0.036	0.113	0.175	-0.002	-0.031	-0.125
ZAF-BA	-0.086	-0.175	-0.026	-0.063	-0.038	-0.047	-0.013	0.025	-0.016	-0.036
ZAF-EAM	-0.099	-0.136	0.028	-0.056	-0.036	-0.075	0.001	-0.028	-0.068	-0.001
ZAF-ZYGO	-0.110	-0.177	0.034	-0.074	0.134	0.155	**-0.275**	-0.106	-0.076	-0.126
AS-EAM	-0.003	-0.079	-0.117	-0.052	**0.541**	-0.170	0.073	**0.438**	0.105	0.050
FM-ZS	**-0.205**	0.116	**-0.234**	-0.037	-0.008	0.111	0.022	0.072	**-0.491**	**-0.288**
FM-MT	-0.018	0.078	0.055	-0.027	-0.017	-0.008	-0.021	-0.014	-0.082	-0.192
ZS-ZI	**-0.201**	0.103	-0.006	-0.118	0.034	-0.046	-0.020	-0.026	-0.126	**0.237**
ZI-MT	0.004	0.023	**-0.270**	0.045	-0.165	**-0.365**	-0.195	-0.161	**0.278**	**-0.458**
ZI-ZYGO	-0.022	-0.004	0.031	-0.091	**0.208**	**0.228**	**-0.287**	-0.056	-0.079	-0.140
NA-ORB	-0.119	0.043	-0.043	**0.617**	-0.033	**0.243**	**0.233**	0.117	0.054	**-0.283**
MT-PNS	-0.058	0.109	**0.382**	**-0.201**	0.028	**0.222**	0.164	-0.067	-0.033	-0.070
PNS-APET	-0.021	0.048	0.096	0.035	0.005	**-0.239**	-0.078	0.029	**-0.316**	0.096
APET-BA	-0.022	-0.015	-0.034	-0.067	-0.006	0.006	0.000	0.051	0.171	-0.013
APET-TS	-0.039	-0.076	-0.107	-0.076	0.005	**0.305**	**0.380**	0.016	**0.454**	-0.089
BA-EAM	-0.008	-0.018	-0.044	-0.059	0.020	0.028	0.015	0.009	0.066	0.051
EAM-ZYGO	-0.062	0.041	0.050	-0.033	**-0.353**	**-0.438**	**0.507**	-0.013	-0.049	0.134

ORB-ZS	**0.787**	**-0.357**	0.174	0.052	-0.033	0.007	0.091	0.030	**-0.204**	-0.177
LD-AS	0.041	-0.018	-0.136	-0.055	**-0.493**	**0.336**	-0.156	-0.124	-0.030	**0.205**
BR-LD	0.037	-0.028	-0.179	-0.020	**0.316**	0.014	0.171	**-0.725**	0.081	0.124
OPI-LD	0.022	-0.041	-0.116	-0.107	**-0.266**	0.186	-0.181	**0.246**	0.147	0.162
ZAF-AS	-0.057	-0.107	-0.035	-0.046	0.116	-0.098	0.065	0.104	-0.018	0.063
JP-AS	-0.018	-0.066	-0.100	-0.062	0.136	-0.007	0.011	0.133	0.094	0.130
BA-OPI	0.028	-0.082	**-0.216**	0.023	0.069	0.051	0.192	-0.148	**-0.324**	0.162
Percent variance explained (%)	15.019	12.576	8.786	6.294	5.018	4.657	4.323	4.092	3.750	3.552

Table S2.17. First 10 principal components for raw cranial data across Cercopithecinae. Highlighted values depict relatively large loadings (<-0.2 and >0.2).

	PC1	PC2	PC3	PC4	PC5	PC6	PC7	PC8	PC9	PC10
IS-PM	-0.041	-0.006	-0.017	0.005	0.024	-0.054	0.016	-0.051	-0.062	0.007
IS-NSL	**-0.203**	-0.005	0.150	**0.226**	**-0.276**	-0.115	0.183	**-0.223**	-0.041	0.110
IS-PNS	**-0.335**	0.097	0.150	0.135	-0.084	**-0.218**	0.138	0.041	-0.057	0.036
PM-ZS	**-0.370**	0.189	0.170	-0.091	-0.078	-0.115	0.078	-0.058	0.067	0.193
PM-ZI	**-0.275**	0.147	0.170	0.058	-0.052	**-0.251**	0.094	-0.023	0.095	-0.034
PM-MT	-0.196	-0.005	0.026	0.015	0.123	**-0.369**	0.184	**-0.208**	-0.065	0.010
NSL-NA	**-0.233**	**0.276**	-0.026	**-0.568**	0.187	**0.271**	-0.098	-0.127	-0.033	0.055
NSL-ZS	-0.195	**0.210**	0.040	**-0.315**	**0.262**	-0.163	-0.143	**0.289**	0.076	-0.002
NSL-ZI	-0.165	0.079	0.000	-0.118	0.159	-0.160	-0.062	0.065	0.053	-0.075
NA-BR	-0.125	**-0.497**	0.156	0.005	0.037	-0.103	**-0.253**	0.022	0.175	**0.361**
NA-FM	-0.073	-0.040	-0.042	0.027	0.049	-0.049	-0.057	-0.035	-0.069	-0.029
NA-PNS	-0.165	-0.032	-0.079	-0.038	0.002	0.177	-0.087	**-0.290**	**-0.207**	0.004
BR-ZAF	-0.135	**-0.387**	0.050	-0.039	0.150	-0.004	**-0.338**	-0.104	**0.277**	0.123
BR-APET	-0.096	-0.162	-0.033	0.192	**0.201**	-0.027	-0.113	-0.069	-0.066	0.065
ZAF-FM	-0.050	0.074	0.196	0.175	0.065	0.005	**-0.435**	-0.183	-0.179	**-0.201**
TS-MT	**-0.264**	0.074	0.128	0.183	**-0.309**	**0.302**	-0.171	**0.397**	0.166	-0.184
ZAF-BA	-0.164	**-0.204**	**-0.262**	-0.042	-0.057	0.071	**0.371**	0.108	0.079	-0.064
ZAF-EAM	-0.185	-0.177	**-0.248**	-0.065	-0.016	0.115	0.168	0.041	0.150	-0.011

ZAF-ZYGO	-0.132	-0.079	-0.174	-0.028	0.010	0.093	0.184	0.198	-0.196	**0.242**
AS-EAM	-0.056	-0.099	**-0.215**	-0.096	-0.193	**-0.276**	**-0.239**	0.037	**-0.305**	-0.200
FM-ZS	-0.045	-0.035	-0.013	0.011	-0.078	**0.216**	0.029	**-0.227**	-0.118	0.041
FM-MT	**-0.231**	0.024	0.074	0.098	-0.151	**0.317**	-0.133	-0.138	-0.142	-0.024
ZS-ZI	-0.123	-0.016	-0.044	-0.052	-0.049	0.146	0.019	-0.174	-0.029	0.159
ZI-MT	-0.068	0.002	0.005	0.045	0.052	-0.022	0.054	**-0.230**	-0.053	-0.077
ZI-ZYGO	-0.146	-0.048	-0.063	0.073	-0.013	0.145	-0.067	**0.296**	**-0.381**	**0.297**
NA-ORB	-0.052	0.019	-0.033	-0.082	0.008	0.151	-0.036	-0.185	-0.100	-0.074
MT-PNS	-0.125	0.062	0.100	0.122	-0.136	0.129	-0.067	**0.221**	0.074	-0.036
PNS-APET	-0.116	-0.002	-0.019	-0.037	-0.037	0.034	-0.080	0.038	0.099	-0.184
APET-BA	-0.061	-0.044	-0.040	0.013	-0.005	0.019	0.036	-0.016	-0.026	-0.041
APET-TS	-0.029	-0.029	-0.019	0.027	-0.011	0.043	0.001	0.022	0.005	0.008
BA-EAM	-0.126	-0.053	-0.058	0.052	0.073	0.013	-0.005	0.015	-0.096	-0.091
EAM-ZYGO	-0.113	-0.056	-0.045	0.013	-0.044	0.099	0.057	**-0.222**	**0.412**	**-0.414**
ORB-ZS	-0.013	0.003	0.033	0.047	0.085	-0.160	-0.137	**0.227**	-0.026	-0.165
LD-AS	-0.099	-0.086	**0.217**	0.199	**0.552**	**0.254**	**0.239**	0.065	0.079	-0.031
BR-LD	-0.092	**0.401**	**-0.572**	**0.491**	**0.247**	-0.018	-0.193	-0.062	0.187	0.182
OPI-LD	-0.099	-0.176	0.110	0.122	**0.324**	0.058	0.153	0.103	**-0.328**	**-0.378**
ZAF-AS	**-0.211**	**-0.237**	**-0.399**	-0.132	-0.111	-0.095	-0.055	0.035	0.045	-0.187
JP-AS	-0.101	-0.094	-0.133	0.018	0.049	-0.120	-0.052	0.010	**-0.204**	-0.153
BA-OPI	-0.026	-0.010	-0.038	-0.015	0.041	-0.010	-0.050	-0.035	0.056	0.076
Percent variance explained (%)	47.223	6.757	5.278	4.479	3.892	3.051	2.984	2.577	2.368	2.119

Table S2.18. First 10 principal components for log-scale ratio cranial data across Cercopithecinae. Highlighted values depict relatively large loadings (<-0.2 and >0.2).

	PC1	PC2	PC3	PC4	PC5	PC6	PC7	PC8	PC9	PC10
IS-PM	0.042	-0.038	-0.176	0.056	-0.062	**-0.246**	-0.169	0.073	-0.029	-0.094
IS-NSL	-0.056	0.078	**0.201**	-0.103	-0.096	-0.133	-0.031	0.001	0.077	**-0.290**
IS-PNS	-0.029	0.039	0.122	-0.053	-0.030	-0.059	-0.087	0.046	0.069	-0.033
PM-ZS	-0.089	0.072	**0.303**	0.020	-0.019	-0.117	-0.149	0.061	0.098	0.085

PM-ZI	-0.046	0.066	**0.213**	-0.025	-0.020	-0.056	-0.089	0.049	0.030	0.038
PM-MT	-0.013	-0.017	-0.026	-0.012	-0.053	-0.126	-0.117	0.035	0.052	0.052
NSL-NA	-0.137	0.035	0.133	**0.581**	-0.003	-0.002	-0.121	0.015	-0.122	**0.153**
NSL-ZS	0.123	-0.104	**0.208**	**0.224**	0.011	-0.070	**-0.228**	0.107	0.103	**0.310**
NSL-ZI	0.003	-0.019	0.022	0.044	-0.001	-0.027	-0.063	0.040	-0.060	0.136
NA-BR	0.005	-0.054	-0.095	-0.146	-0.036	0.034	-0.052	**-0.224**	-0.040	0.058
NA-FM	0.041	-0.064	-0.084	0.026	0.027	0.055	0.049	-0.015	-0.041	-0.013
NA-PNS	-0.002	-0.008	-0.025	0.141	-0.003	0.040	0.036	-0.029	0.035	-0.081
BR-ZAF	0.016	-0.039	-0.104	-0.054	-0.050	0.044	0.018	-0.179	-0.023	0.113
BR-APET	0.021	-0.043	-0.148	-0.087	-0.007	0.077	-0.002	0.002	0.029	0.075
ZAF-FM	**0.438**	**0.793**	-0.167	0.005	0.109	0.022	-0.044	-0.034	0.031	0.090
TS-MT	-0.076	0.143	**0.402**	-0.131	0.054	0.107	**0.183**	-0.026	0.026	-0.098
ZAF-BA	-0.088	-0.170	-0.020	-0.064	-0.035	-0.039	-0.002	-0.031	-0.013	-0.043
ZAF-EAM	-0.101	-0.122	0.033	-0.048	-0.045	-0.070	0.034	0.000	-0.067	0.000
ZAF-ZYGO	-0.118	-0.163	0.044	-0.060	0.102	0.133	**-0.210**	0.130	-0.080	-0.144
AS-EAM	-0.009	-0.094	-0.120	-0.033	**0.613**	-0.195	-0.025	**-0.318**	0.111	0.057
FM-ZS	**-0.205**	0.112	**-0.250**	-0.048	0.007	0.136	0.087	-0.119	**-0.411**	**-0.377**
FM-MT	-0.019	0.081	0.060	-0.021	-0.019	0.000	0.010	0.000	-0.045	**-0.214**
ZS-ZI	-0.185	0.093	-0.007	-0.102	0.030	-0.053	-0.022	0.010	-0.156	**0.168**
ZI-MT	-0.002	0.022	**-0.266**	0.039	**-0.224**	**-0.357**	-0.156	**0.179**	**0.340**	**-0.422**
ZI-ZYGO	-0.024	0.007	0.035	-0.080	**0.191**	**0.212**	**-0.249**	0.116	-0.102	-0.170
NA-ORB	-0.111	0.042	-0.061	**0.629**	-0.024	**0.280**	**0.225**	-0.154	0.141	**-0.205**
MT-PNS	-0.056	0.123	**0.384**	**-0.201**	0.045	**0.231**	**0.182**	0.054	-0.012	-0.088
PNS-APET	-0.020	0.060	0.071	0.040	-0.002	**-0.234**	-0.023	-0.021	**-0.338**	0.059
APET-BA	-0.022	-0.020	-0.030	-0.075	0.010	-0.004	-0.016	-0.055	**0.164**	-0.008
APET-TS	-0.033	-0.091	-0.088	-0.080	0.030	**0.289**	**0.246**	-0.059	**0.542**	0.064
BA-EAM	-0.008	-0.021	-0.042	-0.064	0.017	0.024	0.001	0.005	0.048	0.057
EAM-ZYGO	-0.058	0.043	0.045	-0.045	**-0.343**	**-0.402**	**0.535**	-0.152	-0.001	**0.172**
ORB-ZS	**0.783**	**-0.376**	**0.188**	0.050	-0.030	0.018	0.108	-0.073	-0.146	**-0.220**
LD-AS	0.041	-0.017	-0.131	-0.090	**-0.486**	**0.345**	-0.152	0.092	-0.085	**0.201**

BR-LD	0.032	-0.039	-0.171	-0.021	**0.217**	0.018	**0.289**	**0.758**	0.032	0.099
OPI-LD	0.020	-0.039	-0.109	-0.134	-0.196	**0.188**	**-0.243**	-0.198	0.099	0.149
ZAF-AS	-0.060	-0.105	-0.032	-0.035	0.133	-0.099	0.049	-0.090	-0.022	0.064
JP-AS	-0.021	-0.070	-0.096	-0.066	0.148	-0.011	-0.034	-0.084	0.068	0.137
BA-OPI	0.026	-0.094	**-0.215**	0.022	0.041	0.047	**0.233**	0.091	**-0.303**	**0.166**
Percent variance explained (%)	15.451	12.594	9.100	6.202	5.029	4.739	4.343	4.184	3.755	3.655

Table S2.19. First 10 principal components for raw cranial data across Colobinae. Highlighted values depict relatively large loadings (<-0.2 and >0.2).

	PC1	PC2	PC3	PC4	PC5	PC6	PC7	PC8	PC9	PC10
IS-PM	-0.024	0.001	0.007	-0.005	-0.007	-0.005	0.031	-0.007	-0.073	0.043
IS-NSL	**-0.224**	-0.076	0.142	**-0.218**	0.118	-0.027	0.009	-0.003	-0.179	-0.185
IS-PNS	**-0.251**	-0.086	0.174	-0.076	0.064	0.013	0.130	0.016	**-0.356**	**0.233**
PM-ZS	**-0.216**	-0.031	0.110	-0.183	0.051	-0.070	-0.182	0.052	-0.156	-0.022
PM-ZI	-0.182	-0.066	0.163	**-0.244**	0.058	-0.098	-0.056	0.066	-0.120	0.102
PM-MT	-0.131	-0.009	0.061	0.045	0.097	0.023	0.104	0.031	**-0.504**	-0.006
NSL-NA	-0.056	-0.012	0.062	-0.109	0.131	0.073	0.055	0.113	0.089	**0.274**
NSL-ZS	-0.062	0.077	-0.041	-0.037	0.005	**-0.267**	**-0.472**	0.117	-0.105	0.056
NSL-ZI	-0.137	-0.005	0.091	-0.149	0.029	-0.072	-0.051	0.104	0.001	0.115
NA-BR	**-0.238**	**-0.321**	**-0.270**	0.054	**-0.559**	0.042	-0.050	-0.003	-0.064	-0.064
NA-FM	-0.104	-0.002	0.022	-0.014	-0.019	-0.096	-0.074	0.059	0.039	0.122
NA-PNS	-0.168	-0.060	0.082	-0.004	0.001	-0.002	0.096	0.060	-0.067	0.125
BR-ZAF	**-0.209**	**-0.431**	-0.145	**0.348**	-0.090	0.175	-0.195	**0.210**	0.062	**0.202**
BR-APET	-0.130	-0.132	0.061	**0.376**	-0.099	-0.164	0.141	-0.007	0.094	-0.119
ZAF-FM	0.004	**-0.295**	**0.332**	-0.028	-0.168	-0.172	0.087	**-0.226**	0.031	**-0.248**
TS-MT	**-0.289**	-0.055	0.123	**-0.304**	-0.037	0.012	-0.121	0.042	**0.479**	-0.022
ZAF-BA	**-0.304**	**0.281**	**-0.267**	0.100	0.145	0.150	0.082	0.128	-0.023	0.074
ZAF-EAM	**-0.263**	**0.284**	**-0.229**	-0.007	-0.051	0.095	0.104	0.109	-0.009	-0.171
ZAF-ZYGO	-0.139	**0.393**	-0.151	-0.160	**-0.313**	**-0.238**	**0.237**	-0.037	0.031	-0.121
AS-EAM	-0.129	0.057	0.131	0.128	0.046	0.097	-0.198	**-0.523**	0.039	0.064

FM-ZS	-0.074	-0.072	0.064	-0.103	0.000	0.078	**0.283**	-0.042	0.077	0.073
FM-MT	-0.200	-0.125	0.111	-0.039	-0.077	-0.057	0.194	-0.109	-0.030	-0.138
ZS-ZI	-0.090	-0.052	0.106	-0.055	0.001	0.143	**0.269**	0.031	0.089	0.090
ZI-MT	-0.045	-0.006	-0.007	0.093	0.004	0.028	0.142	-0.083	**-0.298**	-0.157
ZI-ZYGO	-0.153	0.189	0.003	-0.029	**-0.321**	-0.167	0.070	-0.140	0.036	0.164
NA-ORB	-0.072	-0.035	0.044	-0.055	0.175	-0.010	-0.010	0.112	0.135	0.142
MT-PNS	-0.118	-0.051	0.074	-0.071	0.028	-0.017	-0.043	-0.049	0.120	0.045
PNS-APET	-0.143	0.039	-0.010	-0.177	0.053	0.007	-0.178	0.004	0.181	**-0.295**
APET-BA	-0.076	0.007	0.025	0.017	0.016	-0.025	0.053	0.055	0.037	0.059
APET-TS	-0.040	-0.029	0.023	0.010	-0.031	0.056	0.066	0.007	0.068	0.090
BA-EAM	-0.136	0.005	0.059	0.054	0.055	-0.031	-0.004	-0.173	0.096	0.117
EAM-ZYGO	-0.148	-0.125	-0.018	0.027	**0.270**	**0.242**	-0.004	0.143	0.052	**-0.589**
ORB-ZS	-0.029	0.048	-0.038	0.029	-0.059	**-0.299**	**-0.388**	0.045	**-0.206**	-0.130
LD-AS	-0.096	-0.152	-0.185	0.071	0.168	**-0.455**	0.191	**0.252**	0.129	0.055
BR-LD	-0.070	**0.327**	**0.608**	**0.473**	-0.104	-0.074	-0.015	**0.343**	0.099	-0.077
OPI-LD	-0.121	-0.085	**-0.211**	**0.222**	**0.408**	**-0.464**	0.141	**-0.239**	0.096	-0.037
ZAF-AS	**-0.339**	0.179	-0.082	**0.215**	0.074	**0.258**	-0.157	-0.165	0.020	0.028
JP-AS	-0.117	0.074	0.048	0.109	0.100	0.001	-0.086	**-0.411**	0.042	0.089
BA-OPI	-0.035	0.009	0.007	0.000	-0.141	-0.023	-0.005	0.017	0.017	0.008
Percent variance explained (%)	26.475	8.924	7.881	6.405	5.374	4.772	4.393	3.482	3.451	2.752

Table S2.20. First 10 principal components for log-scale ratio cranial data across Colobinae. Highlighted values depict relatively large loadings (<-0.2 and >0.2).

	PC1	PC2	PC3	PC4	PC5	PC6	PC7	PC8	PC9	PC10
IS-PM	0.009	0.005	0.032	0.168	0.036	0.020	0.071	0.114	-0.048	0.110
IS-NSL	-0.048	-0.007	0.083	**-0.290**	-0.103	0.023	-0.002	0.027	-0.199	0.195
IS-PNS	-0.044	-0.004	0.031	-0.027	-0.003	-0.050	-0.003	0.071	0.001	0.057
PM-ZS	0.012	-0.033	-0.058	**-0.251**	0.060	-0.065	-0.032	0.050	-0.058	-0.052
PM-ZI	-0.037	0.006	-0.056	-0.190	0.061	-0.036	0.063	0.005	-0.013	0.187
PM-MT	0.008	0.006	0.019	0.110	-0.038	-0.014	0.001	0.187	-0.096	0.059

NSL-NA	-0.171	**-0.233**	**-0.729**	0.186	**0.372**	-0.163	0.145	0.102	0.087	-0.139
NSL-ZS	**0.325**	0.092	-0.160	-0.091	-0.027	0.143	-0.126	-0.130	0.052	0.110
NSL-ZI	-0.003	-0.008	0.003	-0.072	0.016	0.003	0.025	-0.017	0.050	**0.229**
NA-BR	0.011	0.031	0.109	0.028	-0.054	-0.035	0.084	-0.083	-0.163	-0.055
NA-FM	0.038	0.001	0.029	0.013	-0.027	0.037	-0.022	-0.058	0.030	0.141
NA-PNS	-0.026	-0.002	-0.026	0.042	-0.005	0.000	-0.014	-0.002	-0.032	0.076
BR-ZAF	-0.007	0.028	-0.014	0.068	**-0.216**	-0.128	-0.044	-0.009	-0.067	0.053
BR-APET	0.005	0.059	0.065	0.138	-0.081	0.019	-0.017	0.048	0.066	0.022
ZAF-FM	**-0.397**	**0.774**	-0.036	-0.012	0.103	0.147	0.106	-0.046	-0.011	**-0.207**
TS-MT	-0.091	-0.079	0.004	**-0.420**	0.065	-0.088	-0.039	**-0.253**	0.017	-0.151
ZAF-BA	0.097	-0.194	0.084	0.005	-0.059	0.014	-0.002	0.119	0.036	-0.053
ZAF-EAM	0.104	**-0.213**	0.131	-0.014	0.029	0.031	0.032	0.062	-0.096	-0.137
ZAF-ZYGO	0.165	**-0.200**	**0.234**	0.033	**0.381**	**0.308**	**0.265**	-0.144	-0.023	**-0.241**
AS-EAM	0.024	0.046	0.071	-0.021	0.023	0.046	**-0.325**	**0.203**	0.146	-0.111
FM-ZS	-0.197	-0.058	0.053	0.078	0.030	-0.016	0.194	-0.011	-0.173	**0.280**
FM-MT	-0.052	0.056	0.038	0.000	0.008	-0.010	0.086	0.040	-0.111	0.006
ZS-ZI	**-0.362**	-0.140	**0.210**	-0.047	0.134	**-0.264**	0.199	0.179	**0.231**	**0.331**
ZI-MT	0.026	0.064	0.031	**0.301**	-0.052	0.032	0.009	**0.291**	**-0.339**	**-0.234**
ZI-ZYGO	0.063	-0.027	0.178	0.010	**0.259**	**0.212**	0.076	-0.092	0.036	-0.190
NA-ORB	-0.113	-0.119	**-0.400**	-0.007	**-0.204**	**0.509**	**-0.256**	**-0.218**	-0.095	0.185
MT-PNS	-0.085	0.003	0.017	**-0.343**	-0.019	-0.083	-0.069	-0.133	**0.223**	-0.096
PNS-APET	0.034	-0.041	-0.071	**-0.309**	0.015	0.104	0.064	0.169	-0.188	-0.103
APET-BA	-0.027	-0.005	0.021	0.047	-0.044	0.088	0.049	0.000	**0.342**	-0.135
APET-TS	-0.108	-0.070	0.094	**0.285**	0.003	**-0.436**	**-0.283**	**-0.559**	-0.010	-0.168
BA-EAM	-0.010	-0.003	0.032	0.031	-0.021	-0.007	-0.106	-0.027	0.128	-0.131
EAM-ZYGO	-0.078	-0.053	-0.081	-0.094	**-0.321**	**-0.241**	-0.127	0.192	**-0.250**	**-0.260**
ORB-ZS	**0.640**	**0.353**	-0.184	-0.068	0.131	**-0.352**	0.136	0.009	0.035	0.132
LD-AS	0.048	-0.003	-0.031	0.156	**-0.323**	0.066	**0.374**	**-0.275**	0.103	0.079
BR-LD	0.024	0.099	0.097	0.189	**0.201**	0.119	**-0.408**	0.174	**0.283**	**0.260**
OPI-LD	0.077	-0.029	-0.026	0.123	**-0.437**	0.072	**0.300**	0.039	**0.338**	-0.110

ZAF-AS	0.059	-0.098	0.089	-0.008	-0.049	-0.017	-0.135	0.118	0.024	-0.050
JP-AS	0.038	-0.018	0.038	0.073	-0.009	0.003	-0.200	0.095	0.118	-0.113
BA-OPI	0.048	0.014	0.080	0.181	0.164	0.011	-0.068	**-0.238**	**-0.374**	**0.223**
Percent variance explained (%)	14.355	12.005	7.914	6.813	6.304	5.305	4.714	4.430	3.815	3.536

Table S2.21. First 10 principal components for raw cranial data across Papionini. Highlighted values depict relatively large loadings (<-0.2 and >0.2).

	PC1	PC2	PC3	PC4	PC5	PC6	PC7	PC8	PC9	PC10
IS-PM	-0.043	0.013	-0.029	0.013	0.017	0.069	-0.012	-0.039	-0.098	-0.044
IS-NSL	-0.194	0.054	**0.231**	0.195	**-0.227**	**0.214**	**-0.225**	-0.116	0.022	-0.130
IS-PNS	**-0.341**	-0.068	**0.205**	0.115	-0.045	**0.238**	0.074	0.076	-0.116	-0.094
PM-ZS	**-0.383**	-0.154	0.148	-0.126	-0.114	0.136	-0.084	0.008	0.195	**-0.210**
PM-ZI	**-0.280**	-0.119	0.195	0.010	-0.019	**0.294**	0.069	-0.008	0.151	0.158
PM-MT	-0.195	0.028	-0.007	-0.003	0.141	**0.462**	-0.034	-0.154	-0.064	-0.047
NSL-NA	**-0.255**	**-0.295**	**-0.210**	**-0.543**	0.061	**-0.243**	-0.124	-0.077	0.025	-0.065
NSL-ZS	**-0.215**	**-0.227**	-0.085	**-0.337**	0.188	-0.003	**0.294**	0.142	-0.034	0.039
NSL-ZI	-0.166	-0.090	-0.075	-0.130	0.163	0.119	**0.207**	0.016	-0.025	0.121
NA-BR	-0.108	**0.515**	0.068	-0.093	0.035	-0.039	**0.222**	0.003	**0.310**	-0.175
NA-FM	-0.068	0.052	-0.064	0.046	0.044	0.015	0.021	-0.081	-0.087	0.042
NA-PNS	-0.159	0.046	-0.131	0.011	-0.053	-0.162	**-0.256**	**-0.313**	-0.125	0.073
BR-ZAF	-0.128	**0.408**	-0.060	-0.102	0.109	-0.136	**0.202**	-0.106	**0.332**	-0.085
BR-APET	-0.086	0.181	-0.023	0.121	0.185	-0.023	0.036	-0.091	0.076	-0.085
ZAF-FM	-0.049	-0.030	0.184	0.067	0.114	-0.159	**0.276**	**-0.533**	-0.110	0.016
TS-MT	**-0.260**	-0.062	**0.250**	0.183	**-0.227**	**-0.388**	**0.200**	**0.278**	0.002	0.200
ZAF-BA	-0.149	0.173	**-0.234**	0.069	-0.072	0.086	**-0.254**	**0.346**	-0.060	**0.235**
ZAF-EAM	-0.175	0.152	**-0.233**	0.031	-0.017	-0.031	-0.122	0.147	0.033	0.090
ZAF-ZYGO	-0.128	0.060	-0.133	0.043	0.011	-0.023	-0.092	0.161	-0.190	-0.170
AS-EAM	-0.047	0.073	**-0.248**	0.030	**-0.261**	0.112	**0.306**	-0.170	**-0.216**	0.005
FM-ZS	-0.043	0.032	-0.010	0.002	-0.055	-0.111	-0.196	-0.156	0.006	-0.065
FM-MT	**-0.234**	0.003	0.123	0.091	-0.157	**-0.332**	-0.143	**-0.228**	-0.033	0.109

ZS-ZI	-0.126	0.021	-0.079	-0.031	-0.084	-0.094	-0.183	-0.047	0.075	**-0.345**
ZI-MT	-0.072	0.009	0.008	0.029	0.064	0.106	-0.140	**-0.216**	0.063	**0.259**
ZI-ZYGO	-0.139	0.043	-0.029	0.103	0.012	-0.169	0.073	0.127	**-0.406**	**-0.437**
NA-ORB	-0.048	-0.011	-0.070	-0.056	-0.006	-0.105	-0.168	-0.182	-0.091	0.127
MT-PNS	-0.127	-0.057	0.188	0.113	-0.111	**-0.208**	0.133	0.175	0.034	0.033
PNS-APET	-0.109	0.004	-0.037	-0.034	-0.018	-0.016	0.082	-0.014	-0.043	0.153
APET-BA	-0.055	0.044	-0.042	0.022	-0.004	-0.001	-0.054	0.023	-0.033	0.049
APET-TS	-0.025	0.025	-0.010	0.035	-0.006	-0.049	-0.036	0.032	0.024	-0.001
BA-EAM	-0.120	0.057	-0.063	0.060	0.088	-0.015	0.002	-0.017	-0.130	0.018
EAM-ZYGO	-0.107	0.061	-0.033	0.020	-0.011	-0.037	-0.116	-0.032	**0.229**	**0.392**
ORB-ZS	-0.011	0.012	0.038	0.056	0.066	-0.002	**0.243**	-0.008	**-0.231**	**0.255**
LD-AS	-0.105	0.110	**0.222**	0.031	**0.625**	-0.120	**-0.221**	0.167	-0.024	0.022
BR-LD	-0.092	**-0.409**	**-0.381**	**0.604**	**0.289**	-0.084	0.088	-0.053	**0.328**	-0.123
OPI-LD	-0.096	0.176	0.101	0.047	**0.306**	-0.018	-0.077	-0.002	**-0.358**	0.110
ZAF-AS	-0.196	0.191	**-0.417**	0.056	-0.149	0.035	0.125	0.030	-0.017	0.101
JP-AS	-0.097	0.085	-0.138	0.078	0.035	0.062	0.082	-0.083	-0.160	-0.006
BA-OPI	-0.026	0.008	-0.053	-0.009	0.041	0.001	0.019	-0.006	0.038	-0.055
Percent variance explained (%)	50.439	6.667	5.021	4.530	3.765	3.042	2.738	2.399	2.053	1.942

Table S2.22. First 10 principal components for log-scale ratio cranial data across Papionini. Highlighted values depict relatively large loadings (<-0.2 and >0.2).

	PC1	PC2	PC3	PC4	PC5	PC6	PC7	PC8	PC9	PC10
IS-PM	0.025	-0.067	0.140	0.090	-0.008	**-0.276**	0.057	-0.067	0.194	-0.065
IS-NSL	-0.056	0.071	-0.193	-0.033	0.072	-0.148	-0.116	0.198	**0.235**	-0.077
IS-PNS	-0.035	0.047	-0.132	-0.019	0.016	-0.110	0.026	0.018	0.049	-0.061
PM-ZS	-0.111	0.074	**-0.277**	0.059	-0.002	-0.150	0.018	-0.042	-0.083	-0.014
PM-ZI	-0.058	0.065	**-0.206**	0.025	0.019	-0.113	0.028	-0.003	-0.005	-0.050
PM-MT	-0.020	-0.023	0.037	0.022	0.027	-0.171	0.024	-0.040	-0.006	-0.006
NSL-NA	-0.136	0.014	-0.129	**0.498**	-0.044	0.047	0.074	-0.195	-0.171	0.011
NSL-ZS	0.062	-0.037	**-0.205**	**0.258**	-0.041	-0.154	0.199	**-0.302**	**-0.303**	-0.010

NSL-ZI	-0.006	-0.018	-0.017	0.065	-0.020	-0.058	0.092	-0.135	-0.046	-0.003
NA-BR	0.008	-0.071	0.088	-0.170	0.076	-0.033	**-0.250**	-0.102	-0.060	0.068
NA-FM	0.040	-0.077	0.091	-0.014	-0.024	0.083	-0.013	-0.002	0.058	0.003
NA-PNS	-0.005	-0.027	0.030	0.140	-0.006	0.090	-0.044	0.067	0.017	-0.030
BR-ZAF	0.014	-0.039	0.097	-0.087	0.055	0.013	-0.181	-0.105	-0.055	0.112
BR-APET	0.013	-0.059	0.132	-0.130	0.044	0.039	-0.010	-0.025	-0.025	-0.031
ZAF-FM	**0.296**	**0.841**	**0.285**	-0.001	-0.110	0.004	-0.001	-0.098	-0.122	0.017
TS-MT	-0.059	0.164	**-0.389**	-0.119	-0.018	0.117	-0.026	0.126	-0.006	0.079
ZAF-BA	-0.053	-0.185	0.008	-0.049	0.025	-0.041	-0.051	0.030	0.024	-0.006
ZAF-EAM	-0.053	-0.097	-0.027	-0.003	0.011	-0.047	-0.019	-0.024	0.015	0.125
ZAF-ZYGO	-0.069	-0.105	-0.051	-0.050	-0.019	0.020	0.058	-0.129	0.074	-0.195
AS-EAM	-0.002	-0.119	0.125	-0.090	**-0.616**	-0.060	**-0.284**	0.004	-0.088	-0.165
FM-ZS	-0.178	0.069	**0.217**	-0.050	0.018	0.167	-0.161	0.047	**0.338**	0.010
FM-MT	-0.020	0.090	-0.081	0.004	0.014	0.004	-0.049	0.145	0.075	-0.071
ZS-ZI	-0.196	0.029	-0.024	-0.101	-0.068	-0.016	0.011	-0.128	0.022	0.100
ZI-MT	-0.048	0.001	**0.261**	0.143	0.152	**-0.447**	0.125	**0.538**	-0.084	**-0.270**
ZI-ZYGO	-0.013	0.013	-0.041	-0.146	-0.068	0.094	0.061	**-0.215**	0.140	**-0.245**
NA-ORB	-0.100	-0.004	0.123	**0.590**	0.037	**0.498**	-0.186	0.166	0.130	-0.123
MT-PNS	-0.047	0.179	**-0.437**	**-0.236**	-0.014	**0.247**	0.015	**0.218**	0.093	-0.089
PNS-APET	-0.015	0.045	-0.031	0.106	-0.073	**-0.205**	-0.026	-0.165	**0.240**	**0.245**
APET-BA	-0.017	-0.059	0.043	-0.087	0.009	0.006	-0.033	0.052	**-0.249**	-0.114
APET-TS	-0.028	-0.110	0.079	-0.153	0.072	**0.301**	0.035	0.186	**-0.606**	0.052
BA-EAM	-0.005	-0.033	0.042	-0.087	-0.001	0.013	0.031	-0.015	-0.074	-0.020
EAM-ZYGO	-0.039	0.023	-0.006	0.047	0.108	-0.125	-0.098	**0.294**	-0.072	**0.661**
ORB-ZS	**0.873**	**-0.239**	-0.177	0.085	0.018	0.046	-0.078	0.100	0.102	-0.004
LD-AS	0.030	0.011	0.094	-0.089	**0.606**	0.095	0.151	**-0.238**	0.055	-0.047
BR-LD	0.022	-0.061	0.140	-0.101	**-0.235**	0.177	**0.762**	0.175	0.150	0.045
OPI-LD	0.013	-0.017	0.091	-0.128	**0.272**	-0.011	-0.147	-0.115	-0.040	**-0.239**
ZAF-AS	-0.031	-0.097	0.035	-0.032	-0.146	-0.028	-0.064	-0.026	-0.009	0.087
JP-AS	-0.015	-0.067	0.087	-0.109	-0.102	-0.007	-0.034	-0.055	-0.059	-0.032

BA-OPI	0.022	-0.124	0.180	-0.050	-0.039	0.136	0.101	-0.139	0.152	**0.351**
Percent variance explained (%)	17.781	12.535	9.835	5.995	5.500	5.078	4.399	4.083	3.362	3.236

Table S2.23. First 10 principal components for raw cranial data across Cercopithecini. Highlighted values depict relatively large loadings (<-0.2 and >0.2).

	PC1	PC2	PC3	PC4	PC5	PC6	PC7	PC8	PC9	PC10
IS-PM	-0.031	-0.024	-0.001	0.028	-0.009	0.002	0.018	-0.011	-0.002	0.093
IS-NSL	**-0.229**	**-0.208**	-0.029	-0.133	-0.019	-0.064	0.016	**0.246**	**-0.216**	0.046
IS-PNS	**-0.297**	-0.160	-0.057	-0.033	0.129	-0.027	0.100	0.157	-0.192	**0.425**
PM-ZS	**-0.294**	**-0.226**	-0.037	-0.118	0.171	0.058	0.090	0.094	-0.010	-0.108
PM-ZI	**-0.243**	**-0.221**	-0.041	-0.086	0.097	-0.020	0.094	0.123	-0.134	-0.076
PM-MT	-0.194	-0.089	-0.032	0.044	0.111	0.017	0.157	0.122	-0.084	0.126
NSL-NA	-0.124	-0.045	0.051	**-0.202**	0.163	-0.074	0.011	**-0.470**	**0.421**	0.196
NSL-ZS	-0.097	-0.086	-0.057	0.056	**0.295**	**0.276**	**0.223**	0.058	**0.288**	-0.161
NSL-ZI	-0.156	-0.092	-0.047	-0.016	0.042	0.025	0.129	0.038	0.101	-0.002
NA-BR	-0.198	0.130	**-0.385**	0.149	**-0.282**	**0.278**	**-0.226**	**0.300**	0.057	-0.001
NA-FM	-0.092	-0.061	-0.020	0.036	0.044	0.071	0.025	-0.005	0.095	0.022
NA-PNS	-0.185	-0.081	-0.022	0.010	0.047	0.020	-0.062	-0.171	**0.223**	**0.337**
BR-ZAF	-0.160	0.061	**-0.279**	**0.249**	**-0.412**	**0.210**	0.020	-0.071	**0.267**	0.076
BR-APET	-0.135	-0.041	-0.009	**0.403**	-0.080	-0.008	-0.056	-0.074	-0.095	0.010
ZAF-FM	-0.046	**-0.360**	-0.117	0.069	-0.101	0.027	-0.152	**-0.222**	-0.097	-0.032
TS-MT	**-0.272**	-0.112	0.009	**-0.245**	-0.121	-0.085	-0.154	-0.055	-0.023	**-0.394**
ZAF-BA	**-0.230**	**0.358**	0.137	-0.018	0.057	-0.158	0.099	0.150	0.040	0.016
ZAF-EAM	**-0.226**	**0.346**	0.161	-0.050	-0.049	-0.108	0.034	0.133	0.136	-0.006
ZAF-ZYGO	-0.150	**0.298**	0.163	0.003	**0.297**	-0.019	**-0.281**	**0.210**	0.059	-0.013
AS-EAM	-0.096	0.112	0.014	-0.012	0.046	**0.365**	0.065	**-0.343**	**-0.375**	-0.013
FM-ZS	-0.055	0.034	0.024	-0.091	-0.153	**-0.211**	**-0.264**	-0.085	-0.137	0.176
FM-MT	**-0.208**	-0.091	0.009	-0.052	-0.079	-0.103	**-0.225**	-0.085	0.024	0.068
ZS-ZI	-0.105	-0.004	0.005	-0.045	-0.123	-0.141	-0.046	-0.071	-0.046	0.093
ZI-MT	-0.047	-0.021	0.017	0.049	-0.015	-0.005	-0.087	0.008	0.018	0.088

ZI-ZYGO	-0.175	0.056	0.031	0.012	**0.205**	0.117	**-0.525**	-0.043	0.070	-0.093
NA-ORB	-0.065	-0.062	0.016	-0.088	0.024	-0.010	-0.045	-0.196	0.198	0.098
MT-PNS	-0.109	-0.046	-0.001	-0.054	-0.028	-0.060	-0.024	0.032	-0.032	-0.053
PNS-APET	-0.143	-0.056	0.028	-0.124	-0.138	-0.064	-0.030	-0.116	0.095	**-0.548**
APET-BA	-0.088	0.002	0.010	0.004	0.003	-0.033	0.011	-0.018	-0.033	0.049
APET-TS	-0.045	0.020	-0.010	0.016	0.006	0.017	-0.002	-0.018	-0.027	-0.024
BA-EAM	-0.147	-0.003	0.010	0.051	0.006	-0.044	-0.001	-0.080	-0.057	-0.054
EAM-ZYGO	-0.135	0.017	0.070	-0.121	**-0.391**	**-0.247**	**0.420**	-0.025	0.058	-0.021
ORB-ZS	-0.016	-0.067	-0.053	0.108	0.195	**0.214**	**0.206**	0.091	**0.224**	-0.134
LD-AS	-0.068	-0.003	-0.179	**0.388**	0.086	**-0.470**	0.006	0.049	**0.205**	-0.072
BR-LD	-0.091	**-0.272**	**0.747**	**0.452**	-0.156	0.103	-0.034	0.055	0.044	-0.021
OPI-LD	-0.107	0.125	**-0.227**	**0.397**	**0.274**	**-0.309**	0.116	**-0.289**	**-0.240**	-0.162
ZAF-AS	**-0.273**	**0.391**	0.134	-0.045	-0.119	**0.211**	0.189	-0.198	-0.097	0.019
JP-AS	-0.116	0.108	0.008	0.108	0.068	0.149	0.048	**-0.218**	**-0.224**	-0.047
BA-OPI	-0.026	-0.014	0.014	0.020	-0.109	0.059	-0.015	0.007	0.094	0.028
Percent variance explained (%)	38.616	7.162	6.562	5.071	4.160	3.998	3.600	3.215	2.972	2.841

Table S2.24. First 10 principal components for log-scale ratio cranial data across Cercopithecini. Highlighted values depict relatively large loadings (<-0.2 and >0.2).

	PC1	PC2	PC3	PC4	PC5	PC6	PC7	PC8	PC9	PC10
IS-PM	0.063	-0.067	**0.211**	-0.040	-0.111	0.092	0.094	0.048	**0.275**	**0.344**
IS-NSL	0.010	0.119	-0.183	0.163	0.071	0.135	-0.077	-0.152	0.134	**0.431**
IS-PNS	0.001	0.039	-0.079	0.058	0.060	-0.002	0.072	-0.054	0.149	0.194
PM-ZS	0.000	0.081	**-0.338**	0.031	0.081	0.041	0.016	-0.012	0.093	0.112
PM-ZI	0.020	0.081	-0.198	0.066	0.070	0.025	0.031	-0.019	-0.060	0.162
PM-MT	-0.006	-0.022	0.001	0.037	0.001	0.014	0.044	-0.009	0.095	0.105
NSL-NA	-0.066	0.130	-0.171	**-0.651**	**-0.230**	0.033	**0.284**	**0.230**	0.045	**-0.226**
NSL-ZS	0.070	**-0.319**	**-0.262**	-0.119	-0.006	-0.024	0.004	-0.019	0.006	0.057
NSL-ZI	0.005	-0.028	-0.030	0.006	-0.040	-0.001	0.074	0.005	-0.127	0.161
NA-BR	-0.011	-0.043	0.093	0.090	0.050	-0.074	0.048	0.002	-0.118	0.068

NA-FM	0.013	-0.076	0.045	-0.042	-0.021	0.015	-0.021	-0.049	-0.102	0.128
NA-PNS	0.016	-0.002	0.006	-0.122	-0.022	-0.005	0.016	-0.053	0.047	0.055
BR-ZAF	-0.001	-0.057	0.099	0.016	-0.057	-0.137	-0.002	-0.076	-0.116	0.068
BR-APET	0.018	-0.064	0.151	0.024	0.015	-0.053	-0.020	-0.066	-0.049	-0.020
ZAF-FM	**0.831**	**0.255**	0.084	-0.019	0.132	-0.043	0.014	0.080	0.033	-0.176
TS-MT	-0.019	**0.222**	**-0.359**	0.144	0.117	-0.038	-0.164	-0.058	-0.150	-0.168
ZAF-BA	-0.182	-0.045	0.023	0.069	-0.012	0.018	0.050	0.006	0.066	-0.047
ZAF-EAM	**-0.229**	-0.001	-0.023	0.098	-0.048	0.089	0.034	0.043	0.084	-0.167
ZAF-ZYGO	**-0.303**	-0.079	0.003	-0.022	**0.293**	**0.248**	0.053	0.021	0.064	**-0.219**
AS-EAM	-0.045	-0.073	0.076	-0.037	0.178	**-0.377**	**-0.247**	**0.554**	0.031	0.154
FM-ZS	-0.121	**0.284**	**0.367**	-0.032	0.045	0.177	0.118	0.054	**-0.236**	**0.207**
FM-MT	0.020	0.080	0.010	0.017	0.040	0.116	0.028	0.004	0.024	0.003
ZS-ZI	-0.030	**0.221**	0.085	0.101	-0.041	-0.008	**0.223**	0.155	-0.072	-0.028
ZI-MT	0.061	-0.057	**0.239**	-0.005	-0.027	**0.240**	-0.065	-0.062	**0.481**	-0.100
ZI-ZYGO	-0.038	0.018	-0.009	-0.096	**0.407**	**0.215**	-0.027	0.006	0.032	**-0.201**
NA-ORB	-0.039	0.128	-0.097	**-0.535**	-0.128	-0.140	**-0.250**	**-0.356**	-0.001	0.186
MT-PNS	-0.022	0.136	**-0.221**	0.147	0.087	0.001	-0.002	-0.124	-0.198	-0.028
PNS-APET	0.015	0.113	-0.122	0.059	-0.087	0.196	-0.044	**0.217**	**-0.262**	-0.152
APET-BA	-0.001	0.036	-0.002	0.069	0.044	-0.068	0.101	0.019	**0.214**	0.032
APET-TS	-0.058	-0.062	0.055	0.021	0.070	**-0.457**	**-0.272**	**-0.290**	0.087	**-0.240**
BA-EAM	-0.010	-0.002	0.031	0.038	0.021	-0.058	-0.008	0.003	0.012	-0.054
EAM-ZYGO	-0.036	0.163	-0.107	**0.334**	**-0.675**	-0.096	0.000	0.044	0.188	-0.175
ORB-ZS	**0.243**	**-0.671**	**-0.212**	0.047	-0.105	0.169	0.103	0.091	-0.119	-0.009
LD-AS	0.014	-0.090	0.188	0.033	-0.044	-0.034	**0.318**	**-0.400**	**-0.213**	-0.129
BR-LD	0.035	-0.061	0.180	-0.002	-0.066	**0.302**	**-0.472**	-0.106	0.072	**-0.228**
OPI-LD	-0.017	-0.101	0.135	0.054	0.120	**-0.317**	**0.382**	-0.126	-0.016	-0.123
ZAF-AS	-0.146	-0.031	0.022	0.050	-0.022	-0.101	-0.067	**0.210**	0.034	-0.028
JP-AS	-0.067	-0.064	0.084	-0.010	0.067	**-0.216**	-0.080	0.186	0.022	-0.035
BA-OPI	0.009	-0.086	**0.226**	-0.040	**-0.227**	0.122	**-0.288**	0.054	**-0.452**	0.084
Percent variance explained (%)	14.511	12.049	8.466	6.962	6.033	4.484	4.282	4.094	3.859	3.607

Table S2.25. First 10 principal components for raw cranial data across Asian colobines. Highlighted values depict relatively large loadings (<-0.2 and >0.2).

	PC1	PC2	PC3	PC4	PC5	PC6	PC7	PC8	PC9	PC10
IS-PM	-0.024	0.015	0.012	0.031	0.040	-0.004	-0.020	0.060	-0.046	-0.053
IS-NSL	**-0.204**	0.026	-0.056	-0.012	0.040	-0.063	0.115	0.068	-0.040	0.144
IS-PNS	**-0.269**	-0.016	0.062	**-0.212**	0.079	-0.154	**0.209**	0.069	**-0.386**	0.034
PM-ZS	**-0.253**	-0.057	0.047	-0.089	0.087	0.040	-0.041	0.007	-0.026	-0.007
PM-ZI	**-0.279**	-0.133	-0.101	-0.080	0.191	-0.067	-0.049	0.109	-0.086	0.070
PM-MT	-0.132	0.056	0.049	-0.071	0.050	**-0.207**	0.118	0.182	-0.106	-0.065
NSL-NA	-0.113	-0.055	-0.044	0.195	**0.228**	**0.269**	-0.163	**-0.202**	-0.173	**-0.335**
NSL-ZS	-0.009	0.026	0.021	**-0.331**	0.175	-0.018	**-0.357**	**-0.388**	0.045	**-0.231**
NSL-ZI	-0.151	-0.018	-0.163	-0.104	**0.211**	0.051	-0.185	-0.057	-0.106	-0.195
NA-BR	**-0.221**	-0.039	**0.422**	-0.157	0.148	0.128	0.055	-0.122	**0.345**	0.115
NA-FM	-0.126	0.017	-0.021	-0.079	-0.005	-0.034	-0.053	-0.100	-0.048	-0.028
NA-PNS	**-0.223**	-0.031	0.006	-0.039	0.133	-0.028	0.055	-0.020	0.088	-0.026
BR-ZAF	**-0.312**	**-0.251**	**0.347**	0.040	**-0.214**	-0.008	**0.286**	**-0.257**	0.056	-0.194
BR-APET	**-0.226**	-0.052	-0.040	-0.148	-0.056	**-0.267**	0.125	-0.019	**0.220**	0.018
ZAF-FM	-0.145	**-0.253**	**-0.229**	-0.101	-0.060	0.027	-0.080	**0.272**	0.144	0.021
TS-MT	**-0.318**	-0.050	-0.131	0.104	-0.129	**0.262**	-0.172	-0.062	-0.093	**0.413**
ZAF-BA	-0.100	**0.384**	0.035	**0.217**	-0.005	-0.177	0.141	-0.174	**0.243**	-0.089
ZAF-EAM	-0.061	**0.404**	0.011	0.105	0.090	0.042	0.113	-0.131	0.056	0.063
ZAF-ZYGO	-0.072	**0.498**	-0.083	0.035	**0.329**	0.062	-0.008	0.041	-0.026	0.171
AS-EAM	-0.142	0.137	0.061	-0.196	**-0.352**	0.059	-0.181	0.186	0.001	**-0.212**
FM-ZS	-0.081	-0.068	-0.090	**0.302**	0.064	0.113	0.083	0.015	0.014	-0.050
FM-MT	**-0.278**	-0.088	-0.174	0.197	0.086	-0.026	-0.076	0.137	**0.312**	-0.098
ZS-ZI	-0.131	-0.003	-0.058	0.149	0.005	0.143	0.140	**0.232**	-0.014	**-0.220**
ZI-MT	-0.096	0.095	-0.086	-0.026	0.019	-0.117	0.070	0.190	0.060	0.156
ZI-ZYGO	-0.117	**0.238**	-0.056	-0.113	0.140	0.164	0.071	0.172	0.000	-0.078
NA-ORB	-0.131	-0.029	-0.123	0.070	0.179	0.004	-0.022	**-0.211**	-0.146	-0.134

MT-PNS	-0.119	-0.038	-0.056	0.044	-0.055	0.044	-0.159	-0.002	**-0.203**	0.004
PNS-APET	-0.117	0.012	-0.109	**0.315**	-0.157	-0.018	**-0.339**	0.035	**0.329**	-0.016
APET-BA	-0.071	0.013	-0.056	0.048	-0.001	-0.036	0.023	-0.067	0.131	-0.044
APET-TS	-0.026	0.010	-0.044	-0.142	-0.032	**0.253**	0.141	-0.082	-0.183	**0.212**
BA-EAM	-0.112	0.057	-0.043	0.033	-0.098	0.047	0.090	0.101	-0.051	-0.065
EAM-ZYGO	-0.128	0.022	-0.027	0.140	**-0.328**	-0.027	-0.197	**-0.316**	-0.170	**0.338**
ORB-ZS	-0.032	0.071	0.028	**-0.329**	0.086	**-0.202**	**-0.306**	-0.025	**0.208**	**0.239**
LD-AS	-0.108	-0.022	-0.039	**0.264**	0.048	**-0.321**	0.067	-0.154	-0.166	0.140
BR-LD	0.022	0.030	**-0.670**	**-0.257**	**-0.220**	-0.114	**0.283**	**-0.302**	0.103	-0.140
OPI-LD	-0.092	0.129	0.118	0.121	-0.111	**-0.497**	**-0.280**	0.124	**-0.266**	-0.177
ZAF-AS	-0.165	**0.341**	0.110	-0.081	**-0.377**	0.171	0.035	-0.080	-0.056	-0.165
JP-AS	-0.077	0.183	-0.006	-0.142	**-0.213**	**0.204**	-0.128	**0.224**	-0.035	-0.089
BA-OPI	-0.088	-0.021	-0.025	-0.036	0.006	0.168	0.062	-0.032	0.047	0.127
Percent variance explained (%)	18.863	14.992	9.945	8.452	6.524	6.302	5.039	4.347	3.116	3.025

Table S2.26. First 10 principal components for log-scale ratio cranial data across Asian colobines. Highlighted values depict relatively large loadings (<-0.2 and >0.2).

	PC1	PC2	PC3	PC4	PC5	PC6	PC7	PC8	PC9	PC10
IS-PM	-0.006	-0.085	0.023	-0.078	0.052	-0.115	**-0.210**	0.101	0.019	-0.043
IS-NSL	0.024	-0.014	0.027	-0.025	0.047	-0.002	-0.149	0.001	**-0.207**	0.086
IS-PNS	-0.014	0.036	0.053	0.022	0.009	-0.015	-0.188	0.079	-0.104	**0.240**
PM-ZS	0.045	0.056	-0.008	0.041	0.017	-0.165	-0.161	0.156	-0.172	0.058
PM-ZI	0.133	0.113	-0.080	0.011	0.117	-0.004	-0.168	0.047	-0.106	0.099
PM-MT	-0.032	0.001	0.034	-0.092	-0.003	0.049	-0.147	0.068	-0.154	-0.013
NSL-NA	**0.333**	-0.190	**-0.527**	**0.331**	0.098	-0.197	0.120	-0.135	0.115	-0.114
NSL-ZS	**-0.239**	0.140	**-0.204**	0.181	-0.193	-0.069	-0.193	**0.231**	**0.328**	-0.193
NSL-ZI	0.011	0.039	-0.066	0.082	0.056	-0.019	-0.116	-0.001	0.193	-0.040
NA-BR	-0.054	0.015	0.043	0.005	-0.116	-0.124	**-0.220**	-0.002	-0.137	-0.134
NA-FM	-0.034	0.016	0.005	0.005	-0.100	-0.017	-0.067	0.053	0.012	-0.051
NA-PNS	0.021	0.022	-0.037	0.003	-0.018	-0.011	-0.165	-0.059	-0.063	-0.019

BR-ZAF	0.056	0.033	0.068	-0.075	**-0.268**	-0.142	**-0.238**	-0.158	-0.029	0.041
BR-APET	-0.028	0.068	0.059	-0.106	-0.060	0.094	-0.098	-0.043	0.003	0.024
ZAF-FM	**0.390**	**0.610**	0.149	-0.057	**0.317**	0.147	0.093	0.129	-0.002	-0.035
TS-MT	0.135	0.003	0.002	0.130	-0.102	-0.149	**0.285**	-0.030	**-0.206**	0.160
ZAF-BA	-0.119	-0.168	0.032	-0.136	-0.021	0.087	0.007	-0.032	0.034	-0.108
ZAF-EAM	-0.166	**-0.241**	0.048	-0.018	0.057	0.076	0.065	-0.011	-0.047	-0.059
ZAF-ZYGO	**-0.265**	**-0.344**	-0.060	0.119	**0.438**	0.197	0.186	0.149	-0.088	0.091
AS-EAM	-0.125	0.059	**0.216**	-0.034	-0.010	**-0.286**	0.144	**0.316**	0.087	-0.038
FM-ZS	**0.217**	-0.165	-0.043	-0.079	-0.060	0.082	-0.021	-0.175	-0.009	**-0.251**
FM-MT	0.099	0.016	-0.046	-0.119	0.072	0.035	-0.013	-0.091	0.029	-0.052
ZS-ZI	**0.206**	-0.158	**0.201**	-0.054	**0.360**	**-0.219**	-0.079	**-0.343**	**0.329**	-0.004
ZI-MT	-0.064	0.001	0.031	-0.123	0.194	**0.220**	0.094	-0.176	**-0.341**	0.096
ZI-ZYGO	-0.118	-0.102	0.108	0.153	**0.221**	0.026	-0.050	0.100	-0.041	-0.029
NA-ORB	0.175	-0.090	**-0.332**	0.147	-0.119	**0.366**	-0.123	**0.374**	-0.029	0.157
MT-PNS	0.134	0.053	-0.033	0.019	-0.012	-0.147	**0.252**	0.149	**0.218**	**0.439**
PNS-APET	0.109	-0.024	-0.115	**-0.286**	-0.074	-0.089	**0.329**	0.109	-0.114	**-0.415**
APET-BA	0.004	-0.006	0.011	-0.121	-0.038	0.124	0.014	**-0.207**	0.198	-0.086
APET-TS	-0.043	0.023	**0.302**	**0.565**	**-0.230**	0.032	0.083	**-0.331**	-0.019	0.193
BA-EAM	-0.004	-0.032	0.144	-0.016	-0.030	0.013	0.011	0.029	0.041	0.002
EAM-ZYGO	0.041	-0.005	-0.032	-0.118	**-0.371**	-0.057	**0.414**	-0.017	-0.180	0.064
ORB-ZS	**-0.528**	**0.470**	**-0.402**	0.026	0.142	-0.077	0.113	**-0.324**	-0.061	-0.021
LD-AS	0.028	-0.101	-0.099	**-0.248**	-0.189	**0.219**	-0.103	-0.149	0.009	**0.201**
BR-LD	-0.036	0.110	0.174	0.078	-0.130	**0.541**	0.167	0.003	**0.379**	-0.138
OPI-LD	-0.121	-0.062	-0.097	**-0.381**	-0.014	-0.133	0.007	-0.038	**0.261**	**0.345**
ZAF-AS	-0.110	-0.088	0.142	0.014	-0.088	-0.099	0.088	0.083	0.026	-0.045
JP-AS	-0.108	-0.023	**0.204**	0.058	0.080	-0.171	0.115	**0.208**	0.062	-0.126
BA-OPI	0.051	0.015	0.103	0.177	-0.032	-0.001	-0.074	-0.062	**-0.238**	**-0.285**
Percent variance explained (%)	20.312	15.530	9.585	6.841	6.603	5.335	4.966	4.175	3.467	3.207

Table S2.27. First 10 principal components for raw cranial data across African colobines. Highlighted values depict relatively large loadings (<-0.2 and >0.2).

	PC1	PC2	PC3	PC4	PC5	PC6	PC7	PC8	PC9	PC10
IS-PM	-0.024	-0.004	0.011	0.002	-0.011	-0.001	0.034	-0.059	0.044	0.060
IS-NSL	**-0.225**	0.024	0.170	**-0.261**	0.097	0.044	0.016	-0.131	0.083	-0.159
IS-PNS	**-0.250**	0.016	**0.209**	-0.106	0.068	-0.028	0.172	**-0.247**	0.106	**0.273**
PM-ZS	**-0.215**	-0.039	0.100	-0.196	0.054	0.090	-0.167	-0.166	0.054	-0.079
PM-ZI	-0.179	-0.022	0.145	**-0.255**	0.043	0.091	-0.046	-0.116	0.000	0.120
PM-MT	-0.130	-0.010	0.081	0.030	0.109	-0.051	0.127	**-0.425**	**0.244**	0.062
NSL-NA	-0.053	-0.027	0.059	-0.079	0.135	-0.045	0.056	0.019	-0.116	0.153
NSL-ZS	-0.066	-0.070	-0.091	-0.040	0.022	**0.264**	**-0.434**	-0.161	0.052	0.033
NSL-ZI	-0.137	-0.026	0.066	-0.147	0.016	0.059	-0.037	-0.041	-0.108	0.122
NA-BR	**-0.240**	**0.406**	-0.147	0.065	**-0.533**	-0.111	-0.066	-0.054	0.054	-0.035
NA-FM	-0.102	-0.007	0.009	-0.015	-0.030	0.098	-0.068	0.005	-0.083	0.144
NA-PNS	-0.164	0.025	0.099	0.000	0.012	-0.013	0.109	-0.052	-0.034	0.098
BR-ZAF	**-0.206**	**0.453**	0.002	**0.312**	-0.036	-0.179	**-0.291**	-0.059	-0.172	**0.211**
BR-APET	-0.124	0.135	0.103	**0.382**	-0.084	0.125	0.123	0.093	-0.065	-0.197
ZAF-FM	0.010	**0.207**	**0.408**	-0.045	**-0.205**	0.177	0.077	0.090	0.177	**-0.225**
TS-MT	**-0.288**	-0.013	0.100	**-0.306**	-0.083	0.041	-0.142	**0.353**	**-0.335**	-0.047
ZAF-BA	**-0.311**	-0.181	**-0.335**	0.114	0.170	-0.174	0.071	-0.049	-0.058	0.122
ZAF-EAM	**-0.269**	-0.185	**-0.301**	0.028	-0.045	-0.118	0.107	-0.075	-0.060	-0.200
ZAF-ZYGO	-0.133	**-0.311**	**-0.273**	-0.079	**-0.357**	**0.200**	**0.260**	0.012	0.011	-0.183
AS-EAM	-0.125	-0.090	0.137	0.108	0.067	-0.057	-0.148	**0.375**	**0.403**	0.042
FM-ZS	-0.075	0.048	0.089	-0.091	-0.028	-0.069	**0.267**	0.086	0.007	0.129
FM-MT	-0.196	0.089	0.137	-0.028	-0.100	0.029	0.177	-0.010	0.175	-0.088
ZS-ZI	-0.087	0.019	0.129	-0.052	-0.013	-0.134	**0.253**	0.063	-0.180	0.075
ZI-MT	-0.039	0.031	0.003	0.094	0.018	-0.074	0.141	**-0.219**	**0.286**	-0.092
ZI-ZYGO	-0.151	-0.170	-0.065	0.020	**-0.341**	0.167	0.064	0.094	0.098	0.165
NA-ORB	-0.068	0.018	0.042	-0.054	0.178	0.025	-0.020	0.108	-0.155	0.048
MT-PNS	-0.118	0.020	0.082	-0.077	0.008	0.034	-0.045	0.118	-0.043	0.051

PNS-APET	-0.144	-0.054	-0.044	-0.174	0.004	0.022	**-0.212**	0.091	-0.035	**-0.250**
APET-BA	-0.076	-0.013	0.021	0.016	0.012	0.025	0.050	-0.003	-0.061	0.087
APET-TS	-0.041	0.036	0.028	0.009	-0.010	-0.041	0.084	0.056	-0.065	0.060
BA-EAM	-0.137	-0.014	0.063	0.050	0.059	0.065	-0.009	0.181	0.096	0.095
EAM-ZYGO	-0.149	0.141	0.041	-0.012	**0.263**	**-0.231**	0.012	-0.092	-0.143	**-0.627**
ORB-ZS	-0.028	-0.035	-0.069	0.027	-0.046	**0.270**	**-0.370**	**-0.242**	0.148	-0.067
LD-AS	-0.095	**0.227**	-0.150	0.071	0.114	**0.469**	0.168	-0.128	**-0.275**	0.094
BR-LD	-0.076	**-0.465**	**0.479**	**0.498**	-0.105	0.086	-0.073	-0.159	**-0.319**	-0.074
OPI-LD	-0.120	0.179	-0.156	**0.202**	**0.371**	**0.490**	0.197	0.180	0.093	-0.088
ZAF-AS	**-0.344**	-0.129	-0.104	**0.206**	0.116	**-0.223**	-0.137	0.123	0.131	0.040
JP-AS	-0.116	-0.063	0.045	0.106	0.129	0.045	-0.033	**0.273**	**0.296**	0.031
BA-OPI	-0.031	-0.015	-0.013	0.022	-0.136	0.030	-0.005	-0.017	0.000	-0.008
Percent variance explained (%)	27.647	8.532	7.583	6.608	5.563	4.813	4.313	3.367	3.279	2.686

Table S2.28. First 10 principal components for log-scale ratio cranial data across African colobines. Highlighted values depict relatively large loadings (<-0.2 and >0.2).

	PC1	PC2	PC3	PC4	PC5	PC6	PC7	PC8	PC9	PC10
IS-PM	0.008	0.026	0.035	0.180	0.048	-0.003	0.035	0.128	-0.025	-0.040
IS-NSL	-0.051	-0.015	-0.183	**-0.251**	-0.120	-0.002	0.052	0.033	-0.191	-0.158
IS-PNS	-0.052	-0.013	-0.030	-0.017	0.006	0.060	0.058	0.064	0.011	-0.025
PM-ZS	0.027	-0.057	-0.030	**-0.250**	0.066	0.073	0.046	0.034	-0.053	0.106
PM-ZI	-0.019	-0.020	-0.032	-0.187	0.062	0.057	-0.035	0.042	0.008	-0.139
PM-MT	0.003	0.008	0.027	0.106	-0.023	0.035	0.129	0.151	-0.070	-0.014
NSL-NA	-0.128	**-0.246**	**0.738**	-0.082	**0.409**	0.158	-0.127	0.150	0.049	0.057
NSL-ZS	**0.333**	0.092	0.098	-0.147	-0.037	-0.162	0.000	-0.134	0.043	-0.007
NSL-ZI	-0.003	-0.017	-0.043	-0.061	0.006	0.000	-0.020	0.009	0.056	-0.164
NA-BR	0.000	0.040	-0.100	0.074	-0.056	0.038	-0.101	0.007	-0.160	0.072
NA-FM	0.036	0.002	-0.032	0.027	-0.027	-0.047	-0.021	-0.055	0.040	-0.121
NA-PNS	-0.029	-0.006	0.037	0.029	-0.002	-0.007	0.022	-0.014	-0.031	-0.055
BR-ZAF	0.001	0.027	0.043	0.057	-0.195	0.115	0.057	-0.043	-0.050	-0.030

BR-APET	-0.004	0.061	-0.007	0.151	-0.071	-0.020	0.055	0.022	0.075	-0.049
ZAF-FM	**-0.406**	**0.777**	0.056	-0.093	0.092	-0.112	-0.127	0.044	-0.040	0.158
TS-MT	-0.076	-0.103	-0.166	**-0.383**	0.058	0.061	-0.150	**-0.216**	0.006	0.121
ZAF-BA	0.094	-0.194	-0.074	0.045	-0.056	-0.004	0.087	0.093	0.049	0.100
ZAF-EAM	0.096	**-0.201**	-0.127	0.046	0.017	-0.020	0.015	0.085	-0.106	0.151
ZAF-ZYGO	0.149	-0.158	**-0.238**	0.141	**0.334**	**-0.287**	**-0.325**	0.101	-0.072	0.190
AS-EAM	0.008	0.043	-0.033	-0.010	0.037	-0.092	**0.350**	-0.081	0.135	-0.034
FM-ZS	-0.192	-0.041	-0.046	0.105	0.036	0.047	-0.115	0.089	-0.146	**-0.333**
FM-MT	-0.047	0.060	-0.045	0.022	0.008	0.034	-0.011	0.067	-0.115	-0.017
ZS-ZI	**-0.380**	-0.149	**-0.202**	0.036	0.133	**0.282**	-0.030	0.198	**0.234**	**-0.295**
ZI-MT	0.016	0.080	0.091	**0.280**	-0.051	-0.012	**0.211**	**0.235**	**-0.326**	**0.248**
ZI-ZYGO	0.056	-0.004	-0.177	0.071	**0.237**	**-0.225**	-0.153	0.021	0.023	0.175
NA-ORB	-0.097	-0.128	**0.365**	-0.182	**-0.220**	**-0.545**	0.030	**-0.256**	-0.119	-0.167
MT-PNS	-0.073	-0.018	-0.146	**-0.305**	-0.018	0.081	-0.028	-0.174	**0.201**	0.025
PNS-APET	0.062	-0.052	-0.058	**-0.299**	0.033	-0.077	0.024	0.179	-0.189	0.029
APET-BA	-0.033	-0.005	0.003	0.037	-0.040	-0.101	-0.044	0.064	**0.383**	**0.398**
APET-TS	-0.132	-0.079	0.034	**0.288**	-0.027	**0.339**	-0.127	**-0.661**	-0.037	**0.234**
BA-EAM	-0.009	-0.001	0.000	0.024	-0.019	-0.008	0.058	-0.102	0.126	0.109
EAM-ZYGO	-0.086	-0.066	0.068	-0.111	**-0.299**	**0.253**	**0.232**	0.079	**-0.255**	**0.269**
ORB-ZS	**0.637**	**0.341**	0.115	-0.115	0.126	**0.385**	-0.067	0.009	0.050	-0.134
LD-AS	0.058	0.021	0.072	0.149	**-0.333**	-0.018	**-0.453**	0.047	0.106	-0.096
BR-LD	0.020	0.098	-0.020	0.189	**0.238**	-0.156	**0.415**	-0.120	**0.323**	-0.161
OPI-LD	0.064	-0.015	0.087	0.113	**-0.448**	-0.039	**-0.219**	**0.217**	**0.302**	-0.078
ZAF-AS	0.053	-0.096	-0.072	0.025	-0.045	0.001	0.173	0.013	0.028	0.024
JP-AS	0.029	-0.016	0.027	0.069	-0.016	-0.030	0.190	-0.075	0.107	-0.033
BA-OPI	0.066	0.024	-0.035	**0.228**	0.156	-0.052	-0.090	**-0.250**	**-0.368**	**-0.314**
Percent variance explained (%)	13.960	11.805	7.866	7.138	6.466	5.404	4.717	4.338	3.899	3.611

Table S2.29. First 10 principal components for raw mandibular data across Cercopithecidae. Highlighted values depict relatively large loadings (<-0.2 and >0.2).

	PC1	PC2	PC3	PC4	PC5	PC6	PC7	PC8	PC9	PC10
MO-GG	-0.136	0.052	0.040	0.025	0.063	**0.320**	**-0.400**	**0.228**	**0.482**	**0.228**
GG-GH	-0.098	-0.101	0.012	0.033	0.022	0.010	0.010	0.130	-0.130	**0.225**
GH-IMA	**-0.278**	**0.558**	**0.208**	-0.148	**-0.226**	**0.215**	**0.286**	-0.026	-0.022	**-0.283**
IMA-PMA	**-0.232**	**-0.680**	0.150	0.022	0.045	0.064	0.097	0.059	0.114	**-0.243**
PMA-CONL	-0.129	**0.304**	**-0.332**	-0.114	**0.381**	**-0.419**	-0.089	**0.226**	-0.049	0.164
CONL-CONM	-0.085	-0.012	-0.016	-0.029	0.001	0.008	0.003	0.126	-0.025	0.019
CONL-COR	-0.097	-0.024	**0.242**	0.146	**0.478**	0.027	**0.279**	**0.307**	**-0.358**	0.114
COR-RAMA	**-0.310**	-0.054	**-0.542**	-0.074	**-0.360**	0.137	0.022	-0.198	-0.142	0.118
RAMA-PMA	**-0.261**	-0.070	-0.057	0.154	0.005	**0.407**	**0.292**	0.107	-0.092	0.051
COR-IMA	**-0.336**	**-0.217**	**-0.379**	**-0.260**	0.041	**-0.213**	-0.073	**0.230**	-0.026	**-0.317**
MFO-ALV	**-0.223**	0.052	0.017	**0.558**	**-0.229**	**-0.218**	0.007	**0.201**	0.026	-0.011
MO-MP3	-0.075	0.053	0.016	-0.116	0.006	0.182	-0.084	**0.218**	**0.333**	0.102
MP3-BDM1	-0.119	0.002	0.055	-0.125	0.028	0.183	-0.058	0.113	-0.044	0.161
BDM1-RAMA	-0.146	-0.006	0.192	0.129	-0.019	**-0.301**	**-0.206**	-0.167	0.167	-0.093
CON-MALV	**-0.405**	0.086	0.011	**0.419**	0.136	-0.117	0.092	**-0.324**	0.159	0.045
MFO-CONM	-0.196	-0.001	-0.029	**-0.226**	**0.527**	0.141	0.071	**-0.552**	0.158	0.005
RAMA-GH	**-0.215**	0.007	**0.374**	**-0.300**	-0.078	**-0.213**	-0.062	0.117	0.110	**-0.206**
MP3-MEN	-0.150	0.054	0.082	0.131	0.111	**0.298**	**-0.676**	-0.041	**-0.453**	-0.155
MEN-GH	-0.054	-0.013	-0.039	-0.014	-0.003	0.016	0.153	0.158	**0.294**	**0.318**
ALV-IMA	-0.191	**0.214**	-0.077	0.039	0.108	0.046	-0.006	**0.202**	0.087	**-0.304**
ALV-RAMA	-0.037	-0.020	-0.094	-0.047	0.001	0.107	0.064	0.067	-0.125	0.186
LDM1-BDM1	**-0.203**	-0.037	0.139	0.048	-0.120	-0.179	-0.130	-0.195	-0.077	**0.376**
LDM1-GH	**-0.294**	-0.044	**0.316**	**-0.382**	**-0.210**	-0.151	-0.003	-0.013	**-0.227**	**0.343**
Percent variance explained (%)	40.055	16.449	7.680	6.585	4.564	3.760	3.589	3.186	2.453	2.311

Table S2.30. First 10 principal components for log-scale ratio mandibular data across Cercopithecidae. Highlighted values depict relatively large loadings (<-0.2 and >0.2).

	PC1	PC2	PC3	PC4	PC5	PC6	PC7	PC8	PC9	PC10
MO-GG	-0.148	0.043	0.163	**-0.319**	**0.312**	-0.113	**-0.409**	-0.100	-0.063	**-0.331**

GG-GH	**0.279**	-0.049	0.011	0.170	0.122	**-0.303**	**0.601**	**-0.366**	**0.326**	**-0.252**
GH-IMA	**-0.302**	0.049	0.041	0.051	-0.091	0.038	-0.054	0.119	**0.216**	**0.283**
IMA-PMA	**0.697**	0.053	0.100	-0.130	0.011	**0.219**	-0.164	-0.093	-0.166	0.042
PMA-CONL	**-0.431**	-0.078	-0.043	0.154	**-0.236**	0.160	**0.243**	-0.166	-0.182	-0.162
CONL-CONM	0.011	-0.039	0.026	0.035	-0.017	0.062	0.017	-0.132	0.089	**0.467**
CONL-COR	0.101	0.069	0.104	-0.023	**-0.672**	**-0.500**	-0.195	-0.111	-0.131	0.031
COR-RAMA	0.004	-0.098	-0.170	0.055	**0.203**	**0.376**	0.084	-0.121	-0.174	0.124
RAMA-PMA	0.062	-0.007	-0.022	0.008	-0.025	0.019	-0.075	-0.191	-0.170	0.157
COR-IMA	0.062	-0.026	0.021	0.012	0.011	**0.248**	0.068	-0.128	-0.106	0.087
MFO-ALV	0.013	**0.322**	**-0.534**	**0.353**	0.134	-0.136	**-0.297**	-0.150	-0.023	-0.025
MO-MP3	-0.200	-0.102	**0.295**	-0.139	**0.211**	-0.023	**-0.233**	**-0.371**	**0.294**	-0.172
MP3-BDM1	-0.022	-0.056	0.147	-0.098	-0.009	-0.024	0.028	0.019	0.113	0.185
BDM1-RAMA	0.074	**0.313**	-0.063	0.091	-0.025	0.089	0.014	**0.450**	**0.288**	**-0.336**
CON-MALV	-0.033	0.166	-0.137	0.117	-0.052	0.077	-0.050	0.006	-0.164	-0.188
MFO-CONM	-0.024	-0.035	**0.224**	-0.150	**-0.241**	**0.263**	**0.202**	0.135	**-0.317**	**-0.347**
RAMA-GH	0.000	0.067	0.170	-0.014	-0.066	0.069	-0.011	0.182	**0.310**	0.148
MP3-MEN	-0.091	**0.263**	**-0.213**	**-0.581**	**0.249**	**-0.335**	**0.353**	0.189	**-0.234**	**0.203**
MEN-GH	0.015	**-0.238**	**0.369**	**0.502**	**0.359**	**-0.332**	-0.009	**0.359**	**-0.352**	0.074
ALV-IMA	**-0.231**	0.032	-0.083	0.056	-0.017	0.013	0.019	-0.109	-0.075	0.036
ALV-RAMA	0.074	**-0.762**	**-0.472**	-0.168	-0.076	-0.091	-0.120	**0.217**	0.125	-0.118
LDM1-BDM1	0.065	0.110	-0.036	0.040	-0.022	0.111	-0.007	**0.228**	0.163	-0.077
LDM1-GH	0.025	0.004	0.103	-0.021	-0.063	0.114	-0.003	0.134	**0.234**	0.172
Percent variance explained (%)	17.117	15.802	9.824	8.963	7.800	7.141	6.184	4.864	4.434	3.430

Table S2.31. First 10 principal components for raw mandibular data across Cercopithecinae. Highlighted values depict relatively large loadings (<-0.2 and >0.2).

	PC1	PC2	PC3	PC4	PC5	PC6	PC7	PC8	PC9	PC10
MO-GG	-0.137	0.053	0.040	0.022	0.061	**-0.453**	**-0.201**	**-0.248**	**0.521**	0.139
GG-GH	-0.098	-0.102	0.017	0.034	0.029	-0.026	0.031	-0.141	-0.093	**0.228**
GH-IMA	**-0.277**	**0.554**	**0.206**	-0.166	**-0.227**	-0.079	**0.332**	0.075	-0.078	**-0.266**

IMA-PMA	**-0.231**	**-0.681**	0.145	0.008	0.031	-0.025	0.122	-0.030	0.071	**-0.257**
PMA-CONL	-0.131	**0.301**	**-0.336**	-0.093	**0.381**	**0.334**	**-0.216**	**-0.270**	-0.023	0.153
CONL-CONM	-0.085	-0.012	-0.019	-0.034	0.001	-0.009	0.023	-0.123	-0.028	0.014
CONL-COR	-0.097	-0.036	**0.250**	0.126	**0.470**	0.074	**0.285**	**-0.301**	**-0.344**	0.166
COR-RAMA	**-0.312**	-0.047	**-0.541**	-0.056	**-0.365**	-0.107	0.030	0.197	-0.123	0.156
RAMA-PMA	**-0.263**	-0.080	-0.040	0.135	-0.019	**-0.268**	**0.439**	-0.041	-0.106	0.073
COR-IMA	**-0.336**	**-0.219**	**-0.391**	**-0.244**	0.044	0.157	-0.133	**-0.245**	-0.075	**-0.313**
MFO-ALV	**-0.222**	0.053	0.036	**0.561**	**-0.228**	0.187	-0.053	**-0.224**	0.015	-0.032
MO-MP3	-0.075	0.055	0.008	-0.123	0.002	**-0.212**	0.021	**-0.224**	**0.345**	0.038
MP3-BDM1	-0.122	0.000	0.056	-0.130	0.025	-0.194	0.017	-0.106	-0.021	0.174
BDM1-RAMA	-0.144	-0.009	0.193	0.135	-0.009	0.195	**-0.309**	0.123	0.172	-0.129
CON-MALV	**-0.405**	0.084	0.024	**0.425**	0.140	0.153	0.021	**0.304**	0.166	0.029
MFO-CONM	-0.198	-0.005	-0.045	**-0.220**	**0.530**	-0.068	0.086	**0.559**	0.169	0.001
RAMA-GH	**-0.213**	0.009	**0.357**	**-0.312**	-0.071	0.164	-0.133	-0.134	0.079	**-0.244**
MP3-MEN	-0.148	0.055	0.089	0.128	0.132	**-0.548**	**-0.513**	0.028	**-0.445**	-0.085
MEN-GH	-0.054	-0.011	-0.035	-0.017	-0.008	0.046	0.182	-0.159	**0.333**	**0.268**
ALV-IMA	-0.190	**0.221**	-0.076	0.052	0.106	-0.058	0.069	-0.163	0.027	**-0.324**
ALV-RAMA	-0.038	-0.016	-0.091	-0.045	0.003	-0.070	0.101	-0.056	-0.095	0.191
LDM1-BDM1	**-0.204**	-0.036	0.149	0.053	-0.114	0.131	**-0.219**	0.166	-0.002	**0.386**
LDM1-GH	**-0.294**	-0.035	**0.308**	**-0.387**	**-0.206**	0.156	-0.098	0.001	-0.166	**0.365**
Percent variance explained (%)	41.218	16.388	7.451	6.471	4.408	3.674	3.530	3.082	2.490	2.240

Table S2.32. First 10 principal components for log-scale ratio mandibular data across Cercopithecinae. Highlighted values depict relatively large loadings (<-0.2 and >0.2).

	PC1	PC2	PC3	PC4	PC5	PC6	PC7	PC8	PC9	PC10
MO-GG	-0.140	0.053	0.180	**-0.306**	**0.305**	-0.099	**-0.420**	-0.116	0.082	**-0.300**
GG-GH	**0.266**	-0.080	0.020	0.136	0.101	**-0.311**	**0.597**	**-0.435**	**-0.280**	**-0.249**
GH-IMA	**-0.288**	0.073	0.046	0.041	-0.096	0.043	-0.052	0.096	**-0.226**	**0.239**
IMA-PMA	**0.705**	-0.026	0.071	-0.130	0.034	**0.228**	-0.155	-0.052	0.181	0.058
PMA-CONL	**-0.428**	-0.031	-0.068	0.124	**-0.249**	0.164	**0.245**	-0.134	0.198	-0.124

CONL-CONM	0.005	-0.047	0.026	0.029	-0.038	0.076	0.034	-0.136	-0.074	**0.516**
CONL-COR	0.125	0.057	0.070	-0.025	**-0.684**	**-0.477**	**-0.225**	-0.101	0.153	0.026
COR-RAMA	-0.013	-0.082	-0.166	0.054	**0.217**	**0.359**	0.103	-0.083	0.174	0.116
RAMA-PMA	0.068	-0.013	-0.025	0.004	-0.017	0.030	-0.067	-0.152	0.183	0.146
COR-IMA	0.061	-0.034	0.005	-0.005	0.007	**0.246**	0.081	-0.103	0.122	0.100
MFO-ALV	0.034	**0.343**	**-0.454**	**0.419**	0.183	-0.142	**-0.288**	-0.154	0.049	0.004
MO-MP3	**-0.212**	-0.100	**0.295**	-0.172	0.193	-0.004	**-0.232**	**-0.414**	**-0.221**	-0.149
MP3-BDM1	-0.023	-0.057	0.129	-0.118	-0.022	-0.025	0.029	0.022	-0.132	0.163
BDM1-RAMA	0.103	**0.305**	-0.038	0.108	-0.010	0.074	0.006	**0.375**	**-0.346**	**-0.338**
CON-MALV	-0.019	0.175	-0.122	0.135	-0.032	0.078	-0.047	0.023	0.163	**-0.201**
MFO-CONM	-0.019	-0.040	0.167	-0.168	**-0.245**	**0.273**	0.197	0.164	**0.299**	**-0.378**
RAMA-GH	0.005	0.056	0.163	-0.034	-0.084	0.072	-0.014	0.148	**-0.328**	0.144
MP3-MEN	-0.067	**0.281**	-0.199	**-0.549**	**0.266**	**-0.383**	**0.323**	**0.256**	**0.218**	0.194
MEN-GH	-0.018	**-0.277**	**0.457**	**0.499**	**0.300**	**-0.307**	0.023	**0.373**	**0.290**	0.061
ALV-IMA	**-0.232**	0.064	-0.086	0.065	-0.015	0.011	0.023	-0.105	0.103	0.037
ALV-RAMA	-0.009	**-0.734**	**-0.526**	-0.117	-0.023	-0.116	-0.148	0.199	-0.145	-0.126
LDM1-BDM1	0.075	0.112	-0.033	0.045	-0.018	0.100	-0.008	**0.206**	**-0.208**	-0.096
LDM1-GH	0.022	0.002	0.087	-0.034	-0.074	0.112	-0.006	0.121	**-0.255**	0.157
Percent variance explained (%)	17.957	15.391	9.795	9.062	7.747	7.221	6.044	4.910	4.314	3.453

Table S2.33. First 10 principal components for raw mandibular data across Colobinae. Highlighted values depict relatively large loadings (<-0.2 and >0.2).

	PC1	PC2	PC3	PC4	PC5	PC6	PC7	PC8	PC9	PC10
MO-GG	0.115	0.041	0.028	-0.045	-0.118	-0.009	**0.490**	**-0.371**	0.112	**0.239**
GG-GH	0.091	-0.092	-0.023	0.010	0.034	0.071	-0.151	0.052	**0.393**	**-0.700**
GH-IMA	**0.290**	**0.592**	0.163	0.028	**0.353**	**-0.211**	0.150	**0.229**	**-0.244**	-0.087
IMA-PMA	**0.286**	**-0.628**	0.131	-0.183	-0.195	-0.045	0.077	0.168	**-0.256**	0.010
PMA-CONL	0.080	**0.320**	**-0.271**	**0.361**	**-0.452**	**0.330**	-0.200	0.075	0.183	0.148
CONL-CONM	0.087	-0.015	-0.012	-0.013	-0.016	0.075	0.082	-0.010	0.125	-0.024
CONL-COR	0.086	0.125	0.146	**-0.262**	**-0.460**	-0.125	0.013	**0.417**	0.178	0.177

COR-RAMA	**0.238**	-0.132	**-0.541**	0.134	**0.359**	**-0.266**	-0.075	-0.027	0.051	0.106
RAMA-PMA	**0.202**	0.066	**-0.236**	**-0.258**	-0.080	**-0.207**	0.171	**0.222**	0.080	-0.006
COR-IMA	**0.334**	-0.182	**-0.312**	**0.386**	-0.072	0.170	0.084	0.099	**-0.339**	-0.004
MFO-ALV	**0.265**	0.060	-0.127	**-0.455**	0.150	**0.386**	-0.071	0.050	0.065	0.030
MO-MP3	0.063	0.032	0.057	0.038	-0.031	-0.030	0.177	0.016	0.023	-0.006
MP3-BDM1	0.049	0.015	0.043	0.083	-0.023	-0.100	**0.203**	0.053	0.052	-0.090
BDM1-RAMA	**0.207**	0.032	0.179	-0.017	0.019	**0.231**	**-0.267**	**-0.296**	-0.054	0.070
CON-MALV	**0.385**	0.133	-0.092	**-0.338**	-0.082	-0.098	**-0.390**	-0.130	-0.091	0.002
MFO-CONM	0.146	0.048	0.085	0.187	**-0.362**	**-0.592**	**-0.260**	**-0.289**	-0.078	-0.135
RAMA-GH	**0.280**	-0.018	**0.451**	**0.212**	0.073	0.189	-0.072	0.057	-0.074	-0.134
MP3-MEN	**0.200**	0.052	0.004	-0.125	-0.082	0.022	**0.320**	**-0.512**	0.142	-0.184
MEN-GH	0.050	-0.041	-0.083	0.009	-0.028	0.019	0.043	0.040	0.189	**-0.279**
ALV-IMA	0.197	0.118	-0.068	0.112	**-0.208**	0.186	**0.340**	0.051	-0.144	**-0.200**
ALV-RAMA	0.024	-0.068	-0.129	0.042	0.032	-0.147	0.110	0.130	**0.396**	0.031
LDM1-BDM1	0.196	-0.040	0.053	0.026	0.112	0.042	-0.122	-0.173	**0.284**	**0.352**
LDM1-GH	**0.308**	-0.145	**0.332**	**0.300**	0.182	-0.079	0.012	0.172	**0.398**	**0.230**
Percent variance explained (%)	25.702	18.445	10.949	8.367	6.457	5.889	4.282	3.796	3.173	2.285

Table S2.34. First 10 principal components for log-ratio scale mandibular data across Colobinae. Highlighted values depict relatively large loadings (<-0.2 and >0.2).

	PC1	PC2	PC3	PC4	PC5	PC6	PC7	PC8	PC9	PC10
MO-GG	-0.078	-0.117	**0.272**	**0.417**	-0.158	**0.319**	**-0.205**	-0.199	-0.132	**-0.321**
GG-GH	0.109	**0.299**	**-0.362**	**-0.247**	-0.181	**-0.426**	**-0.393**	**-0.218**	0.164	0.121
GH-IMA	-0.158	**-0.260**	0.133	-0.174	0.035	-0.004	0.189	-0.191	**0.537**	-0.002
IMA-PMA	0.076	**0.351**	**-0.318**	**0.363**	**0.228**	0.183	0.024	**0.272**	**-0.210**	0.193
PMA-CONL	-0.054	**-0.409**	0.056	**-0.451**	**-0.228**	-0.030	0.001	0.103	**-0.530**	0.181
CONL-CONM	0.009	0.053	0.012	0.006	-0.017	0.086	-0.031	-0.100	-0.154	**0.243**
CONL-COR	-0.069	**-0.262**	0.031	0.017	**0.673**	**-0.225**	**-0.318**	0.112	-0.092	-0.003
COR-RAMA	0.178	0.136	0.008	-0.152	**-0.286**	**0.233**	0.195	**0.376**	**0.238**	-0.008
RAMA-PMA	0.009	-0.019	0.069	-0.080	0.084	0.142	-0.167	**0.242**	0.177	0.004

COR-IMA	0.022	0.022	-0.124	-0.048	-0.137	0.165	0.102	0.158	-0.129	0.126
MFO-ALV	-0.175	**0.444**	**0.383**	**-0.350**	**0.279**	0.189	-0.030	0.002	0.025	-0.018
MO-MP3	-0.028	-0.192	-0.066	0.154	-0.014	**0.292**	-0.152	**-0.288**	**0.210**	**0.290**
MP3-BDM1	0.026	-0.138	-0.094	0.075	-0.041	0.071	-0.075	-0.050	0.106	0.067
BDM1-RAMA	**-0.260**	0.176	-0.044	0.011	0.067	**-0.237**	**0.479**	**-0.274**	**-0.246**	**-0.284**
CON-MALV	-0.135	0.070	0.075	-0.114	0.126	-0.021	0.067	**0.243**	0.144	-0.121
MFO-CONM	-0.027	**-0.315**	-0.200	**0.220**	-0.049	**-0.308**	0.148	**0.422**	0.149	**-0.239**
RAMA-GH	-0.107	-0.015	-0.193	0.081	0.060	-0.031	0.193	**-0.279**	0.002	0.114
MP3-MEN	**-0.213**	0.194	**0.463**	**0.341**	**-0.357**	**-0.442**	-0.178	0.123	0.004	0.189
MEN-GH	0.108	0.063	**-0.209**	-0.138	-0.143	0.115	**-0.359**	-0.085	-0.077	**-0.633**
ALV-IMA	-0.051	-0.112	0.048	-0.062	-0.071	0.064	-0.040	-0.008	-0.182	0.107
ALV-RAMA	**0.849**	-0.026	**0.323**	0.043	0.127	-0.174	0.183	-0.155	-0.044	-0.025
LDM1-BDM1	-0.040	0.073	-0.070	0.003	-0.013	0.010	**0.218**	-0.069	-0.032	-0.120
LDM1-GH	0.009	-0.015	-0.191	0.086	0.017	0.029	0.150	-0.135	0.069	0.140
Percent variance explained (%)	20.555	12.397	10.348	9.211	7.422	6.658	6.400	5.399	4.695	3.619

Table S2.35. First 10 principal components for raw mandibular data across Papionini. Highlighted values depict relatively large loadings (<-0.2 and >0.2).

	PC1	PC2	PC3	PC4	PC5	PC6	PC7	PC8	PC9	PC10
MO-GG	-0.144	0.069	0.031	-0.026	0.092	**0.374**	-0.147	**-0.422**	**0.493**	-0.043
GG-GH	-0.103	-0.094	0.019	-0.029	0.027	0.009	0.062	-0.133	-0.041	**-0.257**
GH-IMA	**-0.256**	**0.558**	**0.213**	0.178	**-0.286**	0.090	**0.279**	0.096	-0.092	**0.267**
IMA-PMA	**-0.252**	**-0.666**	0.163	-0.056	0.022	0.024	0.114	-0.004	0.016	**0.265**
PMA-CONL	-0.122	**0.296**	**-0.328**	0.141	**0.399**	**-0.348**	-0.129	**-0.232**	-0.102	-0.128
CONL-CONM	-0.089	-0.012	-0.012	0.035	0.004	0.000	0.033	-0.123	-0.051	-0.015
CONL-COR	-0.092	-0.018	**0.250**	-0.116	**0.437**	-0.048	**0.351**	-0.183	**-0.404**	**-0.200**
COR-RAMA	**-0.320**	-0.042	**-0.548**	0.099	**-0.357**	0.132	-0.037	0.169	-0.062	-0.126
RAMA-PMA	**-0.270**	-0.070	-0.041	-0.131	-0.099	**0.297**	**0.421**	-0.007	-0.113	-0.053
COR-IMA	**-0.341**	**-0.241**	**-0.345**	**0.269**	0.082	-0.192	-0.124	-0.183	-0.152	**0.280**
MFO-ALV	**-0.218**	0.082	-0.017	**-0.564**	**-0.204**	**-0.220**	-0.057	**-0.216**	-0.084	0.045

MO-MP3	-0.078	0.036	0.023	0.120	0.002	0.157	0.046	**-0.316**	**0.301**	0.033
MP3-BDM1	-0.128	-0.003	0.048	0.116	0.028	0.192	0.038	-0.124	0.003	-0.158
BDM1-RAMA	-0.143	0.017	0.188	-0.156	0.035	-0.200	**-0.289**	0.084	**0.201**	0.107
CON-MALV	**-0.400**	0.119	-0.015	**-0.427**	0.145	-0.138	0.028	**0.284**	0.184	0.019
MFO-CONM	-0.197	0.015	-0.024	**0.220**	**0.492**	0.110	0.120	**0.526**	**0.286**	0.015
RAMA-GH	**-0.211**	0.005	**0.381**	**0.277**	-0.051	-0.197	-0.122	-0.140	0.010	**0.273**
MP3-MEN	-0.149	0.074	0.075	-0.127	**0.200**	**0.556**	**-0.509**	0.002	**-0.363**	0.014
MEN-GH	-0.055	-0.025	-0.057	0.000	-0.019	-0.134	**0.272**	**-0.204**	**0.333**	**-0.253**
ALV-IMA	-0.174	**0.218**	-0.063	-0.024	0.110	0.022	0.090	-0.136	-0.021	**0.245**
ALV-RAMA	-0.040	-0.020	-0.092	0.058	-0.004	0.087	0.115	-0.033	-0.090	**-0.208**
LDM1-BDM1	**-0.204**	-0.015	0.143	-0.073	-0.095	-0.105	**-0.232**	0.151	0.102	**-0.442**
LDM1-GH	**-0.291**	-0.042	**0.332**	**0.356**	**-0.209**	-0.140	-0.139	0.036	-0.121	**-0.399**
Percent variance explained (%)	41.988	15.097	7.510	6.845	4.330	3.673	3.662	3.095	2.676	2.286

Table S2.36. First 10 principal components for log-scale ratio mandibular data across Papionini. Highlighted values depict relatively large loadings (<-0.2 and >0.2).

	PC1	PC2	PC3	PC4	PC5	PC6	PC7	PC8	PC9	PC10
MO-GG	0.069	-0.157	0.025	-0.194	**-0.289**	**0.326**	**0.432**	0.055	**0.218**	0.162
GG-GH	-0.111	**0.268**	0.079	0.024	-0.040	**0.318**	**-0.535**	**-0.267**	**-0.390**	**0.351**
GH-IMA	0.097	**-0.280**	0.096	-0.047	0.091	-0.113	0.053	0.175	**-0.231**	**-0.238**
IMA-PMA	-0.095	**0.648**	-0.164	-0.133	-0.133	-0.199	0.173	-0.146	0.172	-0.039
PMA-CONL	0.010	**-0.471**	0.105	0.074	0.119	**-0.208**	**-0.236**	**-0.302**	0.078	0.165
CONL-CONM	-0.039	0.005	0.016	-0.030	0.013	-0.087	0.045	**-0.214**	-0.192	**-0.454**
CONL-COR	0.026	0.124	0.010	**-0.210**	**0.816**	0.191	0.168	-0.153	0.111	0.047
COR-RAMA	-0.072	-0.045	-0.096	0.196	**-0.292**	**-0.235**	-0.076	-0.170	0.070	-0.080
RAMA-PMA	-0.028	0.075	-0.037	0.040	0.002	-0.027	0.090	-0.177	0.099	-0.165
COR-IMA	-0.046	0.059	-0.028	-0.032	-0.112	**-0.212**	-0.056	-0.196	0.043	-0.060
MFO-ALV	**0.340**	0.111	0.089	**0.673**	0.047	0.103	**0.267**	-0.129	-0.049	0.043
MO-MP3	-0.084	-0.172	0.123	**-0.287**	**-0.259**	0.183	**0.348**	**-0.224**	**-0.249**	**0.321**
MP3-BDM1	-0.060	-0.037	-0.014	-0.157	-0.020	0.038	-0.018	0.040	-0.111	-0.179

BDM1-RAMA	**0.288**	0.150	0.092	0.037	0.005	-0.112	-0.059	**0.500**	-0.109	**0.290**
CON-MALV	0.168	0.008	0.047	0.183	0.026	-0.105	0.016	0.001	**0.221**	0.173
MFO-CONM	-0.037	-0.075	-0.033	**-0.259**	0.025	**-0.291**	**-0.224**	0.077	**0.463**	**0.265**
RAMA-GH	0.048	0.027	0.062	**-0.201**	0.030	-0.128	0.018	0.185	**-0.292**	-0.115
MP3-MEN	**0.375**	-0.080	**-0.518**	-0.099	-0.120	**0.473**	**-0.318**	0.077	0.171	**-0.256**
MEN-GH	**-0.376**	0.081	**0.602**	0.120	-0.075	**0.330**	-0.164	**0.250**	**0.318**	**-0.270**
ALV-IMA	0.075	**-0.204**	0.051	0.076	0.040	-0.016	-0.024	-0.091	0.072	-0.080
ALV-RAMA	**-0.644**	-0.150	**-0.511**	**0.319**	0.131	0.065	0.124	**0.266**	-0.081	0.143
LDM1-BDM1	0.101	0.087	0.008	0.032	-0.015	-0.130	-0.027	**0.294**	-0.092	0.089
LDM1-GH	-0.006	0.026	-0.005	-0.126	0.009	-0.166	0.004	0.151	**-0.240**	-0.112
Percent variance explained (%)	17.172	14.368	11.283	10.049	8.344	7.095	5.785	4.513	4.067	3.365

Table S2.37. First 10 principal components for raw mandibular data across Cercopithecini. Highlighted values depict relatively large loadings (<-0.2 and >0.2).

	PC1	PC2	PC3	PC4	PC5	PC6	PC7	PC8	PC9	PC10
MO-GG	0.109	0.019	0.066	-0.001	-0.010	**0.483**	-0.140	0.006	0.100	**0.645**
GG-GH	0.073	-0.123	-0.004	0.086	0.000	0.046	-0.100	0.118	0.154	**-0.523**
GH-IMA	**0.364**	**0.500**	**0.219**	-0.112	0.108	0.024	**0.377**	0.137	-0.180	-0.118
IMA-PMA	0.126	**-0.714**	0.091	-0.040	-0.037	0.060	0.098	0.130	**-0.274**	0.056
PMA-CONL	0.173	**0.290**	**-0.343**	-0.004	**-0.358**	**-0.264**	**-0.486**	0.096	0.102	0.098
CONL-CONM	0.071	-0.014	-0.027	-0.029	0.005	0.008	-0.028	0.100	0.010	-0.085
CONL-COR	0.109	-0.090	0.194	**0.346**	**-0.431**	-0.080	-0.009	**0.398**	**0.271**	-0.018
COR-RAMA	**0.277**	-0.078	**-0.473**	-0.167	**0.380**	0.008	0.199	-0.164	**0.351**	-0.023
RAMA-PMA	**0.233**	-0.113	-0.076	0.176	-0.072	0.171	**0.325**	**0.246**	0.175	-0.016
COR-IMA	**0.309**	**-0.201**	**-0.448**	**-0.217**	0.029	-0.015	**-0.229**	**0.247**	**-0.261**	0.001
MFO-ALV	**0.228**	-0.021	0.057	**0.496**	**0.334**	-0.126	-0.119	0.051	-0.051	0.099
MO-MP3	0.073	0.094	0.007	-0.098	-0.021	**0.236**	0.022	0.113	0.035	**0.266**
MP3-BDM1	0.101	0.003	0.117	-0.147	-0.047	0.137	-0.045	0.149	0.158	-0.033
BDM1-RAMA	0.141	-0.073	0.163	0.077	0.077	-0.171	**-0.245**	**-0.289**	**-0.270**	0.020
CON-MALV	**0.417**	-0.021	0.023	**0.374**	-0.076	-0.156	0.150	**-0.374**	0.037	0.114

MFO-CONM	0.193	-0.072	-0.081	**-0.211**	**-0.614**	0.049	**0.259**	**-0.443**	-0.059	-0.055
RAMA-GH	**0.216**	-0.007	**0.361**	**-0.311**	0.018	-0.110	-0.169	0.064	**-0.231**	0.009
MP3-MEN	0.140	0.014	0.114	0.099	0.018	**0.612**	**-0.395**	**-0.280**	0.087	**-0.374**
MEN-GH	0.050	0.019	0.050	-0.089	0.009	0.124	-0.025	0.020	0.073	-0.134
ALV-IMA	**0.255**	0.198	-0.137	0.138	-0.022	0.182	0.010	0.175	**-0.448**	-0.114
ALV-RAMA	0.029	-0.010	-0.068	-0.021	0.014	0.025	0.008	0.093	0.137	-0.053
LDM1-BDM1	0.195	-0.097	0.147	-0.007	0.108	-0.187	-0.153	-0.187	**0.231**	0.061
LDM1-GH	**0.299**	-0.056	**0.333**	**-0.386**	0.084	-0.193	-0.093	0.100	**0.329**	0.007
Percent variance explained (%)	39.541	20.457	7.407	5.277	4.762	3.881	3.184	2.953	2.125	2.022

Table S2.38. First 10 principal components for log-scale ratio mandibular data across Cercopithecini. Highlighted values depict relatively large loadings (<-0.2 and >0.2).

	PC1	PC2	PC3	PC4	PC5	PC6	PC7	PC8	PC9	PC10
MO-GG	-0.112	0.055	**-0.359**	**0.246**	**0.470**	0.097	0.047	0.184	-0.009	0.110
GG-GH	**0.265**	-0.109	0.147	0.199	**-0.558**	**0.252**	**0.500**	**0.235**	-0.179	**0.222**
GH-IMA	**-0.278**	0.085	0.016	-0.057	-0.029	-0.038	-0.102	0.062	-0.172	-0.019
IMA-PMA	**0.722**	-0.026	-0.151	-0.118	**0.210**	-0.125	0.003	0.034	0.156	-0.088
PMA-CONL	**-0.385**	-0.016	**0.235**	**-0.324**	-0.100	0.103	0.121	-0.146	0.162	-0.172
CONL-CONM	0.001	-0.060	0.022	-0.089	-0.079	-0.061	0.084	0.103	-0.051	**-0.637**
CONL-COR	0.128	0.078	0.041	-0.151	-0.034	**0.698**	**-0.415**	0.088	0.191	-0.079
COR-RAMA	-0.005	-0.079	0.128	-0.068	0.100	**-0.407**	**0.255**	0.008	**0.219**	-0.017
RAMA-PMA	0.057	0.004	0.014	-0.059	0.057	0.043	0.042	0.136	**0.231**	-0.019
COR-IMA	0.052	-0.020	0.043	-0.156	0.059	-0.144	0.169	-0.002	0.139	-0.142
MFO-ALV	0.033	**0.281**	**0.464**	**0.372**	0.069	-0.131	**-0.228**	**0.263**	0.174	0.025
MO-MP3	**-0.245**	-0.087	**-0.297**	0.017	0.156	0.044	0.152	**0.460**	-0.153	0.168
MP3-BDM1	-0.023	-0.033	-0.156	-0.049	-0.016	0.091	0.028	0.002	**-0.207**	-0.178
BDM1-RAMA	0.109	**0.278**	0.152	0.038	-0.010	-0.151	-0.165	**-0.268**	**-0.434**	0.196
CON-MALV	-0.015	0.174	0.174	-0.039	0.051	-0.040	-0.057	-0.023	0.134	**0.275**
MFO-CONM	0.010	-0.012	-0.132	**-0.432**	0.054	0.092	0.155	**-0.338**	0.123	**0.450**
RAMA-GH	-0.006	0.068	-0.103	-0.095	-0.045	-0.051	-0.111	-0.028	**-0.348**	-0.100

MP3-MEN	-0.065	0.189	**-0.251**	**0.548**	-0.033	0.167	**0.223**	**-0.565**	0.183	-0.195
MEN-GH	-0.081	-0.138	**-0.420**	0.076	**-0.559**	**-0.341**	**-0.437**	0.048	**0.289**	0.120
ALV-IMA	**-0.238**	0.070	0.164	0.045	0.035	-0.001	0.102	0.068	0.169	0.097
ALV-RAMA	-0.015	**-0.830**	**0.257**	**0.234**	0.191	0.049	**-0.202**	**-0.206**	-0.100	0.076
LDM1-BDM1	0.078	0.108	0.072	-0.034	0.023	-0.090	-0.085	-0.101	**-0.253**	0.023
LDM1-GH	0.012	0.020	-0.061	-0.105	-0.013	-0.057	-0.078	-0.015	**-0.263**	-0.116
Percent variance explained (%)	23.194	13.512	9.922	7.626	7.109	6.674	6.002	5.039	4.312	3.128

Table S2.39. First 10 principal components for raw mandibular data across Asian colobines. Highlighted values depict relatively large loadings (<-0.2 and >0.2).

	PC1	PC2	PC3	PC4	PC5	PC6	PC7	PC8	PC9	PC10
MO-GG	0.037	0.000	-0.097	0.144	**0.223**	0.146	**-0.408**	0.037	0.141	**-0.432**
GG-GH	0.123	0.000	-0.007	-0.101	**-0.226**	-0.057	0.158	**0.235**	**-0.437**	**0.393**
GH-IMA	-0.133	**0.660**	-0.170	-0.057	**0.397**	0.199	0.075	-0.026	-0.012	0.110
IMA-PMA	**0.568**	**-0.301**	0.019	0.097	0.053	**0.309**	0.077	**-0.460**	-0.065	0.014
PMA-CONL	-0.057	0.189	0.056	-0.164	**-0.270**	**-0.603**	**-0.302**	**-0.270**	-0.024	**-0.202**
CONL-CONM	0.099	0.034	0.042	-0.046	-0.058	0.016	0.002	-0.013	-0.029	0.109
CONL-COR	-0.088	0.061	-0.015	-0.067	**-0.259**	0.075	**-0.259**	**-0.312**	**-0.399**	-0.172
COR-RAMA	**0.310**	0.051	**0.302**	**-0.295**	**0.259**	-0.164	**0.225**	**0.303**	0.113	**-0.261**
RAMA-PMA	0.140	0.087	**0.288**	-0.200	0.157	0.169	-0.098	**-0.209**	**-0.213**	-0.107
COR-IMA	**0.446**	0.013	-0.033	0.129	**0.227**	**-0.531**	0.016	-0.122	0.139	0.134
MFO-ALV	0.140	**0.369**	**0.398**	**0.292**	**-0.227**	0.161	-0.005	-0.047	0.033	-0.027
MO-MP3	-0.007	0.062	-0.104	-0.018	0.170	0.071	-0.017	-0.135	-0.035	-0.031
MP3-BDM1	0.023	-0.031	-0.110	0.033	0.077	0.002	-0.031	-0.014	-0.129	0.013
BDM1-RAMA	0.186	0.103	-0.192	0.050	**-0.322**	0.078	0.002	0.099	**0.321**	-0.133
CON-MALV	0.180	**0.326**	**0.248**	**-0.340**	**-0.258**	0.185	-0.105	-0.085	**0.292**	**0.233**
MFO-CONM	0.091	-0.109	**-0.331**	**-0.660**	0.114	0.070	**-0.269**	-0.045	0.089	0.163
RAMA-GH	**0.200**	0.147	**-0.473**	0.151	-0.196	0.060	0.052	-0.099	0.085	0.026
MP3-MEN	**0.200**	-0.060	0.008	0.177	0.023	0.114	**-0.600**	**0.446**	-0.022	0.123
MEN-GH	0.093	-0.081	0.149	-0.046	-0.078	0.030	**-0.204**	**0.238**	-0.173	0.162

ALV-IMA	0.084	**0.219**	-0.007	**0.270**	**0.279**	-0.194	**-0.244**	-0.106	-0.153	**0.349**
ALV-RAMA	0.035	0.005	0.140	-0.084	0.164	0.023	-0.001	0.045	**-0.338**	**-0.273**
LDM1-BDM1	**0.215**	0.142	-0.088	-0.031	-0.168	-0.010	0.034	**0.251**	-0.036	**-0.269**
LDM1-GH	**0.262**	**0.230**	**-0.342**	-0.034	-0.068	-0.036	0.168	0.169	**-0.394**	**-0.237**
Percent variance explained (%)	27.492	18.254	12.827	9.196	6.499	5.482	4.428	3.945	3.330	2.058

Table S2.40. First 10 principal components for log-scale ratio mandibular data across Asian colobines. Highlighted values depict relatively large loadings (<-0.2 and >0.2).

	PC1	PC2	PC3	PC4	PC5	PC6	PC7	PC8	PC9	PC10
MO-GG	-0.046	-0.024	0.153	**0.588**	-0.119	0.135	**-0.292**	**-0.292**	0.176	**0.418**
GG-GH	-0.141	0.057	0.030	**-0.520**	**-0.203**	**-0.503**	0.060	-0.083	0.036	**0.270**
GH-IMA	**0.210**	**0.270**	-0.175	0.180	0.013	**-0.205**	**0.248**	-0.147	**0.232**	-0.053
IMA-PMA	-0.191	-0.193	**0.222**	-0.030	**0.444**	0.108	**-0.245**	**0.448**	-0.080	0.132
PMA-CONL	0.172	**0.226**	-0.171	-0.132	**-0.259**	**0.419**	-0.085	-0.147	**-0.540**	-0.093
CONL-CONM	-0.080	-0.054	-0.037	-0.131	0.153	0.063	0.088	**0.266**	-0.037	0.041
CONL-COR	**0.209**	0.193	0.036	-0.129	**-0.412**	0.110	**-0.437**	**0.357**	0.164	-0.166
COR-RAMA	0.050	-0.166	0.003	-0.114	**0.257**	0.180	**0.311**	**-0.253**	-0.100	0.069
RAMA-PMA	0.133	-0.062	0.000	-0.033	0.020	0.200	0.103	0.148	0.134	0.197
COR-IMA	-0.043	0.024	0.018	-0.002	0.158	0.041	-0.001	-0.048	**-0.363**	0.136
MFO-ALV	-0.003	**-0.243**	**-0.717**	0.049	0.034	0.025	-0.037	0.127	0.192	0.038
MO-MP3	0.175	**0.233**	0.082	**0.271**	0.047	**-0.201**	**0.264**	**0.314**	0.107	-0.005
MP3-BDM1	0.037	0.101	0.132	0.045	-0.025	-0.151	-0.030	0.150	-0.136	0.026
BDM1-RAMA	**-0.322**	0.126	-0.101	-0.063	**0.251**	-0.018	**-0.382**	**-0.330**	0.187	**-0.331**
CON-MALV	0.018	0.026	-0.193	-0.101	0.052	**0.300**	**0.224**	0.050	**0.266**	-0.115
MFO-CONM	0.016	**0.244**	**0.436**	-0.070	0.071	**0.263**	**0.270**	-0.109	0.190	**-0.215**
RAMA-GH	-0.104	**0.206**	0.020	0.038	0.093	-0.182	-0.165	0.022	-0.007	-0.097
MP3-MEN	**-0.506**	**-0.326**	0.060	**0.292**	**-0.393**	-0.074	**0.259**	0.107	-0.161	**-0.440**
MEN-GH	-0.115	**-0.288**	0.172	**-0.223**	**-0.370**	0.152	0.053	-0.116	**0.227**	**0.330**
ALV-IMA	0.045	0.081	-0.140	**0.208**	-0.040	-0.179	0.074	0.007	**-0.368**	**0.227**
ALV-RAMA	**0.605**	**-0.559**	0.179	0.000	0.059	**-0.231**	-0.149	-0.158	-0.043	**-0.308**

LDM1-BDM1	-0.102	0.018	-0.035	-0.090	0.108	-0.063	-0.096	**-0.250**	-0.004	-0.034
LDM1-GH	-0.016	0.110	0.025	-0.034	0.063	-0.188	-0.035	-0.063	-0.070	-0.027
Percent variance explained (%)	24.173	15.780	11.820	10.387	7.777	6.060	4.164	4.008	3.184	3.123

Table S2.41. First 10 principal components for raw mandibular data across African colobines. Highlighted values depict relatively large loadings (<-0.2 and >0.2).

	PC1	PC2	PC3	PC4	PC5	PC6	PC7	PC8	PC9	PC10
MO-GG	0.128	0.033	0.022	-0.057	-0.103	0.029	**0.506**	**-0.417**	-0.007	0.159
GG-GH	0.081	-0.101	-0.034	0.010	0.024	-0.107	-0.053	0.057	**0.392**	**-0.711**
GH-IMA	**0.345**	**0.542**	0.182	0.063	**0.384**	0.175	0.084	**0.261**	**-0.246**	-0.058
IMA-PMA	**0.226**	**-0.656**	0.128	-0.157	**-0.203**	0.071	0.020	0.142	**-0.253**	0.004
PMA-CONL	0.108	**0.337**	**-0.304**	**0.318**	**-0.500**	**-0.258**	-0.128	0.055	**0.229**	0.132
CONL-CONM	0.082	-0.019	-0.008	-0.005	-0.026	-0.085	0.152	-0.016	0.099	-0.018
CONL-COR	0.116	0.105	0.190	**-0.269**	**-0.431**	**0.236**	0.030	**0.400**	**0.207**	**0.215**
COR-RAMA	**0.215**	-0.137	**-0.570**	0.056	**0.398**	**0.207**	-0.077	-0.014	0.065	0.096
RAMA-PMA	**0.211**	0.060	-0.189	**-0.300**	-0.031	**0.208**	0.197	**0.216**	0.065	-0.012
COR-IMA	**0.307**	-0.187	**-0.387**	**0.325**	-0.118	-0.108	0.010	0.134	**-0.353**	0.039
MFO-ALV	**0.265**	0.013	-0.060	**-0.431**	0.107	**-0.430**	-0.025	0.080	0.097	0.028
MO-MP3	0.070	0.018	0.045	0.042	-0.037	0.028	0.196	-0.019	-0.001	-0.049
MP3-BDM1	0.056	0.016	0.026	0.081	-0.007	0.138	**0.211**	0.046	-0.002	-0.095
BDM1-RAMA	**0.206**	0.017	0.171	0.013	0.005	**-0.287**	**-0.265**	**-0.244**	-0.005	0.076
CON-MALV	**0.401**	0.083	-0.032	**-0.347**	-0.032	0.036	**-0.398**	-0.128	-0.005	-0.018
MFO-CONM	0.160	0.049	0.057	0.135	**-0.253**	**0.586**	**-0.357**	**-0.347**	-0.027	-0.180
RAMA-GH	**0.279**	-0.062	**0.402**	**0.299**	0.046	-0.200	-0.053	0.100	-0.059	-0.148
MP3-MEN	**0.210**	0.062	0.023	-0.138	-0.038	-0.020	**0.290**	**-0.483**	0.014	-0.150
MEN-GH	0.046	-0.034	-0.076	0.019	-0.024	-0.021	0.090	0.078	0.152	**-0.275**
ALV-IMA	**0.208**	0.100	-0.083	0.098	**-0.264**	-0.102	**0.276**	0.020	**-0.214**	-0.178
ALV-RAMA	0.017	-0.075	-0.134	0.036	0.036	0.163	0.152	0.060	**0.416**	-0.010
LDM1-BDM1	0.181	-0.061	0.041	0.038	0.108	-0.070	-0.065	-0.196	**0.299**	**0.370**
LDM1-GH	**0.287**	**-0.210**	**0.276**	**0.376**	0.160	0.100	0.102	0.095	**0.373**	**0.229**

Percent variance explained (%)	26.205	18.370	11.111	8.413	6.604	5.597	4.020	3.696	3.050	2.317

Table S2.42. First 10 principal components for log-shape ratio mandibular data across African colobines. Highlighted values depict relatively large loadings (<-0.2 and >0.2).

	PC1	PC2	PC3	PC4	PC5	PC6	PC7	PC8	PC9	PC10
MO-GG	-0.081	-0.143	**0.223**	**0.317**	**-0.357**	**0.361**	-0.031	**0.205**	-0.098	**-0.369**
GG-GH	0.134	**0.343**	**-0.271**	**-0.269**	-0.017	**-0.219**	**-0.640**	0.148	0.100	0.048
GH-IMA	-0.171	**-0.249**	0.086	-0.152	0.077	-0.101	0.136	0.133	**0.593**	0.112
IMA-PMA	0.084	**0.401**	**-0.266**	**0.413**	0.043	0.110	0.114	**-0.256**	**-0.262**	0.162
PMA-CONL	-0.057	**-0.445**	-0.030	**-0.491**	-0.013	-0.018	-0.003	-0.038	**-0.535**	0.142
CONL-CONM	0.013	0.028	0.025	0.003	-0.049	0.108	0.023	0.162	-0.145	**0.307**
CONL-COR	-0.090	**-0.220**	0.018	**0.252**	**0.698**	0.140	**-0.249**	-0.066	-0.056	0.035
COR-RAMA	0.180	0.118	0.020	**-0.247**	**-0.265**	0.053	**0.255**	**-0.419**	**0.208**	0.023
RAMA-PMA	-0.008	-0.015	0.053	-0.059	0.082	**0.235**	-0.055	**-0.243**	0.162	0.042
COR-IMA	0.030	0.029	-0.137	-0.093	-0.132	0.044	0.154	-0.170	-0.140	0.120
MFO-ALV	-0.188	**0.427**	**0.385**	**-0.269**	**0.285**	**0.252**	0.122	0.009	0.006	-0.056
MO-MP3	-0.028	-0.140	-0.125	0.151	-0.162	**0.289**	-0.041	**0.258**	0.166	**0.211**
MP3-BDM1	0.028	-0.124	-0.118	0.062	-0.070	0.083	-0.034	0.017	0.149	0.101
BDM1-RAMA	**-0.248**	0.171	0.020	0.017	0.083	**-0.449**	**0.299**	**0.323**	-0.195	**-0.281**
CON-MALV	-0.144	0.076	0.086	-0.058	0.143	-0.027	0.061	**-0.228**	0.096	-0.160
MFO-CONM	-0.030	**-0.297**	-0.176	**0.220**	-0.009	**-0.364**	-0.063	**-0.430**	0.071	**-0.321**
RAMA-GH	-0.093	0.023	-0.189	0.106	0.013	-0.163	0.135	**0.267**	0.047	0.160
MP3-MEN	**-0.211**	0.075	**0.537**	0.180	**-0.333**	**-0.276**	**-0.411**	-0.136	-0.060	0.174
MEN-GH	0.106	0.054	**-0.256**	-0.192	-0.060	**0.254**	-0.183	0.130	0.019	**-0.567**
ALV-IMA	-0.049	-0.120	-0.001	-0.081	-0.034	0.055	-0.018	-0.004	**-0.208**	0.045
ALV-RAMA	**0.840**	-0.082	**0.359**	0.070	0.149	-0.166	0.091	0.161	-0.020	-0.040
LDM1-BDM1	-0.035	0.073	-0.053	0.007	-0.029	-0.112	**0.206**	0.072	0.006	-0.083
LDM1-GH	0.019	0.017	-0.193	0.114	-0.041	-0.089	0.133	0.106	0.096	0.194
Percent variance explained (%)	21.228	12.208	10.252	8.993	7.442	6.801	6.333	5.582	4.717	3.503

Table series S3. Pearson product-moment correlation values and associated p-values for each analysis (42 tables in total)

Table S3.1. Pearson product-moment correlation values (below diagonal) and associated p-values (above diagonal) for raw skull data across Cercopithecidae.

	PC1	PC2	PC3	PC4	PC5	PC6	PC7	PC8	PC9	PC10	PC11	PC12	PC13	PC14	PC15	PC16	PC17	PC18	PC19	PC20
PC1	1.000	0.338	0.377	0.000	0.938	0.000	0.339	0.875	0.401	0.122	0.090	0.646	0.960	0.292	0.003	0.992	0.041	0.200	0.300	0.000
PC2	0.220	1.000	0.000	0.117	0.007	0.649	0.011	0.410	0.108	0.429	0.010	0.152	0.234	0.163	0.294	0.000	0.515	0.823	0.162	0.648
PC3	-0.203	-0.807	1.000	0.039	0.006	0.826	0.000	0.521	0.095	0.205	0.015	0.106	0.542	0.276	0.243	0.001	0.770	0.917	0.019	0.457
PC4	0.729	-0.353	0.453	1.000	0.097	0.000	0.000	0.433	0.353	0.276	0.969	0.041	0.402	0.184	0.137	0.030	0.184	0.439	0.010	0.001
PC5	-0.018	-0.572	0.582	0.372	1.000	0.922	0.134	0.607	0.037	0.337	0.545	0.180	0.125	0.060	0.967	0.000	0.645	0.989	0.845	0.776
PC6	-0.845	-0.105	-0.051	-0.777	0.023	1.000	0.014	0.260	0.989	0.168	0.379	0.204	0.374	0.687	0.020	0.607	0.326	0.293	0.004	0.000
PC7	-0.219	0.540	-0.747	-0.706	-0.338	0.526	1.000	0.729	0.083	0.703	0.291	0.029	0.710	0.833	0.445	0.011	0.927	0.155	0.000	0.011
PC8	0.037	-0.190	0.148	0.181	0.119	-0.257	-0.080	1.000	0.886	0.924	0.574	0.102	0.374	0.615	0.080	0.097	0.674	0.086	0.148	0.635
PC9	0.193	0.361	-0.374	-0.213	-0.458	-0.003	0.387	0.033	1.000	0.081	0.366	0.167	0.862	0.432	0.029	0.083	0.167	0.752	0.681	0.895
PC10	0.348	0.182	-0.288	0.249	-0.220	-0.312	-0.088	0.022	-0.389	1.000	0.793	0.489	0.841	0.011	0.605	0.266	0.510	0.535	0.750	0.794
PC11	0.379	0.548	-0.523	-0.009	-0.140	-0.202	0.242	-0.130	0.208	0.061	1.000	0.923	0.994	0.167	0.135	0.413	0.337	0.638	0.390	0.296
PC12	0.107	-0.324	0.362	0.449	0.304	-0.289	-0.477	0.366	-0.313	0.160	0.022	1.000	0.057	0.622	0.803	0.027	0.716	0.784	0.013	0.393
PC13	-0.012	0.271	-0.141	-0.193	-0.345	-0.205	-0.086	-0.204	-0.040	-0.047	-0.002	-0.421	1.000	0.460	0.501	0.078	0.028	0.535	0.307	0.105
PC14	0.241	-0.316	0.249	0.302	0.417	-0.093	-0.049	0.117	0.181	-0.543	0.313	0.114	-0.170	1.000	0.015	0.014	0.865	0.460	0.655	0.199
PC15	0.621	0.240	-0.266	0.335	-0.009	-0.504	0.176	0.390	0.476	-0.120	0.337	0.058	-0.155	0.522	1.000	0.555	0.056	0.970	0.911	0.144
PC16	-0.002	-0.743	0.680	0.475	0.790	-0.119	-0.540	0.372	-0.387	-0.254	-0.189	0.482	-0.393	0.526	0.137	1.000	0.943	0.928	0.126	0.924
PC17	0.448	0.151	-0.068	0.302	0.107	-0.225	-0.021	-0.097	0.313	0.152	0.221	0.085	-0.480	0.039	0.423	0.017	1.000	0.053	0.272	0.558
PC18	-0.291	0.052	-0.024	-0.178	0.003	0.241	0.322	0.383	-0.073	-0.143	0.109	0.064	-0.143	0.170	0.009	-0.021	-0.428	1.000	0.946	0.156
PC19	-0.238	0.316	-0.505	-0.549	-0.045	0.596	0.780	-0.327	0.095	0.074	0.198	-0.530	-0.234	-0.104	-0.026	-0.345	0.251	0.016	1.000	0.009
PC20	0.695	0.106	0.172	0.678	-0.066	-0.780	-0.545	-0.110	0.031	0.061	0.239	0.197	0.364	0.292	0.330	0.022	0.136	-0.321	-0.556	1.000

Table S3.2. Pearson product-moment correlation values (below diagonal) and associated p-values (above diagonal) for log-shape ratio skull data across Cercopithecidae.

	PC1	PC2	PC3	PC4	PC5	PC6	PC7	PC8	PC9	PC10	PC11	PC12	PC13	PC14	PC15	PC16	PC17	PC18	PC19	PC20
PC1	1.000	0.265	0.016	0.614	0.072	0.000	0.003	0.012	0.006	0.003	0.000	0.000	0.100	0.016	0.000	0.024	0.000	0.000	0.004	0.000
PC2	-0.255	1.000	0.419	0.013	0.554	0.033	0.534	0.051	0.004	0.250	0.790	0.454	0.503	0.002	0.010	0.015	0.010	0.008	0.676	0.230
PC3	-0.519	-0.186	1.000	0.012	0.156	0.010	0.001	0.901	0.218	0.527	0.004	0.001	0.016	0.639	0.145	0.245	0.428	0.476	0.186	0.005
PC4	0.117	-0.532	0.537	1.000	0.357	0.877	0.083	0.358	0.105	0.639	0.486	0.148	0.205	0.002	0.067	0.817	0.053	0.023	0.537	0.602
PC5	-0.401	0.137	0.321	0.212	1.000	0.030	0.100	0.077	0.435	0.128	0.006	0.011	0.567	0.941	0.403	0.061	0.209	0.142	0.000	0.031
PC6	0.840	-0.466	-0.547	0.036	-0.473	1.000	0.003	0.007	0.000	0.001	0.000	0.000	0.261	0.007	0.000	0.000	0.000	0.001	0.007	0.000
PC7	0.617	0.144	-0.658	-0.387	-0.369	0.617	1.000	0.042	0.043	0.007	0.017	0.000	0.476	0.847	0.053	0.059	0.085	0.238	0.009	0.010
PC8	-0.537	0.432	0.029	-0.211	0.394	-0.566	-0.447	1.000	0.004	0.002	0.022	0.023	0.479	0.136	0.015	0.047	0.000	0.000	0.002	0.006
PC9	-0.577	0.600	0.280	-0.364	0.180	-0.747	-0.446	0.599	1.000	0.009	0.047	0.063	0.668	0.018	0.000	0.039	0.000	0.000	0.244	0.008
PC10	-0.616	0.263	0.146	-0.109	0.343	-0.686	-0.572	0.626	0.554	1.000	0.003	0.005	0.372	0.107	0.003	0.003	0.001	0.013	0.012	0.006
PC11	-0.753	0.062	0.601	0.161	0.576	-0.747	-0.513	0.497	0.439	0.613	1.000	0.001	0.025	0.259	0.002	0.018	0.003	0.002	0.005	0.000
PC12	-0.733	0.173	0.675	0.327	0.541	-0.741	-0.740	0.495	0.413	0.592	0.673	1.000	0.182	0.638	0.040	0.009	0.097	0.097	0.004	0.001
PC13	0.369	0.155	-0.520	-0.289	-0.133	0.257	0.165	0.163	-0.099	-0.205	-0.487	-0.303	1.000	0.707	0.305	0.515	0.582	0.801	0.456	0.202
PC14	-0.519	0.630	-0.109	-0.640	-0.017	-0.573	0.045	0.336	0.512	0.361	0.258	0.109	-0.087	1.000	0.000	0.023	0.002	0.005	0.294	0.094
PC15	-0.799	0.550	0.329	-0.408	0.192	-0.840	-0.429	0.525	0.823	0.611	0.634	0.451	-0.235	0.747	1.000	0.005	0.000	0.000	0.100	0.000
PC16	-0.490	0.523	0.265	0.054	0.415	-0.718	-0.419	0.438	0.454	0.611	0.510	0.555	-0.151	0.492	0.590	1.000	0.023	0.049	0.031	0.001
PC17	0.737	-0.546	-0.183	0.429	-0.286	0.763	0.384	-0.711	-0.752	-0.677	-0.617	-0.372	0.128	-0.641	-0.881	-0.494	1.000	0.000	0.019	0.000
PC18	0.699	-0.564	-0.164	0.495	-0.332	0.679	0.269	-0.755	-0.759	-0.531	-0.627	-0.372	0.059	-0.593	-0.813	-0.434	0.868	1.000	0.059	0.001

PC19	0.599	-0.097	-0.300	-0.143	-0.736	0.570	0.555	-0.641	-0.266	-0.539	-0.589	-0.597	0.172	-0.240	-0.369	-0.472	0.507	0.418	1.000	0.013
PC20	-0.711	0.274	0.593	0.121	0.471	-0.837	-0.549	0.581	0.559	0.583	0.863	0.655	-0.290	0.375	0.704	0.664	-0.719	-0.687	-0.530	1.000

Table S3.3. Pearson product-moment correlation values (below diagonal) and associated p-values (above diagonal) for raw skull data across Cercopithecinae.

	PC1	PC2	PC3	PC4	PC5	PC6	PC7	PC8	PC9	PC10	PC11
PC1	1.000	0.216	0.235	0.001	0.740	0.858	0.001	0.660	0.269	0.405	0.006
PC2	0.385	1.000	0.029	0.961	0.038	0.544	0.106	0.723	0.171	0.232	0.036
PC3	-0.371	-0.628	1.000	0.717	0.099	0.039	0.677	0.310	0.055	0.535	0.021
PC4	0.823	0.016	0.117	1.000	0.766	0.140	0.002	0.295	0.599	0.039	0.305
PC5	-0.107	-0.603	0.499	0.096	1.000	0.665	0.397	0.296	0.395	0.894	0.086
PC6	-0.058	0.195	-0.601	-0.452	0.140	1.000	0.098	0.879	0.139	0.022	0.436
PC7	-0.815	-0.490	0.134	-0.789	0.269	0.500	1.000	0.939	0.762	0.209	0.092
PC8	-0.142	0.115	-0.320	-0.330	-0.329	0.050	0.025	1.000	0.728	0.747	0.872
PC9	0.347	0.423	-0.566	-0.169	-0.271	0.453	-0.098	0.112	1.000	0.062	0.005
PC10	0.265	-0.373	0.199	0.600	-0.043	-0.650	-0.391	0.104	-0.554	1.000	0.521
PC11	-0.735	-0.608	0.653	-0.323	0.516	-0.249	0.508	-0.052	-0.754	0.206	1.000

Table S3.4. Pearson product-moment correlation values (below diagonal) and associated p-values (above diagonal) for log-shape ratio skull data across Cercopithecinae.

	PC1	PC2	PC3	PC4	PC5	PC6	PC7	PC8	PC9	PC10	PC11
PC1	1.000	0.013	0.073	0.346	0.922	0.011	0.039	0.136	0.009	0.049	0.258
PC2	-0.689	1.000	0.001	0.087	0.424	0.000	0.004	0.060	0.223	0.000	0.002
PC3	-0.535	0.832	1.000	0.001	0.335	0.000	0.007	0.041	0.368	0.002	0.001
PC4	0.299	-0.515	-0.809	1.000	0.040	0.012	0.086	0.073	0.800	0.051	0.003
PC5	0.032	-0.255	-0.305	0.597	1.000	0.118	0.143	0.076	0.729	0.057	0.023
PC6	0.701	-0.913	-0.856	0.698	0.476	1.000	0.001	0.008	0.078	0.000	0.001
PC7	-0.600	0.764	0.734	-0.516	-0.449	-0.835	1.000	0.000	0.022	0.000	0.031
PC8	-0.457	0.557	0.595	-0.535	-0.530	-0.720	0.883	1.000	0.017	0.001	0.053
PC9	0.715	-0.380	-0.286	0.082	0.112	0.528	-0.652	-0.670	1.000	0.051	0.677
PC10	-0.577	0.848	0.790	-0.573	-0.561	-0.906	0.959	0.829	-0.574	1.000	0.002

PC11	0.354	-0.797	-0.836	0.770	0.645	0.835	-0.622	-0.571	0.135	-0.791	1.000

Table S3.5. Pearson product-moment correlation values (below diagonal) and associated p-values (above diagonal) for raw skull data across Colobinae.

	PC1	PC2	PC3	PC4	PC5	PC6	PC7	PC8
PC1	1.000	0.001	0.050	0.514	0.248	0.011	0.674	0.210
PC2	-0.891	1.000	0.028	0.481	0.101	0.059	0.414	0.350
PC3	-0.666	0.722	1.000	0.500	0.157	0.006	0.720	0.294
PC4	0.252	-0.271	0.259	1.000	0.123	0.445	0.090	0.274
PC5	0.430	-0.581	-0.514	0.552	1.000	0.682	0.017	0.112
PC6	0.789	-0.648	-0.830	-0.292	0.159	1.000	0.531	0.359
PC7	0.164	-0.312	-0.140	0.597	0.762	-0.242	1.000	0.540
PC8	-0.463	0.354	0.394	-0.410	-0.566	-0.348	-0.236	1.000

Table S3.6. Pearson product-moment correlation values (below diagonal) and associated p-values (above diagonal) for log-shape ratio skull data across Colobinae.

	PC1	PC2	PC3	PC4	PC5	PC6	PC7	PC8
PC1	1.000	0.753	0.612	0.376	0.378	0.752	0.718	0.952
PC2	-0.123	1.000	0.023	0.142	0.250	0.455	0.881	0.618
PC3	0.197	0.738	1.000	0.205	0.767	0.229	0.513	0.989
PC4	-0.336	0.530	0.467	1.000	0.736	0.245	0.328	0.285
PC5	0.335	-0.428	0.116	0.131	1.000	0.784	0.735	0.328
PC6	0.123	-0.287	-0.446	-0.433	-0.107	1.000	0.203	0.703
PC7	-0.141	-0.059	-0.252	0.370	-0.132	0.469	1.000	0.217
PC8	-0.023	-0.193	0.005	-0.401	0.369	0.149	-0.456	1.000

Table S3.7. Pearson product-moment correlation values (below diagonal) and associated p-values (above diagonal) for raw skull data across Papionini.

	PC1	PC2	PC3	PC4	PC5
PC1	1.000	0.886	0.770	0.078	0.021

PC2	-0.076	1.000	0.005	0.249	0.484
PC3	0.155	-0.943	1.000	0.127	0.478
PC4	0.762	-0.559	0.693	1.000	0.039
PC5	0.880	-0.360	0.364	0.834	1.000

Table S3.8. Pearson product-moment correlation values (below diagonal) and associated p-values (above diagonal) for log-shape ratio skull data across Papionini.

	PC1	PC2	PC3	PC4	PC5
PC1	1.000	0.329	0.029	0.285	0.294
PC2	-0.485	1.000	0.853	0.516	0.586
PC3	0.858	-0.099	1.000	0.250	0.304
PC4	-0.525	0.335	-0.558	1.000	0.610
PC5	0.517	0.283	0.508	-0.266	1.000

Table S3.9. Pearson product-moment correlation values (below diagonal) and associated p-values (above diagonal) for raw skull data across Cercopithecini.

	PC1	PC2	PC3	PC4	PC5
PC1	1.000	0.801	0.535	0.011	0.187
PC2	0.134	1.000	0.851	0.705	0.181
PC3	-0.321	0.100	1.000	0.288	0.772
PC4	-0.914	0.199	0.522	1.000	0.086
PC5	-0.622	0.629	0.154	0.749	1.000

Table S3.10. Pearson product-moment correlation values (below diagonal) and associated p-values (above diagonal) for log-shape ratio skull data across Cercopithecini.

	PC1	PC2	PC3	PC4	PC5
PC1	1.000	0.232	0.416	0.274	0.406
PC2	-0.575	1.000	0.385	0.158	0.265
PC3	-0.413	0.438	1.000	0.037	0.419
PC4	0.535	-0.655	-0.839	1.000	0.140

PC5	0.421	-0.544	-0.411	0.677	1.000

Table S3.11. Pearson product-moment correlation values (below diagonal) and associated p-values (above diagonal) for raw skull data across Asian colobines.

	PC1	PC2	PC3	PC4	PC5
PC1	1.000	0.010	0.215	0.814	0.497
PC2	-0.918	1.000	0.100	0.806	0.289
PC3	-0.593	0.729	1.000	0.439	0.199
PC4	0.125	-0.130	0.395	1.000	0.499
PC5	0.350	-0.521	-0.609	0.348	1.000

Table S3.12. Pearson product-moment correlation values (below diagonal) and associated p-values (above diagonal) for log-shape ratio skull data across Asian colobines.

	PC1	PC2	PC3	PC4	PC5
PC1	1.000	0.608	0.464	0.445	0.415
PC2	0.268	1.000	0.817	0.561	0.685
PC3	-0.375	-0.123	1.000	0.333	0.365
PC4	0.390	-0.302	0.482	1.000	0.783
PC5	-0.414	0.213	0.455	-0.145	1.000

Table S3.13. Pearson product-moment correlation values (below diagonal) and associated p-values (above diagonal) for raw skull data across African colobines.

	PC1	PC2
PC1	1	0.430
PC2	0.780	1

Table S3.14. Pearson product-moment correlation values (below diagonal) and associated p-values (above diagonal) for log-shape ratio skull data across African colobines.

	PC1	PC2
PC1	1.000	0.584
PC2	0.608	1.000

Table S3.15. Pearson product-moment correlation values (below diagonal) and associated p-values (above diagonal) for raw cranial data across Cercopithecidae.

	PC1	PC2	PC3	PC4	PC5	PC6	PC7	PC8	PC9	PC10	PC11	PC12	PC13	PC14	PC15	PC16	PC17	PC18	PC19	PC20
PC1	1.000	0.500	0.465	0.067	0.002	0.082	0.002	0.000	0.024	0.004	0.116	0.067	0.757	0.354	0.036	0.014	0.201	0.129	0.784	0.439
PC2	0.156	1.000	0.000	0.000	0.010	0.067	0.044	0.775	0.286	0.291	0.000	0.000	0.025	0.517	0.157	0.778	0.133	0.006	0.114	0.097
PC3	0.168	0.717	1.000	0.002	0.014	0.250	0.542	0.597	0.753	0.288	0.000	0.001	0.291	0.021	0.119	0.908	0.054	0.005	0.573	0.156
PC4	0.408	0.836	0.635	1.000	0.001	0.868	0.036	0.229	0.208	0.506	0.000	0.000	0.081	0.856	0.058	0.773	0.029	0.298	0.712	0.417
PC5	-0.628	-0.546	-0.529	-0.678	1.000	0.316	0.014	0.044	0.985	0.350	0.008	0.000	0.068	0.734	0.268	0.211	0.001	0.580	0.892	0.868
PC6	-0.389	0.407	0.263	0.039	0.230	1.000	0.323	0.115	0.942	0.657	0.330	0.352	0.130	0.166	0.962	0.966	0.178	0.012	0.000	0.001
PC7	0.647	0.443	0.141	0.460	-0.525	0.227	1.000	0.124	0.221	0.118	0.057	0.049	0.083	0.132	0.264	0.048	0.701	0.412	0.053	0.103
PC8	-0.766	-0.066	-0.122	-0.274	0.444	0.354	-0.346	1.000	0.032	0.002	0.189	0.050	0.203	0.120	0.103	0.139	0.691	0.580	0.744	0.364
PC9	0.491	-0.244	-0.073	-0.286	0.004	-0.017	0.279	-0.470	1.000	0.003	0.795	0.773	0.456	0.743	0.646	0.030	0.138	0.681	0.129	0.114
PC10	-0.603	-0.242	-0.243	-0.154	0.215	-0.103	-0.352	0.628	-0.620	1.000	0.135	0.013	0.510	0.274	0.082	0.087	0.728	0.299	0.084	0.001
PC11	-0.353	-0.700	-0.722	-0.732	0.565	-0.223	-0.422	0.298	-0.060	0.337	1.000	0.002	0.244	0.503	0.421	0.180	0.132	0.069	0.078	0.139
PC12	0.407	0.745	0.674	0.703	-0.707	0.214	0.434	-0.433	-0.067	-0.530	-0.638	1.000	0.001	0.441	0.049	0.380	0.141	0.012	0.140	0.009
PC13	-0.072	-0.487	-0.242	-0.390	0.406	-0.341	-0.388	0.290	0.172	0.152	0.266	-0.670	1.000	0.362	0.990	0.989	0.470	0.059	0.046	0.032
PC14	0.213	-0.150	-0.500	0.042	-0.079	-0.314	0.340	-0.350	-0.076	0.250	0.155	-0.178	-0.210	1.000	0.793	0.979	0.650	0.009	0.807	0.106
PC15	0.459	0.320	0.351	0.420	-0.253	-0.011	0.256	-0.366	0.106	-0.389	-0.185	0.434	-0.003	-0.061	1.000	0.412	0.450	0.768	0.924	0.157
PC16	-0.527	0.066	-0.027	-0.067	0.285	-0.010	-0.437	0.334	-0.473	0.383	0.304	-0.202	-0.003	-0.006	-0.189	1.000	0.448	0.351	0.235	0.218
PC17	0.291	0.339	0.426	0.476	-0.662	-0.306	0.089	-0.092	-0.335	0.081	-0.339	0.332	-0.167	-0.105	-0.174	0.175	1.000	0.623	0.051	0.360
PC18	0.342	-0.577	-0.591	-0.238	0.128	-0.537	0.189	-0.128	0.095	0.238	0.405	-0.539	0.419	0.556	0.068	-0.214	-0.114	1.000	0.052	0.023
PC19	0.064	-0.356	-0.131	-0.086	-0.032	-0.710	-0.427	0.076	-0.342	0.386	0.393	-0.333	0.440	0.057	0.022	0.271	0.431	0.430	1.000	0.001
PC20	0.178	0.371	0.321	0.187	-0.039	0.666	0.365	-0.208	0.355	-0.666	-0.334	0.555	-0.468	-0.363	0.320	-0.281	-0.210	-0.493	-0.680	1.000

Table S3.16. Pearson product-moment correlation values (below diagonal) and associated p-values (above diagonal) for log-shape ratio cranial data across Cercopithecidae.

	PC1	PC2	PC3	PC4	PC5	PC6	PC7	PC8	PC9	PC10	PC11	PC12	PC13	PC14	PC15	PC16	PC17	PC18	PC19	PC20
PC1	1.000	0.004	0.000	0.000	0.647	0.002	0.000	0.002	0.002	0.004	0.000	0.001	0.005	0.011	0.036	0.695	0.001	0.888	0.251	0.090
PC2	-0.600	1.000	0.047	0.223	0.387	0.010	0.090	0.765	0.502	0.977	0.071	0.122	0.914	0.152	0.102	0.038	0.339	0.465	0.492	0.162
PC3	-0.837	0.439	1.000	0.000	0.239	0.001	0.000	0.001	0.000	0.001	0.000	0.004	0.025	0.003	0.080	0.881	0.005	0.802	0.139	0.018
PC4	-0.699	0.278	0.758	1.000	0.083	0.006	0.000	0.004	0.007	0.000	0.000	0.008	0.001	0.026	0.477	0.038	0.010	0.431	0.134	0.033
PC5	0.106	0.199	-0.269	-0.387	1.000	0.676	0.187	0.121	0.275	0.004	0.060	0.510	0.036	0.132	0.698	0.008	0.028	0.218	0.214	0.604
PC6	0.637	-0.549	-0.683	-0.581	0.097	1.000	0.083	0.101	0.011	0.384	0.004	0.177	0.693	0.029	0.041	0.860	0.506	0.068	0.822	0.002
PC7	0.823	-0.380	-0.839	-0.710	0.300	0.388	1.000	0.000	0.004	0.000	0.000	0.003	0.000	0.002	0.138	0.889	0.000	0.232	0.062	0.337
PC8	0.630	-0.069	-0.681	-0.599	0.349	0.368	0.772	1.000	0.001	0.001	0.001	0.017	0.000	0.011	0.048	0.130	0.004	0.332	0.415	0.068
PC9	-0.625	0.155	0.769	0.572	-0.250	-0.543	-0.604	-0.674	1.000	0.015	0.005	0.010	0.054	0.085	0.036	0.383	0.035	0.226	0.673	0.002
PC10	-0.601	-0.007	0.672	0.799	-0.595	-0.200	-0.748	-0.663	0.524	1.000	0.000	0.029	0.000	0.005	0.905	0.011	0.000	0.085	0.093	0.373
PC11	0.812	-0.402	-0.824	-0.787	0.418	0.603	0.761	0.677	-0.590	-0.789	1.000	0.054	0.002	0.000	0.084	0.243	0.000	0.300	0.037	0.110
PC12	-0.656	0.348	0.599	0.561	-0.152	-0.306	-0.614	-0.516	0.550	0.477	-0.426	1.000	0.005	0.463	0.149	0.804	0.016	0.523	0.237	0.165
PC13	0.592	-0.025	-0.487	-0.670	0.461	0.092	0.727	0.768	-0.426	-0.790	0.627	-0.589	1.000	0.128	0.235	0.031	0.000	0.011	0.120	0.766
PC14	0.542	-0.324	-0.618	-0.486	0.339	0.477	0.643	0.545	-0.385	-0.591	0.741	-0.170	0.343	1.000	0.337	0.554	0.013	0.584	0.383	0.164
PC15	0.460	-0.367	-0.390	-0.164	-0.090	0.449	0.335	0.436	-0.459	0.028	0.386	-0.326	0.271	0.221	1.000	0.297	0.022	0.850	0.628	0.159
PC16	0.091	-0.456	0.035	0.456	-0.561	-0.041	-0.032	-0.342	0.201	0.544	-0.267	0.058	-0.473	-0.137	0.239	1.000	0.601	0.318	0.849	0.474
PC17	0.649	-0.219	-0.589	-0.549	0.480	0.154	0.764	0.605	-0.461	-0.714	0.746	-0.520	0.771	0.532	0.497	-0.121	1.000	0.013	0.020	0.744
PC18	0.033	0.169	0.058	-0.181	0.280	-0.405	0.273	0.222	0.276	-0.385	0.237	-0.148	0.542	0.127	0.044	-0.229	0.531	1.000	0.000	0.076
PC19	-0.262	0.159	0.334	0.338	-0.283	-0.052	-0.414	-0.188	-0.098	0.377	-0.459	0.270	-0.350	-0.201	-0.112	0.044	-0.504	-0.714	1.000	0.211

PC20	0.379	-0.317	-0.509	-0.468	0.120	0.627	0.220	0.406	-0.631	-0.205	0.359	-0.315	0.069	0.315	0.319	-0.165	0.076	-0.396	0.285	1.000

Table S3.17. Pearson product-moment correlation values (below diagonal) and associated p-values (above diagonal) for raw cranial data across Cercopithecinae.

	PC1	PC2	PC3	PC4	PC5	PC6	PC7	PC8	PC9	PC10	PC11
PC1	1.000	0.750	0.799	0.062	0.015	0.000	0.245	0.001	0.215	0.067	0.453
PC2	-0.103	1.000	0.024	0.004	0.155	0.373	0.708	0.534	0.093	0.688	0.035
PC3	0.082	0.645	1.000	0.346	0.724	0.894	0.005	0.218	0.942	0.577	0.066
PC4	0.553	-0.764	-0.299	1.000	0.001	0.008	0.356	0.391	0.173	0.700	0.078
PC5	-0.679	0.437	0.114	-0.836	1.000	0.001	0.231	0.211	0.489	0.769	0.345
PC6	0.860	-0.283	-0.043	0.725	-0.819	1.000	0.169	0.067	0.783	0.560	0.192
PC7	0.364	0.121	0.754	0.293	-0.374	0.425	1.000	0.136	0.490	0.935	0.989
PC8	0.839	0.200	0.384	0.273	-0.389	0.545	0.456	1.000	0.201	0.054	0.967
PC9	0.386	0.506	0.023	-0.421	0.221	0.089	-0.221	0.397	1.000	0.031	0.988
PC10	-0.545	-0.130	-0.179	0.124	-0.095	-0.187	-0.026	-0.567	-0.621	1.000	0.623
PC11	-0.240	0.610	0.547	-0.527	0.299	-0.405	0.005	-0.013	-0.005	-0.158	1.000

Table S3.18. Pearson product-moment correlation values (below diagonal) and associated p-values (above diagonal) for log-shape ratio cranial data across Cercopithecinae.

	PC1	PC2	PC3	PC4	PC5	PC6	PC7	PC8	PC9	PC10	PC11
PC1	1.000	0.000	0.000	0.012	0.670	0.000	0.000	0.004	0.449	0.178	0.000
PC2	-0.933	1.000	0.007	0.092	0.972	0.011	0.004	0.065	0.755	0.628	0.012
PC3	-0.881	0.733	1.000	0.005	0.282	0.000	0.000	0.002	0.216	0.063	0.001
PC4	-0.698	0.508	0.745	1.000	0.239	0.001	0.035	0.066	0.327	0.004	0.002
PC5	0.138	0.011	-0.339	-0.368	1.000	0.107	0.408	0.479	0.193	0.054	0.237
PC6	0.858	-0.704	-0.889	-0.826	0.489	1.000	0.005	0.013	0.111	0.025	0.000
PC7	0.873	-0.760	-0.927	-0.610	0.263	0.744	1.000	0.004	0.560	0.109	0.014
PC8	-0.755	0.548	0.806	0.547	-0.227	-0.689	-0.759	1.000	0.286	0.069	0.001
PC9	-0.242	0.101	0.385	0.310	-0.403	-0.484	-0.187	0.336	1.000	0.529	0.075
PC10	-0.417	0.156	0.552	0.766	-0.568	-0.640	-0.486	0.542	0.202	1.000	0.032
PC11	0.862	-0.697	-0.833	-0.799	0.369	0.931	0.687	-0.815	-0.533	-0.617	1.000

Table S3.19. Pearson product-moment correlation values (below diagonal) and associated p-values (above diagonal) for raw cranial data across Colobinae.

	PC1	PC2	PC3	PC4	PC5	PC6	PC7	PC8
PC1	1.000	0.361	0.001	0.541	0.236	0.164	0.147	0.664
PC2	0.347	1.000	0.694	0.351	0.005	0.504	0.934	0.709
PC3	-0.895	-0.153	1.000	0.530	0.296	0.255	0.019	0.685
PC4	0.236	-0.353	-0.242	1.000	0.212	0.427	0.790	0.779
PC5	0.440	0.833	-0.392	-0.460	1.000	0.377	0.234	0.495
PC6	0.507	0.257	-0.424	-0.304	0.336	1.000	0.738	0.444
PC7	-0.525	-0.032	0.755	-0.104	-0.442	-0.130	1.000	0.255
PC8	0.169	0.145	-0.158	0.110	0.263	0.293	-0.424	1.000

Table S3.20. Pearson product-moment correlation values (below diagonal) and associated p-values (above diagonal) for log-shape ratio cranial data across Colobinae.

	PC1	PC2	PC3	PC4	PC5	PC6	PC7	PC8
PC1	1.000	0.122	0.639	0.300	0.075	0.980	0.143	0.165
PC2	0.554	1.000	0.681	0.094	0.023	0.800	0.918	0.210
PC3	0.182	0.160	1.000	0.242	0.224	0.002	0.160	0.951
PC4	0.390	0.590	0.435	1.000	0.143	0.383	0.922	0.735
PC5	-0.619	-0.737	-0.450	-0.529	1.000	0.731	0.106	0.077
PC6	-0.010	0.099	0.875	0.332	-0.134	1.000	0.779	0.353
PC7	-0.529	-0.040	-0.511	-0.039	0.574	-0.109	1.000	0.223
PC8	-0.506	-0.462	0.024	-0.132	0.617	0.352	0.451	1.000

Table S3.21. Pearson product-moment correlation values (below diagonal) and associated p-values (above diagonal) for raw cranial data across Papionini.

	PC1	PC2	PC3	PC4	PC5
PC1	1.000	0.438	0.911	0.231	0.333
PC2	0.396	1.000	0.140	0.007	0.230
PC3	0.060	0.676	1.000	0.174	0.126
PC4	0.576	0.930	0.636	1.000	0.059
PC5	-0.482	-0.578	-0.694	-0.795	1.000

Table S3.22. Pearson product-moment correlation values (below diagonal) and associated p-values (above diagonal) for log-shape ratio cranial data across Papionini.

	PC1	PC2	PC3	PC4	PC5
PC1	1.000	0.018	0.009	0.156	0.862
PC2	-0.890	1.000	0.071	0.596	0.707
PC3	0.922	-0.774	1.000	0.233	0.798
PC4	-0.658	0.276	-0.575	1.000	0.810
PC5	0.093	-0.198	-0.135	0.128	1.000

Table S3.23. Pearson product-moment correlation values (below diagonal) and associated p-values (above diagonal) for raw cranial data across Cercopithecini.

	PC1	PC2	PC3	PC4	PC5
PC1	1.000	0.032	0.198	0.075	0.100
PC2	0.850	1.000	0.670	0.280	0.462
PC3	0.610	0.224	1.000	0.309	0.022
PC4	-0.767	-0.530	-0.503	1.000	0.058
PC5	-0.730	-0.377	-0.876	0.796	1.000

Table S3.24. Pearson product-moment correlation values (below diagonal) and associated p-values (above diagonal) for log-shape ratio cranial data across Cercopithecini.

	PC1	PC2	PC3	PC4	PC5
PC1	1.000	0.537	0.246	0.260	0.336
PC2	-0.320	1.000	0.205	0.222	0.055
PC3	0.562	-0.603	1.000	0.014	0.210
PC4	0.548	-0.585	0.902	1.000	0.051
PC5	-0.479	0.801	-0.598	-0.810	1.000

Table S3.25. Pearson product-moment correlation values (below diagonal) and associated p-values (above diagonal) for raw cranial data across Asian colobines.

	PC1	PC2	PC3	PC4	PC5
PC1	1.000	0.041	0.229	0.227	0.086
PC2	-0.829	1.000	0.044	0.058	0.036
PC3	0.579	-0.824	1.000	0.009	0.259

PC4	0.581	-0.796	0.922	1.000	0.414
PC5	-0.750	0.841	-0.549	-0.415	1.000

Table S3.26. Pearson product-moment correlation values (below diagonal) and associated p-values (above diagonal) for log-shape ratio cranial data across Asian colobines.

	PC1	PC2	PC3	PC4	PC5
PC1	1.000	0.193	0.981	0.406	0.024
PC2	-0.616	1.000	0.553	0.156	0.147
PC3	0.013	-0.307	1.000	0.747	0.683
PC4	0.421	-0.657	-0.170	1.000	0.506
PC5	0.870	-0.668	0.215	0.343	1.000

Table S3.27. Pearson product-moment correlation values (below diagonal) and associated p-values (above diagonal) for raw cranial data across African colobines.

	PC1	PC2
PC1	1.000	0.674
PC2	0.491	1.000

Table S3.28. Pearson product-moment correlation values (below diagonal) and associated p-values (above diagonal) for log-shape ratio cranial data across African colobines.

	PC1	PC2
PC1	1.000	0.753
PC2	-0.378	1.000

Table S3.29. Pearson product-moment correlation values (below diagonal) and associated p-values (above diagonal) for raw mandibular data across Cercopithecidae.

	PC 1	PC 2	PC 3	PC 4	PC 5	PC 6	PC 7	PC 8	PC 9	PC 10	PC 11	PC 12	PC 13	PC 14	PC 15	PC 16	PC 17	PC 18	PC 19	PC 20
PC 1	1.000	0.278	0.451	0.000	0.480	0.517	0.011	0.012	0.158	0.004	0.638	0.215	0.303	0.072	0.000	0.000	0.283	0.623	0.111	0.559
PC 2	0.248	1.000	0.245	0.026	0.204	0.000	0.369	0.000	0.545	0.429	0.010	0.003	0.002	0.004	0.876	0.592	0.007	0.134	0.288	0.045
PC 3	-0.174	0.265	1.000	0.813	0.858	0.311	0.310	0.244	0.939	0.167	0.998	0.064	0.529	0.871	0.447	0.808	0.965	0.641	0.805	0.010
PC 4	0.901	0.486	-0.055	1.000	0.174	0.476	0.140	0.002	0.259	0.090	0.776	0.966	0.998	0.021	0.005	0.000	0.826	0.149	0.459	0.191
PC 5	-0.163	-0.289	-0.042	-0.309	1.000	0.131	0.652	0.596	0.799	0.863	0.629	0.069	0.649	0.929	0.335	0.117	0.096	0.273	0.029	0.000
PC 6	-0.150	0.771	0.232	0.164	-0.341	1.000	0.035	0.139	0.151	0.031	0.028	0.003	0.000	0.064	0.334	0.328	0.000	0.255	0.032	0.045
PC 7	0.545	-0.207	0.233	0.333	-0.105	-0.462	1.000	0.735	0.067	0.000	0.033	0.065	0.001	0.767	0.032	0.000	0.024	0.157	0.088	0.086
PC 8	-0.540	-0.699	-0.266	-0.630	-0.123	-0.334	-0.078	1.000	0.992	0.759	0.022	0.167	0.021	0.002	0.196	0.153	0.931	0.180	0.372	0.675
PC 9	-0.320	0.140	-0.018	-0.258	0.059	0.324	-0.408	0.002	1.000	0.018	0.040	0.763	0.010	0.840	0.280	0.017	0.084	0.323	0.810	0.786
PC 10	-0.602	0.182	0.313	-0.379	0.040	0.470	-0.716	0.071	0.511	1.000	0.481	0.097	0.001	0.917	0.104	0.000	0.044	0.146	0.319	0.925
PC 11	-0.109	0.551	0.001	0.066	0.112	0.480	-0.466	-0.497	0.450	0.163	1.000	0.014	0.000	0.257	0.068	0.190	0.217	0.031	0.139	0.714

PC 12	-0.282	0.610	0.412	0.010	-0.404	0.617	-0.410	-0.313	0.070	0.372	0.526	1.000	0.012	0.660	0.005	0.502	0.047	0.053	0.008	0.055
PC 13	-0.236	0.626	0.145	0.000	0.105	0.711	-0.687	-0.501	0.546	0.676	0.717	0.537	1.000	0.063	0.267	0.027	0.040	0.034	0.290	0.777
PC 14	0.401	0.599	0.038	0.499	0.021	0.411	0.069	-0.646	0.047	-0.024	0.259	0.102	0.413	1.000	0.065	0.112	0.839	0.868	0.152	0.682
PC 15	0.757	-0.036	-0.175	0.588	0.221	-0.222	0.469	-0.294	-0.247	-0.365	-0.406	-0.589	-0.254	0.410	1.000	0.004	0.147	0.480	0.008	0.617
PC 16	0.849	0.124	-0.056	0.782	-0.352	-0.224	0.723	-0.323	-0.515	-0.721	-0.298	-0.155	-0.482	0.358	0.606	1.000	0.098	0.796	0.163	0.114
PC 17	-0.246	0.570	0.010	-0.051	-0.373	0.755	-0.491	-0.020	0.385	0.443	0.281	0.437	0.452	0.047	-0.328	-0.370	1.000	0.583	0.011	0.139
PC 18	-0.114	-0.338	-0.108	-0.326	0.251	-0.260	0.320	0.304	-0.227	-0.329	-0.472	-0.428	-0.465	-0.038	0.163	0.060	-0.127	1.000	0.087	0.484
PC 19	0.358	-0.243	0.057	0.171	0.477	-0.468	0.381	-0.205	-0.056	-0.229	-0.334	-0.565	-0.242	0.324	0.563	0.316	-0.544	0.382	1.000	0.103
PC 20	-0.135	-0.441	-0.546	-0.297	0.750	-0.442	-0.384	0.097	0.063	0.022	0.085	-0.425	0.066	-0.095	0.116	-0.356	-0.334	0.162	0.366	1.000

Table S3.30. Pearson product-moment correlation values (below diagonal) and associated p-values (above diagonal) for log-shape ratio mandibular data across Cercopithecidae.

	PC 1	PC 2	PC 3	PC 4	PC 5	PC 6	PC 7	PC 8	PC 9	PC 10	PC 11	PC 12	PC 13	PC 14	PC 15	PC 16	PC 17	PC 18	PC 19	PC 20
PC 1	1.000	0.033	0.978	0.007	0.711	0.865	0.192	0.900	0.012	0.670	0.003	0.001	0.024	0.115	0.085	0.005	0.332	0.036	0.509	0.958
PC 2	-0.467	1.000	0.967	0.054	0.436	0.197	0.202	0.604	0.213	0.906	0.020	0.001	0.021	0.046	0.000	0.319	0.181	0.808	0.402	0.220
PC 3	-0.007	0.010	1.000	0.158	0.028	0.955	0.091	0.021	0.914	0.737	0.815	0.254	0.945	0.410	0.927	0.986	0.320	0.732	0.627	0.062
PC 4	0.568	-0.427	0.319	1.000	0.407	0.551	0.213	0.531	0.302	0.006	0.163	0.004	0.000	0.286	0.093	0.000	0.048	0.225	0.023	0.019
PC 5	0.086	0.180	-0.478	-0.191	1.000	0.402	0.160	0.003	0.214	0.568	0.399	0.115	0.147	0.327	0.138	0.441	0.270	0.329	0.999	0.080
PC 6	0.040	0.293	-0.013	-0.138	0.193	1.000	0.427	0.159	0.092	0.883	0.368	0.566	0.947	0.780	0.851	0.140	0.032	0.885	0.041	0.188
PC 7	0.297	-0.290	-0.379	0.284	-0.318	-0.183	1.000	0.327	0.320	0.554	0.895	0.441	0.031	0.954	0.207	0.051	0.098	0.139	0.421	0.096
PC 8	-0.029	-0.120	0.500	0.145	-0.609	-0.319	0.225	1.000	0.871	0.655	0.653	0.577	0.877	0.260	0.828	0.561	0.950	0.710	0.599	0.099
PC 9	-0.538	0.283	0.025	-0.237	0.283	-0.377	-0.228	0.038	1.000	0.828	0.019	0.005	0.036	0.787	0.011	0.328	0.836	0.029	0.161	0.983
PC 10	0.099	0.027	0.078	0.580	-0.132	-0.034	0.137	0.104	-0.050	1.000	0.829	0.690	0.003	0.797	0.807	0.007	0.756	0.611	0.010	0.627
PC 11	0.619	-0.503	-0.054	0.316	0.194	0.207	-0.031	-0.104	-0.507	-0.050	1.000	0.006	0.527	0.171	0.109	0.473	0.862	0.421	0.057	0.568
PC 12	-0.669	0.687	-0.260	-0.599	0.354	0.133	-0.178	-0.129	0.586	-0.093	-0.583	1.000	0.021	0.385	0.001	0.026	0.099	0.070	0.978	0.349
PC 13	-0.492	0.501	-0.016	-0.739	0.328	0.015	-0.470	-0.036	0.460	-0.612	-0.146	0.501	1.000	0.088	0.012	0.001	0.232	0.233	0.149	0.107
PC 14	0.355	-0.440	-0.190	0.244	0.225	0.065	0.013	-0.257	-0.063	-0.060	0.310	-0.200	-0.382	1.000	0.350	0.755	0.917	0.688	0.828	0.527
PC 15	-0.385	0.743	0.021	-0.376	0.334	0.044	-0.287	0.050	0.542	-0.057	-0.360	0.648	0.539	-0.215	1.000	0.206	0.380	0.054	0.407	0.439
PC 16	0.591	-0.228	-0.004	0.783	-0.178	-0.333	0.431	0.134	-0.224	0.570	0.166	-0.486	-0.672	0.072	-0.287	1.000	0.131	0.050	0.001	0.253
PC 17	0.223	-0.304	0.228	0.435	-0.252	-0.469	0.371	0.014	-0.048	-0.072	0.040	-0.370	-0.272	-0.024	-0.202	0.341	1.000	0.029	0.220	0.001
PC 18	0.460	-0.057	0.079	0.276	-0.224	0.034	0.334	-0.086	-0.477	-0.118	0.185	-0.403	-0.272	-0.093	-0.426	0.432	0.478	1.000	0.477	0.175
PC 19	0.153	0.193	0.113	0.492	0.000	-0.449	0.186	0.122	0.317	0.546	-0.422	-0.006	-0.326	-0.051	0.191	0.673	0.279	0.164	1.000	0.253
PC 20	0.012	0.279	-0.414	-0.506	0.390	0.299	-0.372	-0.370	-0.005	-0.113	0.132	0.215	0.362	0.146	0.178	-0.261	-0.668	-0.308	-0.261	1.000

Table S3.31. Pearson product-moment correlation values (below diagonal) and associated p-values (above diagonal) for raw mandibular data across Cercopithecinae.

	PC1	PC2	PC3	PC4	PC5	PC6	PC7	PC8	PC9	PC10	PC11
PC1	1.000	0.251	0.683	0.000	0.419	0.152	0.061	0.004	0.003	0.006	0.038
PC2	0.359	1.000	0.075	0.059	0.019	0.115	0.084	0.172	0.347	0.503	0.071
PC3	-0.132	0.533	1.000	0.854	0.478	0.578	0.167	0.258	0.860	0.236	0.880
PC4	0.942	0.558	-0.060	1.000	0.211	0.538	0.077	0.007	0.013	0.070	0.044
PC5	-0.258	-0.663	-0.227	-0.389	1.000	0.068	0.073	0.983	0.988	0.750	0.030
PC6	0.440	-0.479	-0.179	0.198	0.543	1.000	0.769	0.142	0.223	0.025	0.823

PC7	0.556	0.519	0.426	0.528	-0.535	0.095	1.000	0.094	0.114	0.135	0.001
PC8	0.757	0.422	0.355	0.728	-0.007	0.450	0.505	1.000	0.042	0.141	0.390
PC9	-0.783	-0.298	0.057	-0.688	0.005	-0.380	-0.480	-0.592	1.000	0.006	0.033
PC10	-0.738	0.215	0.370	-0.540	-0.103	-0.641	-0.458	-0.451	0.743	1.000	0.102
PC11	-0.604	-0.539	-0.049	-0.589	0.625	0.072	-0.844	-0.273	0.617	0.495	1.000

Table S3.32. Pearson product-moment correlation values (below diagonal) and associated p-values (above diagonal) for log-shape ratio mandibular data across Cercopithecinae.

	PC1	PC2	PC3	PC4	PC5	PC6	PC7	PC8	PC9	PC10	PC11
PC1	1.000	0.801	0.861	0.230	0.425	0.000	0.536	0.316	0.036	0.056	0.040
PC2	-0.081	1.000	0.067	0.740	0.708	0.701	0.180	0.376	0.133	0.842	0.212
PC3	-0.057	0.544	1.000	0.074	0.522	0.558	0.327	0.300	0.051	0.235	0.032
PC4	-0.375	-0.107	0.534	1.000	0.519	0.129	0.836	0.931	0.317	0.081	0.236
PC5	0.254	0.121	-0.205	-0.207	1.000	0.151	0.002	0.033	0.930	0.671	0.489
PC6	0.883	-0.124	-0.188	-0.464	0.441	1.000	0.413	0.453	0.106	0.019	0.014
PC7	-0.199	-0.415	-0.310	-0.067	-0.792	-0.261	1.000	0.131	0.341	0.763	0.750
PC8	-0.317	0.281	0.327	-0.028	-0.615	-0.240	0.462	1.000	0.464	0.545	0.402
PC9	0.609	-0.460	-0.574	-0.316	-0.028	0.490	0.301	-0.234	1.000	0.138	0.023
PC10	-0.565	0.065	0.371	0.523	-0.137	-0.662	0.098	0.194	-0.454	1.000	0.018
PC11	0.598	-0.389	-0.618	-0.371	0.222	0.686	0.103	-0.267	0.648	-0.665	1.000

Table S3.33. Pearson product-moment correlation values (below diagonal) and associated p-values (above diagonal) for raw mandibular data across Colobinae.

	PC1	PC2	PC3	PC4	PC5	PC6	PC7	PC8
PC1	1.000	0.053	0.165	0.007	0.267	0.976	0.143	0.000
PC2	-0.660	1.000	0.207	0.135	0.204	0.481	0.203	0.058
PC3	-0.506	0.465	1.000	0.066	0.623	0.030	0.008	0.133
PC4	0.819	-0.538	-0.635	1.000	0.522	0.472	0.104	0.012
PC5	-0.415	0.468	-0.191	-0.247	1.000	0.297	0.542	0.248
PC6	-0.012	-0.271	-0.717	0.276	0.392	1.000	0.122	0.934
PC7	0.530	-0.469	-0.811	0.576	-0.235	0.553	1.000	0.160
PC8	0.920	-0.650	-0.541	0.786	-0.430	-0.032	0.511	1.000

Table S3.34. Pearson product-moment correlation values (below diagonal) and associated p-values (above diagonal) for log-shape ratio mandibular data across Colobinae.

	PC1	PC2	PC3	PC4	PC5	PC6	PC7	PC8
PC1	1.000	0.723	0.585	0.468	0.957	0.181	0.026	0.071
PC2	0.138	1.000	0.395	0.775	0.394	0.981	0.091	0.946
PC3	-0.212	-0.324	1.000	0.457	0.451	0.207	0.425	0.840
PC4	0.278	-0.112	0.285	1.000	0.181	0.951	0.350	0.215
PC5	-0.021	-0.325	-0.289	0.490	1.000	0.280	0.970	0.623
PC6	-0.490	-0.009	0.465	-0.024	-0.405	1.000	0.535	0.826
PC7	-0.728	-0.594	0.305	-0.354	-0.015	0.239	1.000	0.084
PC8	-0.626	0.026	-0.079	-0.458	0.191	-0.086	0.605	1.000

Table S3.35. Pearson product-moment correlation values (below diagonal) and associated p-values (above diagonal) for raw mandibular data across Papionini.

	PC1	PC2	PC3	PC4	PC5
PC1	1.000	0.635	0.128	0.011	0.885
PC2	-0.248	1.000	0.556	0.909	0.097
PC3	-0.692	0.305	1.000	0.062	0.919
PC4	-0.913	-0.060	0.788	1.000	0.566
PC5	-0.077	-0.733	-0.054	0.298	1.000

Table S3.36. Pearson product-moment correlation values (below diagonal) and associated p-values (above diagonal) for log-shape ratio mandibular data across Papionini.

	PC1	PC2	PC3	PC4	PC5
PC1	1.000	0.898	0.798	0.463	0.370
PC2	0.068	1.000	0.734	0.154	0.847
PC3	-0.135	-0.179	1.000	0.440	0.631
PC4	-0.376	-0.660	0.393	1.000	0.310
PC5	-0.450	0.102	-0.251	-0.502	1.000

Table S3.37. Pearson product-moment correlation values (below diagonal) and associated p-values (above diagonal) for raw mandibular data across Cercopithecini.

	PC1	PC2	PC3	PC4	PC5

PC1	1.000	0.270	0.069	0.107	0.905
PC2	-0.539	1.000	0.918	0.087	0.512
PC3	0.776	0.055	1.000	0.353	0.521
PC4	-0.720	0.748	-0.465	1.000	0.822
PC5	-0.063	-0.338	-0.332	-0.119	1.000

Table S3.38. Pearson product-moment correlation values (below diagonal) and associated p-values (above diagonal) for log-shape ratio mandibular data across Cercopithecini.

	PC1	PC2	PC3	PC4	PC5
PC1	1.000	0.326	0.281	0.806	0.196
PC2	0.488	1.000	0.094	0.148	0.013
PC3	-0.529	-0.738	1.000	0.559	0.068
PC4	-0.130	0.667	-0.303	1.000	0.144
PC5	0.612	0.906	-0.779	0.671	1.000

Table S3.39. Pearson product-moment correlation values (below diagonal) and associated p-values (above diagonal) for raw mandibular data across Asian colobines.

	PC1	PC2	PC3	PC4	PC5
PC1	1.000	0.006	0.378	0.128	0.891
PC2	0.939	1.000	0.309	0.048	0.994
PC3	-0.444	-0.503	1.000	0.023	0.086
PC4	-0.692	-0.816	0.873	1.000	0.357
PC5	0.073	0.004	0.750	0.461	1.000

Table S3.40. Pearson product-moment correlation values (below diagonal) and associated p-values (above diagonal) for log-shape ratio mandibular data across Asian colobines.

	PC1	PC2	PC3	PC4	PC5
PC1	1.000	0.762	0.404	0.990	0.190
PC2	0.160	1.000	0.286	0.078	0.741
PC3	0.423	-0.524	1.000	0.032	0.049
PC4	0.007	0.762	-0.849	1.000	0.359
PC5	-0.619	0.174	-0.814	0.460	1.000

Table S3.41. Pearson product-moment correlation values (below diagonal) and associated p-values (above diagonal) for raw mandibular data across African colobines.

	PC1	PC2
PC1	1.000	0.608
PC2	-0.578	1.000

Table S3.42. Pearson product-moment correlation values (below diagonal) and associated p-values (above diagonal) for log-shape ratio mandibular data across African colobines.

	PC1	PC2
PC1	1.000	0.801
PC2	-0.308	1.000

Table S4. Results of the sampling test for covariance matrix estimation using a raw subset of cranial data. Comparative results for the full dataset are provided for comparison.

	**Regression test**				
**Analysis**	**Rejection of Drift?**	**Slope (95% CI)**	**R^2**	**t-statistic**	**p-value**
**Cranial original**					
**Family**					
Cercopithecidae	Yes	1.216 (1.092-1.341)	0.914	3.516	0.001
**Subfamily**					
Cercopithecinae	Yes	1.304 (1.120-1.488)	0.848	3.343	0.002
**Region**					
African colobines	No	1.036 (0.726-1.346)	0.554	0.237	0.814
**Cranial sample test (n=50)**					
**Family**					
Cercopithecidae	Yes	1.203 (1.055-1.350)	0.881	2.783	0.008
**Subfamily**					
Cercopithecinae	Yes	1.277 (1.099-1.455)	0.851	3.152	0.003
**Region**					
African colobines	No	0.994 (0.668-1.321)	0.507	0.036	0.971

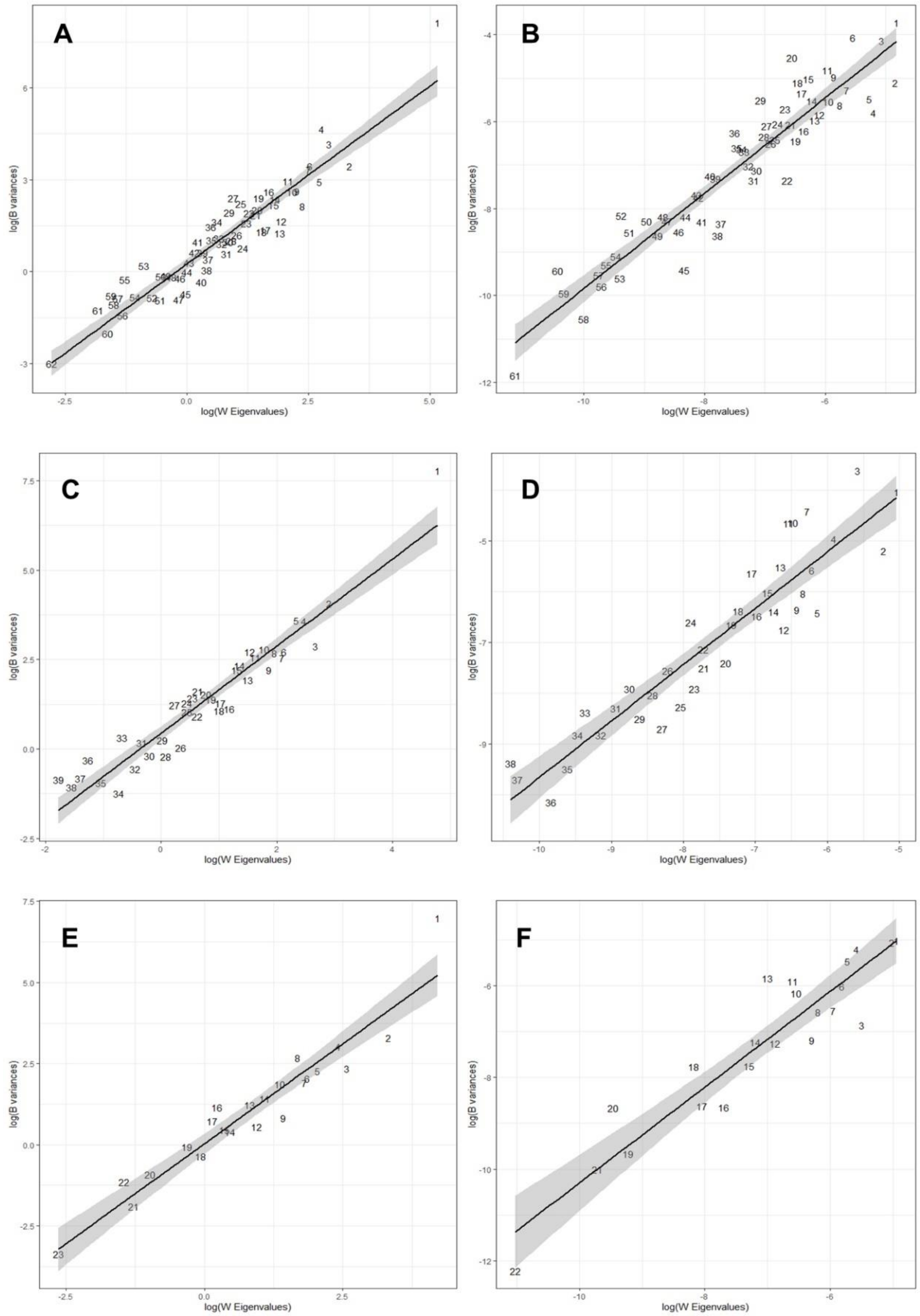

Figure S1. Regression scatterplots of logged between- and within-population variance across Afro-Eurasian monkeys. A) Skull raw data, B) Skull log-shape ratio data, C) Cranial raw data, D) Cranial log-shape ratio data, E) Mandibular raw data, F) Mandibular log-shape ratio data
